# Tuning Co‐Operative Energy Transfer in Copper(I) Complexes Using Two‐Photon Absorbing Diimine‐Based Ligand Sensitizers

**DOI:** 10.1002/anie.202412606

**Published:** 2024-10-31

**Authors:** Noémie Beaucage, Zujhar Singh, Jérémie Bourdon, Shawn K. Collins

**Affiliations:** ^1^ Noémie Beaucage Dr. Zujhar Singh Jérémie Bourdon and Prof. Dr. Shawn K. Collins Department of Chemistry and Centre for Green Chemistry and Catalysis Université de Montréal 1375 Avenue Thérèse-Lavoie-Roux Montréal

**Keywords:** copper, energy transfer, photocatalysis, red light, two photon absoprtion

## Abstract

Photocatalysis mediated by low energy light wavelengths has potential to enable safer, sustainable synthetic methods. A phenanthroline‐derived ligand **bathocupSani**, with a large two‐photon absorption (TPA) cross section was used to construct a heteroleptic complex [Cu(**bathocupSani**)(**DPEPhos**)]BF_4_ and a homoleptic complex [Cu(**bathocupSani**)_2_]BF_4_. The ligand and the respective homoleptic complex with copper exhibit two‐photon upconversion with an anti‐Stokes shift of 1.2 eV using red light. The complex [Cu(**bathocupSani**)_2_]BF_4_ promoted energy transfer photocatalysis enabling oxidative dimerization of benzylic amines, sulfide oxidation, phosphine oxidation, boronic acid oxidation and atom‐transfer radical addition.

## Graphical abstract

Photocatalysis seeing red! A copper‐based complex [Cu(**bathocupSani**)_2_]BF_4_ exhibits two‐photon upconversion promoting energy transfer photocatalysis enabling oxidative dimerization of benzylic amines, sulfide oxidation, phosphine oxidation, boronic acid oxidation and atom‐transfer radical addition.

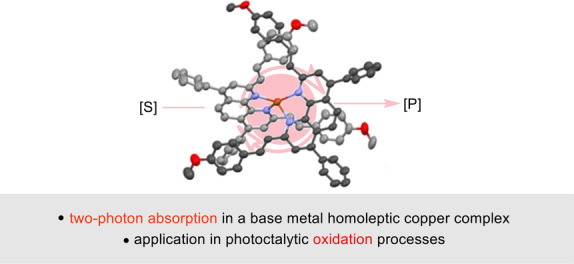

## Introduction

**Figure 1:**
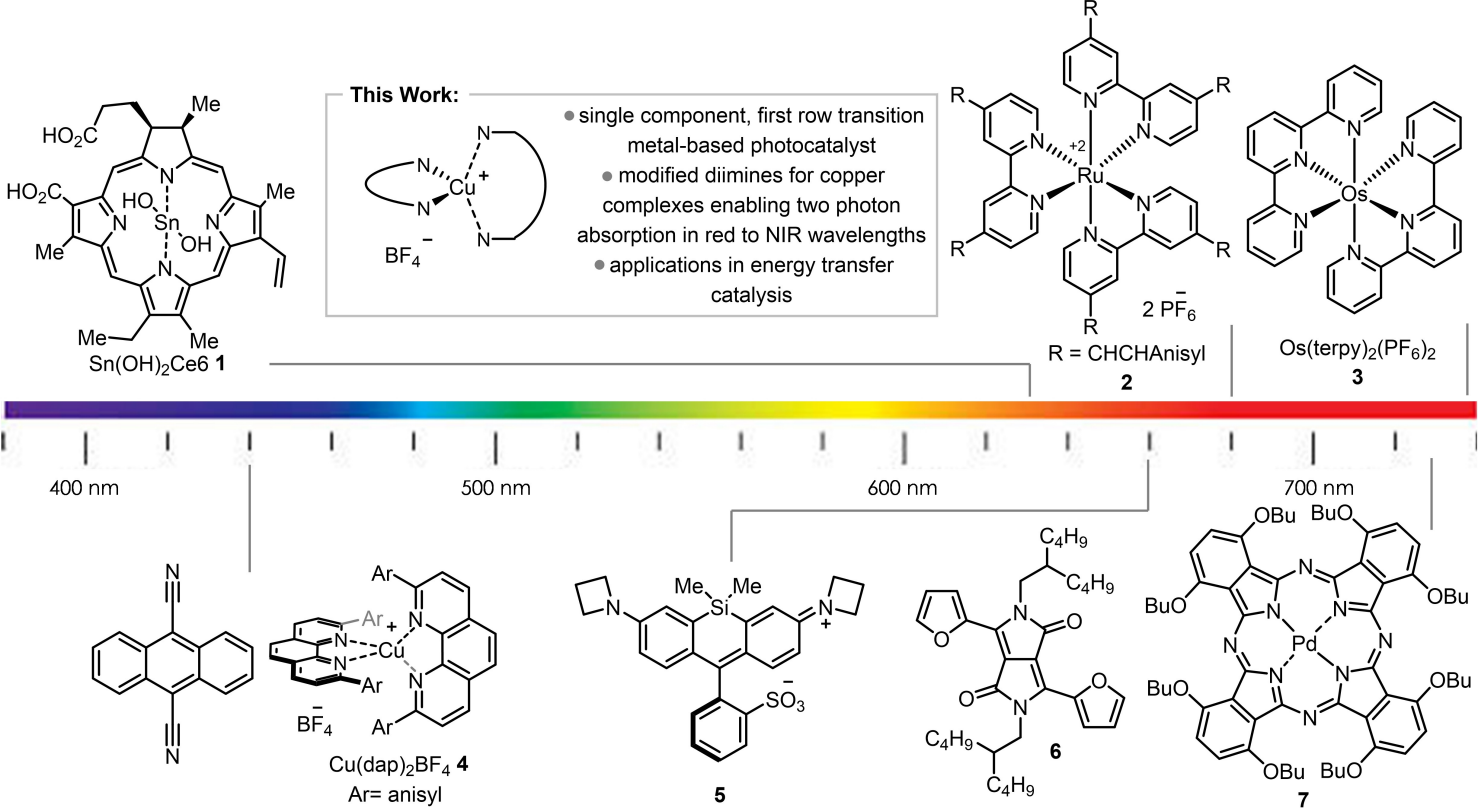
Photocatalysts used to harness lower energy wavelengths for photocatalysis.

Photocatalysis^[1]^ has had a transformative effect on molecular synthesis. The majority of modern photocatalysis makes use of visible light, typically between 390 and 550 nm.^[7]^ With the advent of light‐emitting diode (LED) technology, photocatalysis is considered to be “low energy”, generally less energy‐demanding than analogous thermal reactions. However, irradiation using purple, blue and green light wavelengths using common photocatalysts is actually relatively high energy when one considers just the visible light spectrum. For example, new “high density” blue LEDs in academic laboratories have spurred the use of orange‐tinted lab safety glasses to protect researchers from photooxidative retinal damage. In contrast, red light (600–700 nm) is much lower in energy, poses less health risks, possesses greater potential for sunlight driven photochemistry,^[10]^ and is especially useful in biology. Direct excitation in the red light region has played a role in the development of labelling techniques for capturing biological interactions in living cells.[^11^, ^13^] A red‐light harvesting Sn‐metalated chlorin catalyst **1** (Figure 1) was shown to reduce aromatic azides in vivo for use in proximity labelling,^[14]^ while a silicon Rhodamine dye **5** absorbing at 660 nm could oxidize a dihydrotetrazine to a tetrazine for successful bioconjugation.^[15]^ Similarly, polypyridyl osmium (II) complexes (such as **3**) can absorb in the red to the near infrared (NIR) region and undergo excitation via a spin‐forbidden S0→T1 transition.

Such complexes are active catalysts for a variety of different photochemical processes including a variety of oxidative and reductive syntheses, photopolymerizations, and metallaphotoredox chemistry.^[16]^ Dual photocatalysis excitation involving cooperative effects of two photocatalysts is also a viable route to enabling photochemistry using lower energy wavelengths.^[17]^ Triplet‐triplet upconversion has been demonstrated using palladium and platinum‐based phthalocyanines and appropriate annihilators to achieve chemical transformations with irradiation in the NIR region.^[19]^ An alternative mechanism is to exploit two‐photon absorption (TPA) to allow lower energy light to afford energetically viable photocatalysts. Such approaches remain rare in the area of small molecule photocatalysis. Turro and co‐workers recently reported a Ru complex **2** (Figure 1) with a large TPA cross section whose chromophore could simultaneously absorb two photons in the red, in a single step, to populate the desired excited state.^[20]^ Fine‐tuning of TPA in photocatalysts often encompasses extensive synthetic efforts to modulate the ligands electronics.^[21]^ While examples of two‐photon absorption processes in homogeneous catalysis requiring absorption by multiple components or visible/UV light have begun to appear,^[22]^ most examples of productive TPA processes are found in imaging and photodynamic therapy,^[28]^ and focus on modifying Ru‐based complexes.^[30]^ An alternative for photocatalyst design for TPA would be to examine the potential of analogous copper complexes, which tend to possess similar excited state redox properties and lifetimes to their Ru(II) analogs,^[32]^ and have demonstrated significant potential in photocatalysis.^[33]^ The use of diimine (**NN**) and bisphosphine (**PP**) ligands have afforded homoleptic and heteroleptic copper‐based complexes^18^ as efficient photocatalysts.[^37^, ^42^, ^43^, ^44^, ^51^] Given their ability for broad tuning of photophysical properties, the complexes would seem ideal candidates for further development as red‐light absorbing photocatalysts. Herein, we report on the synthesis and characterization of homoleptic and heteroleptic copper complexes for low energy light photochemistry via two‐photon absorption.

**Figure 2:**
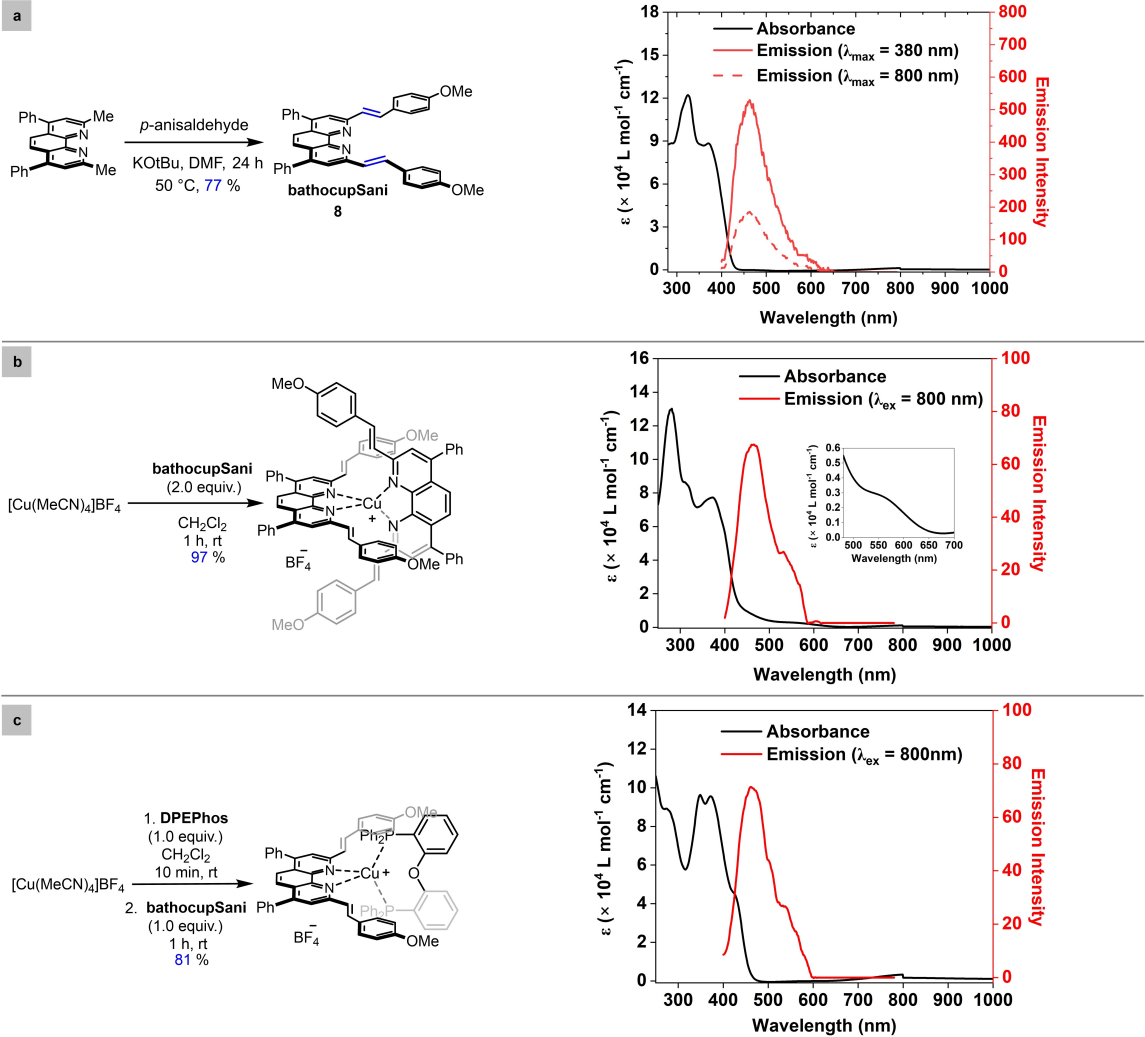
[a] Synthesis of ligand and respective absorption spectra and emission spectra. [b] Synthesis of the homoleptic complex and respective absorption spectra and emission spectra. [c] Synthesis of the heteroleptic complex and respective absorption spectra and emission spectra.

*Ligand design, synthesis and incorporation into homoleptic and heteroleptic complexes*. A commonly used technique to improve the two‐photon absorption cross‐section of organic chromophores is to increase the transition dipole moment from the ground state to the excited state via extension of the π‐conjugated framework.^[52]^ Beginning with the phenanthroline‐derived bathocuproine (**bathocup**), modification of the methyl groups in the 2‐ and 9‐positions allowed for installation of styrenyl (**S**) moieties. It was hoped that introduction of anisyl (**ani**) groups at the ends of the ligands would induce intramolecular charge transfer and a high TPA cross section. Treating bathocuproine with KOtBu and *p*‐anisaldehyde afforded the ligand **bathocupSani** in 77 % yield following purification by recrystallization (Figure 2a). The corresponding homoleptic complex [Cu(**bathocupSani**)_2_]BF_4_ was prepared using established procedures and isolated via filtration as a red solid (97 % yield). An analogous protocol was used for the synthesis of a heteroleptic complex incorporating a **DPEPhos** ligand as a bidentate bisphosphine, isolated as a yellow solid in 81 % yield.

*Characterization of ligands and complexes*. The UV/Vis spectrum of the ligand **bathocupSani** in acetonitrile exhibits two intense bands at approximately 290 nm and 320 nm assigned to π→π* transition of the phenanthroline core (Figure 2a, black trace). In addition, a third band from 330 nm to 430 nm with λ_max_ at 370 nm is observed and can be assigned to intraligand charge transfer (ICT) transitions. The emission spectra of **bathocupSani** when excited at 380 nm exhibited a broad intense emission from 415 nm to 600 nm with λ_max_ at ~470 nm (Figure 2a, red trace). Interestingly, when **bathocupSani** was excited at 800 nm, emission at a similar wavelength is observed (Figure 2a, pink trace). The absorbance spectra of the homoleptic [Cu(**bathocupSani**)_2_]BF_4_ taken at room temperature^[56]^ exhibited broad ^1^MLCT absorption bands centered at 560 nm in addition to the above‐described ligand‐based transitions (Figure 2b, black trace). When the homoleptic complex was excited at 800 nm, the emission also exhibits a λ_max_ at ~470 nm (Figure 2b, red trace). The anti‐Stokes shift of 1.20 eV to achieve the triplet energy of 2.63 eV when excited at 800 nm demonstrates the potential of the **bathocupSani** ligand to populate the excited state of the homoleptic complex's excited triplet state (owing to its small band gap of 2.21 eV). The absence of emission from the copper excited state in the emission spectra demonstrates successful formation of a low energy triplet charge‐separated state. The absorbance spectra of heteroleptic [Cu(**bathocupSani**)(**DPEPhos**)]BF_4_ exhibits a ^1^MLCT transition at 420 nm along with the three ligand centered transitions (Figure 2c, black trace), but lacks the ^1^MLCT absorption bands in the orange or red wavelengths that found in the analogous homoleptic complex. While the emission spectra of the heteroleptic complex does also exhibit emission λ_max_ at ~470 nm like the ligand (**bathocupSani**) emission when excited at 800 nm (Figure 2c, red trace) the energy is not sufficient to populate the excited triplet state of [Cu(**bathocupSani**)(**DPEPhos**)]BF_4_ owing to its large band‐gap energy (3.0 eV).

**Figure 3:**
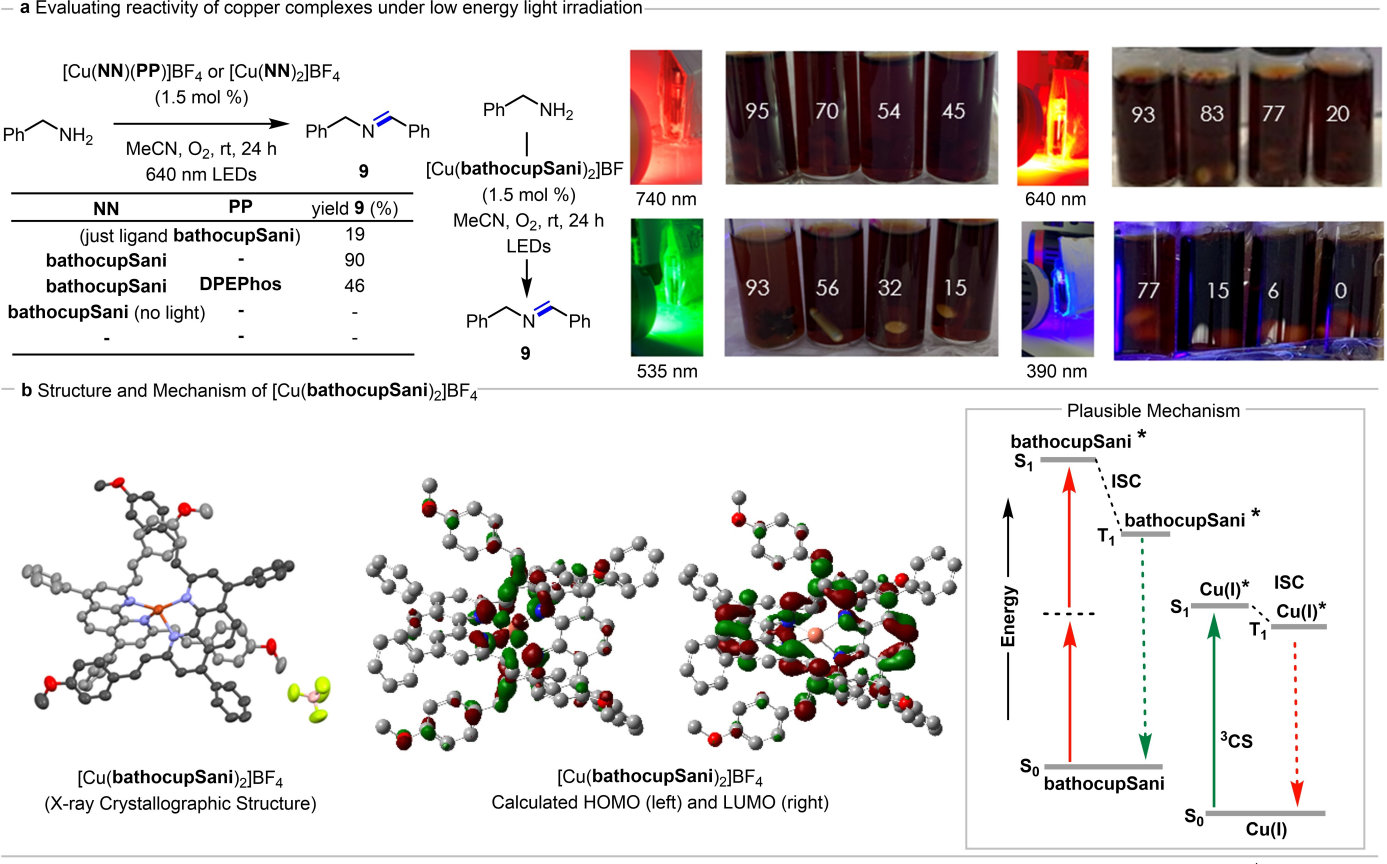
Photocatalysis using copper complexes. [a] Evaluation in the dimerization of benzylic amines. Light penetration tests. Yields determined by ^1^H NMR. [b] X‐crystallographic analysis of [Cu(**bathocupSani**)_2_]BF_4_. Atoms represented as ellipsoids. Hydrogens omitted for clarity. Carbons for each **bathocupSani** ligand shown in different shades of grey for clarity. Carbon=grey. Oxygen=red.Copper=bronze. Nitrogen=purple. Boron=pink. Fluorine=bright green. Calculated HOMO and LUMO of [Cu(**bathocupSani**)_2_]BF4. The ground state optimization and energy calculations were carried out using the B3PW91 functional with dgdzvp as basis set. Excited state calculations were performed using B3LYP as functional with 6–31 g as basis set with acetonitrile as continuous solvent model. (Isovalue=0.03). Schematic of excitation and formation of the triplet charge–separated state using ligand emission energy in homoleptic complex Cu(**bathocupSani**)_2_BF_4_.

*Evaluating the reactivity of copper complexes and possible mechanism for energy‐transfer*. Both homoleptic and heteroleptic copper complexes were evaluated in an oxidative dimerization of benzyl amine to afford imine **9** (Figure 3). The experiments were conducted in thin‐walled tubes under an atmosphere of molecular oxygen and irradiated with a red LED Kessil® lamp. In general, the homoleptic complex [Cu(**bathocupSani**)_2_]BF_4_ afforded higher yields (90 % of **9**) than the corresponding heteroleptic complex [Cu(**bathocupSani**)(**DPEPhos**)]BF_4_ (46 % of **9**). Control reactions showed that no product formed in the absence of catalyst, or light (Figure 3). Only 19 % of the product **9** was observed when the reaction was conducted in presence of ligand only, possibly due to the short excited state lifetimes in many organic chromophores.

An advantage of low energy irradiation and two‐photon absorption is the potential for improved light penetration for photocatalysis. Using the [Cu(**bathocupSani**)_2_]BF_4_ complex, the oxidative dimerization of benzylamine was conducted under four different wavelengths: 390, 535, 640 and 740 nm (Figure 3). For each experiment, four identical vials containing the same reaction mixture were placed one behind the other in front of the LED lamp. To restrict the light path and force the light to penetrate from one vial to the next, a cardboard “shield” was placed along the sides of the four vials. After 24 h of irradiation, the yield of imine **9** was measured by ^1^H NMR. When irradiating from a 390 nm LED lamp, after a 24 h reaction time, the first vial (*ie*. closest to the lamp) had an acceptable yield of 77 % for imine **9**. However, the second vial experienced a significant drop in yield to 15 % of imine **9** and the third and fourth vials had negligible conversion of the benzyl amine. When irradiating at 535 nm, the yields improved for all vials, with conversion and a 15 % yield of imine **9** observed for the fourth vial. Upon irradiation at 640 nm, the first vial provided a 93 % yield as previously observed, however the second and third vial had somewhat similar yields of imine **9** (83 and 77 % respectively). In the fourth vial, the yield of imine **9** increased to 20 % when compared to results with 390 or 535 nm irradiation. Finally, under 740 nm irradiation, high yields of imine **9** were observed in the first vials, but the yield in the fourth vial was the highest observed at 45 %. In summary, the data demonstrates improved light penetration at higher wavelengths.

The complex [Cu(**bathocupSani**)_2_]BF_4_ was crystallized via vapor diffusion of Et_2_O into a CH_2_Cl_2_ solution. X‐ray crystallographic analysis (Figure 3b) exhibited a distorted tetrahedral geometry about the copper center. The styrenyl units of each **bathocupSani** ligand extend over the π‐system of the other ligand. Density functional theory (DFT) was used to calculate the nature of the lowest energy unoccupied molecular orbitals (LUMOs) and the highest energy molecular orbitals (HOMOs) for both complexes (for [Cu(**bathocupSani**)_2_]BF_4_: Figure 3 **b**, for [Cu(**bathocupSani**)(**DPEPhos**)]BF_4_: see Supporting Information). The calculated HOMO of [Cu(**bathocupSani**)_2_]BF_4_ is mainly delocalized on d‐orbitals of the copper metal with minimal contribution from the corresponding ligands, whereas the LUMO is primarily ligand centered. Time‐dependent DFT (TD‐DFT) calculations assisted in assigning the singlet excited‐states of both complexes that were obtained spectroscopically (see Supporting Information). In the case of homoleptic complex, [Cu(**bathocupSani**)_2_]BF_4_, three more prominent states occurring between 595 and 650 nm align with experimental spectra and exhibit transitions from the Cu‐based HOMO to the ligand‐based LUMO confirming the MLCT character of the transitions. In such cases, the copper acted as an electron‐donating unit and the ligand as the electron density‐receiving units.

**Figure 4:**
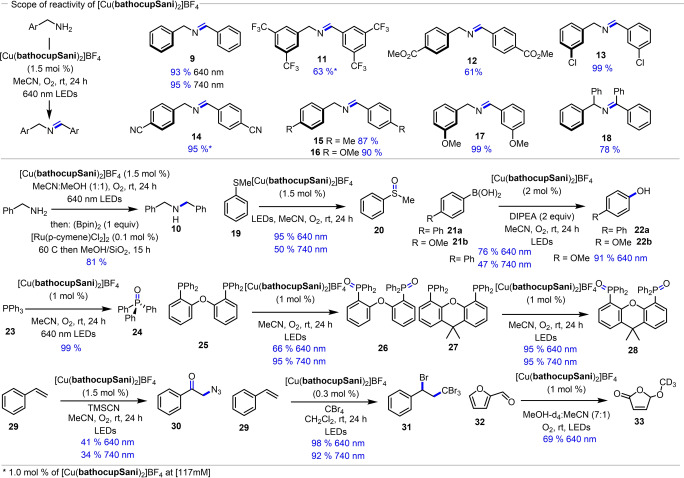
Score of the reactivity for amine dimerization and other oxidation processes (yields determined by ^1^H NMR and/or chromatography).

Given the success with the homoleptic complex to dimerize benzyl amine, further evaluation of the scope of the process was pursued (Figure 4). Benzyl amine underwent dimerization in excellent yields under 640 nm (93 %) or 740 nm (95 %). Benzylic amines containing both electron‐withdrawing groups (**11**, **12, 13** and **14**), and electron‐donating substituents (**15**, **16** and **17**) affording good yields of the corresponding imines. Increasing the steric bulk in proximity to the imine bond slightly decreased yield (**18**). The oxidative dimerization of benzyl amine could also be combined with tandem in situ reduction to afford the secondary amine **10** in 81 % isolated yield. The photocatalytic activity of [Cu(**bathocupSani**)_2_]BF_4_ in ^1^O_2_‐involved benzyl amine coupling prompted us to explore other low energy light‐driven reactions. A 95 % yield of thioanisole sulfoxidation (**19**→**20**) was obtained using the homoleptic catalyst at 640 nm irradiation (Figure 4). The oxidation of boronic acids **21** **a** and **21** **b** to the corresponding phenols **22** **a** and **22** **b** was achieved in 76 % and 91 % isolated yields respectively when irradiated at 640 nm (Figure 4). Phosphine oxidation was also demonstrated: triphenylphosphine underwent quantitative oxidation to Ph_3_PO (**24**), while DPEPhos (**25**) and Xantphos (**27**) could be catalytically oxidized to their corresponding bis‐oxides **26** and **28** in 95 and 66 % yield respectively (similarly high yields were obtained at 740 nm). The atom transfer addition of CBr_4_ to styrene also proceeded in excellent yields under either 640 or 740 nm irradiation (**29**→**31**, 98 and 92 % yield respectively). The synthesis of the α‐azidoketone **30** from styrene, a transformation previously shown to be unique to copper photocatalysis,^[58]^ was also possible with the [Cu(**bathocupSani**)_2_]BF_4_ complex at both 640 and 740 nm. Given that such a process is proposed to proceed via inner‐sphere coordination and participation with Cu(II) species, the successful transformation suggests that the use of the **bathocupSani** ligand can promote varied photochemical processes via two‐photon absorption pathways. The oxidation of furfural via singlet oxygen (**32**→**33**, 69 % yield) was also possible using the [Cu(**bathocupSani**)_2_]BF_4_ complex.

In summary, a commercially available diimine ligand was converted to a new ligand exhibiting two‐photon absorption properties. Isolated via recrystallization, the strategy provides a versatile platform for further derivatization or existing ligand types and exploration of TPA properties. Further, two new copper (I)‐based complexes (homoleptic [Cu(**bathocupSani**)_2_] BF_4_ and heteroleptic [Cu(**bathocupSani**)(**DPEPhos**)] BF_4_) have been synthesized and characterized using the extended π‐conjugated ligand. The homoleptic complex, promoted the oxidative dimerization of benzylic amines, boronic acid oxidation, phosphine oxidation, sulfur oxidation and the α‐oxoazidation of styrene via simultaneous absorption of two photons when excited using 640 nm or 740 nm light in good to excellent yields. Of note, the following demonstrates the first two‐photon absorbing catalyst employing a valuable first row transition metal. The complex can promote processes with mechanisms based on energy transfer, single electron transfer and inner sphere photochemistry. It is highly likely that the ligand design is applicable to the design of other transition metal‐catalyzed photochemical processes that could be enhanced by two‐photon absorption

## Conflict of Interests

The authors declare no conflict of interest.

1

## Supporting information

As a service to our authors and readers, this journal provides supporting information supplied by the authors. Such materials are peer reviewed and may be re‐organized for online delivery, but are not copy‐edited or typeset. Technical support issues arising from supporting information (other than missing files) should be addressed to the authors.

Supporting Information

## Data Availability

The data that support the findings of this study are available in the supplementary material of this article.
