# Supplementary material for: Tuning Co‐Operative Energy Transfer in Copper(I) Complexes Using Two‐Photon Absorbing Diimine‐Based Ligand Sensitizers
Source: Angew Chem Int Ed Engl. 2024 Oct 31;64(8):e202412606. doi: 10.1002/anie.202412606 (PMC11833277; doi:10.1002/anie.202412606)
Supplement: Supplementary file 1 — Supporting Information [file ANIE-64-e202412606-s001.pdf]

Angewandte Chemie
Eine Zeitschrift der Gesellschaft Deutscher Chemiker

Supporting Information

Tuning Co-Operative Energy Transfer in Copper(I) Complexes Using Two-Photon Absorbing Diimine-Based Ligand Sensitizers

*N. Beaucage, Z. Singh, J. Bourdon, S. K. Collins**

SUPPORTING INFORMATION

Tuning Co-operative Energy Transfer in Copper(I) Complexes Using Two-Photon Absorbing Diimine-Based Ligand Sensitizers

Noémie Beaucage, Zujhar Singh, Jérémie Bourdon and Shawn K. Collins*

Département de Chimie, Centre for Green Chemistry and Catalysis, Université de Montréal, 1375 Avenue Thérèse-Lavoie-Roux, Montréal, QC CANADA H2V 0B3

TABLE OF CONTENTS:

GENERAL	S1
EXPERIMENTAL PROCEDURES AND CHARACTERIZATION DATA	S2
GENERAL INFORMATION FOR PHOTOCATALYSIS REACTIONS	S5
GENERAL PROCEDURE FOR PENETRATION LENGTH TEST	S12
ABSORBANCE/EMISSION DATA	S13
ELECTROCHEMICAL DATA	S17
NMR DATA FOR ALL NEW COMPOUNDS	S19
XRAY CRYSTALLOGRAPHY	S43
COMPUTATIONAL DETAILS AND METHODS	S60

GENERAL:

All reactions that were carried out under anhydrous conditions were performed under an inert argon or nitrogen atmosphere in glassware that had previously been dried overnight at 120 °C or had been flame dried and cooled under a stream of argon or nitrogen. All chemical products were obtained from Sigma-Aldrich Chemical Company, Oakwood Chemical or Alfa Aesar and were reagent quality. Technical solvents were obtained from VWR International Co. Anhydrous solvents (CH_2Cl_2, MeCN and DMF) were dried and deoxygenated using a GlassContour system (Irvine, CA). Isolated yields reflect the mass obtained following flash column silica gel chromatography. Organic compounds were purified using silica gel obtained from Silicycle Chemical division (40-63 nm; 230-240 mesh). Analytical thin-layer chromatography (TLC) was performed on glassbacked silica gel 60 coated with a fluorescence indicator (Silicycle Chemical division, 0.25 mm, F254.). Visualization of TLC plate was performed by UV (254 nm), $KMnO_4$, p-anisaldehyde or cerium ammonium molybdate stains. All mixed solvent eluents are reported as %v. Concentration refers to removal of volatiles at low pressure on a rotary evaporator. All reported compounds were homogeneous by thin layer chromatography (TLC) and by ^{1}H NMR. NMR spectra were taken in deuterated $CDCl_3$ or acetone-*d6* using Bruker AV-300 and AV-400 instruments unless otherwise noted. Signals due to the solvent served as the internal standard ($CHCl_3$: δ 7.26 for ^{1}H, δ 77.16 for ^{13}C, Acetone: δ 2.05 for ^{1}H, δ 206.26 for ^{13}C). The acquisition parameters are shown on all spectra. The ^{1}H NMR chemical shifts and coupling constants were determined assuming first-order behavior. Multiplicity is indicated by one or more of the following: s (singlet), d (doublet), t (triplet), q (quartet), m (multiplet), br (broad); the list of couplings constants (J) corresponds to the order of the multiplicity assignment. High resolution mass spectroscopy (HRMS) was done by the Centre régional de spectrométrie de masse at the Département de Chimie, Université de Montréal from an Agilent LC-MSD TOF system using ESI mode of ionization unless otherwise noted.

EXPERIMENTAL PROCEDURES AND CHARACTERIZATION DATA

Synthesis of ligand

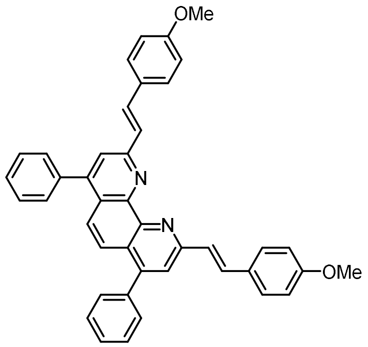

bathocupSani (8): To a 15 mL screw cap vial equipped with a stir bar was added 2,9-dimethyl-4,7-diphenyl 1,10-phenanthroline (bathocup) (500 mg, 1.39 mmol, 1.0 eq.) and p-anisaldehyde (424 mg, 3.05 mmol, 2.2 eq.). A solution of *t*-BuOK (545 mg, 4.86 mmol, 3.5 eq.) in anhydrous DMF (200 mM) was added to the vial. The mixture was stirred at 50 ºC for 20 h. Upon cooling, the reaction mixture was treated with water and following precipitation, the mixture is filtered. The solid residue was dissolved in CH_2Cl_2 and the solution was washed with an aqueous K_2CO_3 solution (2 x 20 mL). The organic phase was dried over $MgSO_4$, filtered and concentrated. Following purification by re-crystallized (DCM/MeOH), the desired product was obtained as an orange solid (639 mg, 77**%).** **^{1}H NMR (400 MHz, $CDCl_3$):** δ 7.86 (s, 2H), 7.78 – 7.72 (m, 6H), 7.67 (d, *J* = 8.9 Hz, 4H), 7.61 – 7.47 (m, 10H), 7.01 – 6.93 (m, 4H), 3.87 (s, 6H). **^{13}C NMR (126 MHz, $CDCl_3$):** δ 160.2, 156.1, 148.9, 138.6, 133.9, 129.8, 129.8, 128.9, 128.8, 128.6, 128.2, 125.9, 123.4, 120.7, 114.5, 55.5. **HRMS (ESI)** m/z calculated for $C_{42}H_{32}N_2O_2$ $[M^+]^+$ 597.2537; found 597.2539.

Synthesis of copper complexes

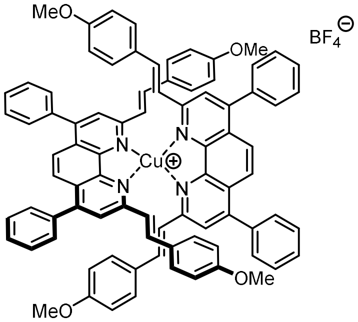

Procedure for the synthesis of homoleptic complex:

Cu(bathocupSani)$_2$BF$_4$: To a stirred solution of $Cu(MeCN)_4BF_4$ (30 mg, 95.5 µmol, 1.0 equiv.) in anhydrous CH_2Cl_2 (5.0 mM) was added bathocupSani (114 mg, 191 µmol, 2.0 equiv.). The reaction was stirred at room temperature for one hour. The reaction mixture was concentrated to approximately a fifth of the original volume and Et_2O was added dropwise to precipitate the product. The desired product was obtained by filtration as a red solid (120 mg, 94**%).** **^{1}H NMR (400 MHz, Acetone-*d6*):** δ 8.34 (s, 4H), 8.21 (s, 4H), 7.83 (d, *J* = 16.5 Hz, 4H), 7.72 – 7.62 (m, 20H), 7.44 (d, *J* = 16.5 Hz, 4H), 6.77 – 6.70 (m, 8H), 6.62 – 6.57 (m, 8H), 3.72 (s, 12H). **^{13}C NMR (101 MHz, Acetone-*d6*):** δ 161.7, 155.5, 150.6, 145.1, 138.0, 137.7, 130.7, 130.3, 130.0, 129.3, 129.2, 127.6,

126.6, 125.1, 123.2, 115.0, 55.8. **HRMS (ESI)** m/z calculated for $C_{84}H_{64}CuN_4O_4$ $[M^+]^+$; 1255.4218 found 1255.4191.

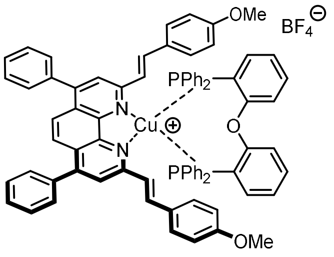

Procedure for the synthesis of the heteroleptic complex:

Cu(bathocupSani)(DPEphos)BF4 : To a stirred solution of $Cu(MeCN)_4BF_4$ (30 mg, 95.5 µmol, 1.0 equiv.) in anhydrous CH_2Cl_2:MeCN (1:1, 12.5 mM) was added Bis[(2-diphenylphosphino)phenyl] ether (DPEphos) (51.5 mg, 95.5 µmol, 1.0 equiv.). The reaction was stirred at room temperature for 90 minutes. Then, to the reaction mixture was added a solution of bathocupSani (57 mg, 95.5 µmol, 1.0 equiv.) in anhydrous CH_2Cl_2 (12.5 mM). The reaction mixture was stirred for an additional four hours. The reaction mixture was concentrated to approximately a fifth of the original volume and Et_2O was added dropwise to precipitate the product. The desired product was obtained by filtration as an orange solid (123 mg, 79%). **^{1}H NMR (400 MHz, Acetone-*d6*) :** δ 8.34 (s, 2H), 7.77 (d, J = 1.6 Hz, 2H), 7.74 – 7.61 (m, 16H), 7.36 (td, J = 7.6, 1.2 Hz, 2H), 7.27 (td, J = 7.8, 1.7 Hz, 2H), 7.18 (q, J = 5.5 Hz, 8H), 7.09 (t, J = 7.4 Hz, 4H), 7.01 – 6.91 (m, 16H), 6.83 – 6.79 (m, 2H), 3.91 (s, 6H). **^{13}C NMR (101 MHz, Acetone-*d6*):** δ 162.05, 156.50, 150.88, 138.83, 138.15, 134.85, 133.91, 133.83, 133.75, 132.91, 130.65, 130.60, 130.12, 129.92, 129.55, 129.30, 129.25, 129.21, 128.48, 127.42, 126.09, 124.53, 122.12, 114.95, 55.96. **HRMS (ESI)** m/z calculated for $C_{78}H_{60}CuN_2O_3P_2$ $[M^+]^+$ 1197.3368; found 1197.3370.

GENERAL INFORMATION FOR PHOTOCATALYSIS

General considerations for the photochemical reactions:

All photocatalysis reactions for reaction optimization were performed in 5-dram vials that were placed in a photoreactor. The vial bottoms were maintained at approximately 7 cm from the Kessil® lamps. An external fan was used to maintain an average temperature below 30 °C.

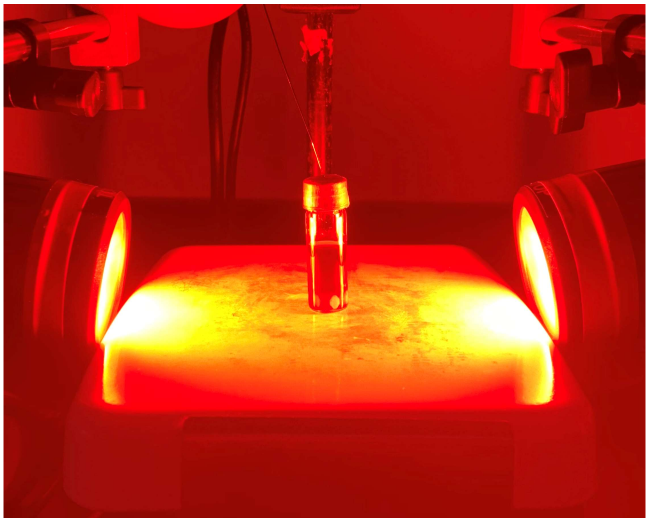

Photoreactor used for the photochemical reactions.

General Procedure for the photochemical oxidation of benzylamine:

In an oven dried appropriate vial, the benzylamine (233 µmol, 1 equiv.) and Cu(**bathocupSani**)$_2$BF$_4$ (1.5 mol %) were dissolved in anhydrous acetonitrile (70 mM) under an oxygen atmosphere. The vial was capped and the solution was irradiated by red Kessil lamps (640 nm) for 24 hours at room temperature. The reaction solvent was then concentrated under vacuum. The NMR yield was determined by adding 1,4-dimethoxybezene as an internal standard.

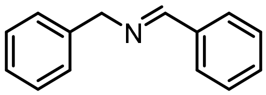

N-Benzylidenebenzylamine (9): Following the general procedure, the product was obtained in 93 % yield (NMR). Spectral data were in accordance with previous report.[1] **^{1}H NMR (400 MHz, CDCl$_3$):** δ 8.37 (s, 1H), 7.79 – 7.70 (m, 2H), 7.43 – 7.34 (m, 3H), 7.33 – 7.28 (m, 4H), 7.24 – 7.18 (m, 1H), 4.79 (d, *J* = 3.4 Hz, 2H).

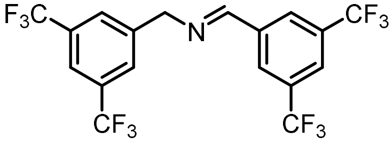

N-(3,5-Bis(trifluoromethyl)benzylidene)-1-(3,5 bis(trifluoromethyl)phenyl) methanamine (11): Following the general procedure, except with copper complex (1.0 mol %) in anhydrous acetonitrile (117 mM), the product was obtained in 63 % yield (NMR). Spectral data were in accordance with previous report.[2] **^{1}H NMR (400 MHz, CDCl$_3$):** δ 8.54 (d, *J* = 1.5 Hz, 1H), 8.26 (d, *J* = 1.8 Hz, 2H), 7.97 (s, 1H), 7.83 (s, 3H), 4.96 (s, 2H).

---

[1] Liu, L.; Zhang, S.; Fu, X.; Yan, C.-H. *Chem. Commun.* **2011**, *47*, 10148-10150.

[2] Zhao, S.; Liu, C.; Guo, Y.; Xiao, J.-C.; Chen, Q.-Y. *J. Org. Chem.* **2014**, *79*, 8926–8931.

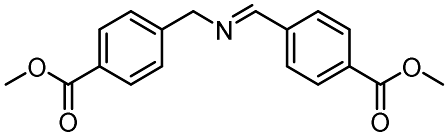

N-(4-methoxycarbonylbenzyl)-N-(4-methoxycarbonylbenzylidene)amine (12): Following the general procedure, the product was obtained in 61 % yield (NMR). Spectral data were in accordance with previous report.[3] **^{1}H NMR (400 MHz, $CDCl_3$):** δ 8.46 (d, *J* = 1.5 Hz, 1H), 8.12 – 8.07 (m, 2H), 8.05 – 8.00 (m, 2H), 7.88 – 7.83 (m, 2H), 7.42 (d, *J* = 8.2 Hz, 2H), 4.90 (s, 2H), 3.94 (s, 3H), 3.91 (s, 3H).

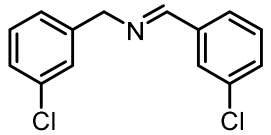

N-(3-chlorobenzylidene)-3-chlorophenylmethylamine (13): Following the general procedure, the product was obtained in 99 % yield (NMR). Spectral data were in accordance with previous report.[2] **^{1}H NMR (400 MHz, $CDCl_3$):** δ 8.34 (s, 1H), 7.82 (t, J = 1.8 Hz, 1H), 7.63 (dt, J = 7.4, 1.4 Hz, 1H), 7.41 (ddd, J = 8.0, 2.1, 1.3 Hz, 1H), 7.37 (d, J = 7.6 Hz, 1H), 7.33 (d, J = 2.4 Hz, 1H), 7.29 (dd, J = 7.8, 6.8 Hz, 1H), 7.26 – 7.20 (m, 2H), 4.79 (s, 2H).

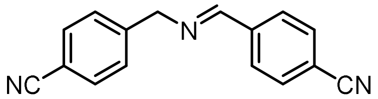

4-(((4-Cyanobenzyl)imino)methyl)benzonitrile (14): Following the general procedure, except with copper complex (1.0 mol %) in anhydrous acetonitrile (117 mM), the product was obtained in 95 % yield (NMR). Spectral data were in accordance with previous report.[4] **^{1}H NMR (400 MHz, $CDCl_3$):** δ 8.45 (s, 1H), 7.89 (d, *J* = 8.2 Hz, 2H), 7.74 – 7.70 (m, 2H), 7.66 – 7.63 (m, 2H), 7.47 (d, *J* = 7.9 Hz, 2H), 4.90 (s, 2H).

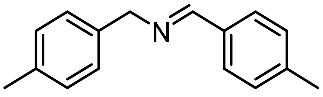

N-(4-Methylbenzylidene)-4-methylbenzylamine (15): Following the general procedure, the product was obtained in 90 % yield (NMR). Spectral data were in accordance with previous report.[1] **^{1}H NMR (400 MHz, $CDCl_3$):** δ 8.35 (s, 1H), 7.68 (d, *J* = 7.9 Hz, 2H), 7.25 – 7.20 (m, 4H), 7.16 (d, *J* = 7.7 Hz, 2H), 4.78 (s, 2H), 2.39 (s, 3H), 2.35 (s, 3H).

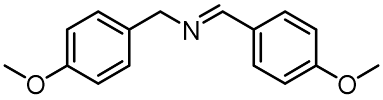

N-(4-Methoxybenzylidene)-4-methoxybenzylamine (16): Following the general procedure, the product was obtained in 90 % yield (NMR). Spectral data were in accordance with previous report.[1] **^{1}H NMR (400 MHz, $CDCl_3$):** δ 8.29 (s, 1H), 7.71 (d, *J* = 8.5 Hz, 2H), 7.27 – 7.19 (m, 2H), 6.93 – 6.86 (m, 4H), 4.72 (s, 2H), 3.83 (s, 3H), 3.79 (s, 3H).

[3] Pal, N. K.; Singh, K; Patra, M.; Yadav, S.; Pandey, P. K.; Bera, J. K. *Green Chem.* **2023**, *25*, 6212-6217.
[4] Yuan, J.-P.; Guan, Z.-J.; Lin, H-Y.; Yan, B.; Liu, K. K.; Zhou, H.-C.; Fang, Y. *Angew. Chem. Int. Ed.* **2023**, *62*, e202303896.

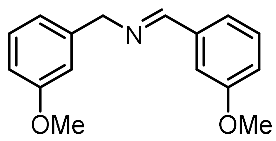

N-(3-methoxybenzylidene)-3-methoxyphenylmethylamine (17): Following the general procedure, the product was obtained in 99 % yield (NMR). Spectral data were in accordance with previous report.[2] **^{1}H NMR (400 MHz, $CDCl_3$):** δ 8.36 (s, 1H), 7.40 (d, J = 1.4 Hz, 1H), 7.35 – 7.29 (m, 2H), 7.27 (d, J = 8.0 Hz, 1H), 6.99 (ddd, J = 7.6, 2.7, 1.7 Hz, 1H), 6.95 – 6.88 (m, 2H), 6.81 (ddd, *J* = 8.2, 2.6, 0.9 Hz, 1H), 4.80 (s, 2H), 3.85 (s, 3H), 3.81 (s, 3H).

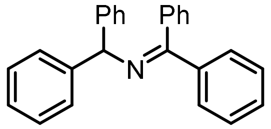

Benzophenone N-benzhydrylimine (18): Following the general procedure, the product was obtained as a white powder in 78 % isolated yield (76 % NMR) by flash column chromatography (10% EtOAc in Hexanes). Spectral data were in accordance with previous report.[1] **^{1}H NMR (400 MHz, $CDCl_3$):** δ 7.77 – 7.73 (m, 2H), 7.48 – 7.26 (m, 12H), 7.26 – 7.24 (m, 2H), 7.22 – 7.16 (m, 2H), 7.10 – 7.06 (m, 2H), 5.55 (s, 1H).

Photochemical oxidation of benzylamine followed by reduction

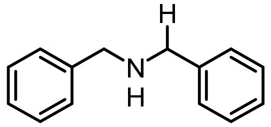

Dibenzylamine (10): Following the general procedure, after the photochemical reaction, pinacolborane (1 equiv.), $[Ru(p\text{-cymene})Cl_2]_2$ (0.1 mol %) were add to the vial. The reaction mixture was heated to 60 °C for 15h. Then the reaction mixture was treated with silica gel and methanol at 50 °C for 6h. The reaction was filtered and purified by flash column chromatography (20% EtOAc in Hexanes) affording the pure product as a white powder in 82 % isolated yield (92 mg). Spectral data were in accordance with a previous report.[5] **^{1}H NMR (400 MHz, $CDCl_3$):** δ 7.46 – 7.29 (m, 10H), 3.83 (s, 4H).

Other photochemical oxidations

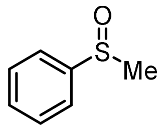

Methyl phenyl sulfoxide (20): In an oven dried vial, the methyl phenylthioether (25 mg, 1 equiv.) and copper complex (1.0 mol %) were dissolved in anhydrous acetonitrile and methanol (3:1, 70 mM) under an oxygen atmosphere. The vial was capped and the solution was irradiated by red Kessil lamps (640 nm) for 24 hours at room temperature. The reaction solvent was then concentrated under vacuum. The NMR yield was determined by adding 1,4-dimethoxybezene as an internal standard. The product was obtained in 95 % yield (NMR). Spectral data were in accordance with previous report.[4] **^{1}H NMR (400 MHz, $CDCl_3$):** δ 7.64 (d, *J* = 7.2 Hz, 2H), 7.54 – 7.45 (m, 3H), 2.73 (s, 3H).

---

[5] Kaithal, A.; Chatterjee, B.; Gunanathan, C. *J. Org. Chem.* **2016**, *81*, 11153-11161.

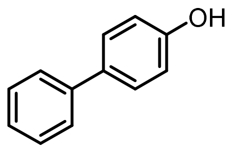

4-Phenylphenol (22): In an oven dried appropriate vial, the 4-biphenylboronic acid (100 mg, 1 equiv.), DIPEA (2 equiv.) and copper complex (2.0 mol %) were dissolved in anhydrous acetonitrile (50 mM) under an oxygen atmosphere. The vial was capped and the solution was irradiated by red Kessil lamps (640 nm) for 24 hours at room temperature. The reaction solvent was then concentrated under vacuum. Purification by flash column chromatography (10% EtOAc in Hexanes) afforded the pure product in 70 % isolated yield (58 mg). Spectral data were in accordance with previous report.[6] **^{1}H NMR (400 MHz, CDCl$_3$):** δ 7.56 – 7.52 (m, 2H), 7.51 – 7.46 (m, 2H), 7.44 – 7.38 (m, 2H), 7.33 – 7.28 (m, 1H), 6.93 – 6.88 (m, 2H), 4.85 (s, 1H).

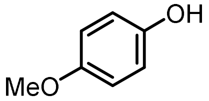

4-Methoxyphenol (22b): Following the general procedure, except under 640 nm Kessil lamp, the product was obtained in 91 % yield (NMR). Spectral data were in accordance with previous report.[12] **^{1}H NMR (400 MHz, CDCl$_3$):** δ 6.82 – 6.71 (m, 4H), 4.75 (s, 1H), 3.76 (s, 3H).

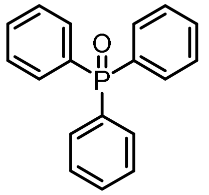

Triphenylphosphine oxide (24): In an oven dried vial, the triphenylphosphine (50 mg, 1 equiv.) and copper complex (1.0 mol %) were dissolved in anhydrous acetonitrile (95 mM) under an oxygen atmosphere. The vial was capped and the solution was irradiated by red Kessil lamps (640 nm) for 24 hours at room temperature. The reaction solvent was then concentrated under vacuum. The NMR yield was determined by adding 1,4-dimethoxybezene as an internal standard. The product was obtained in 94 % yield (NMR). Spectral data were in accordance with previous report.[7] **^{1}H NMR (400 MHz, CDCl$_3$):** δ 7.69 (s, 6H), 7.52 (t, J = 7.1 Hz, 3H), 7.44 (d, J = 7.3 Hz, 6H).

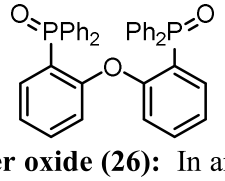

Bis[2-(diphenylphosphino)phenyl]ether oxide (26): In an oven dried appropriate vial, the bis[(2-diphenylphosphino)phenyl] ether (25 mg, 1 equiv.) and copper complex (1.0 mol %) were dissolved in anhydrous acetonitrile (23 mM) under an oxygen atmosphere. The vial was capped and the solution

[6] Yang, S.; Li, H.; Yu, X.; An, J.; Szostak, M. *J. Org. Chem.* **2022**, *87*, 15250−15260.
[7] Tang, L.; Lv, G.; Jia, F.; Zhao, R.; Wang, X.; Zhou Q. *Adv. Synth. Catal.* **2024**, *366*, 70.

was irradiated by red Kessil lamps (640 nm) for 24 hours at room temperature. The reaction solvent was then concentrated under vacuum. The pure product was obtained in 66 % isolated yield (17.5 mg). Spectral data were in accordance with previous report.[8] **^{1}H NMR (400 MHz, CDCl$_3$):** δ 7.77 – 7.57 (m, 10H), 7.54 – 7.27 (m, 9H), 7.24 (s, 3H), 7.19 – 7.04 (m, 4H), 6.03 (ddd, J = 8.2, 5.0, 1.1 Hz, 2H).

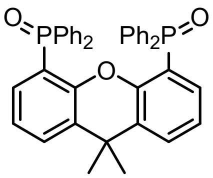

(9,9-dimethyl-9H-xanthene-4,5-diyl) bis(diphenylphosphineoxide) (28): In an oven dried appropriate vial, the 4,5-bis(diphenylphosphino)-9,9-dimethylxanthene (25 mg, 1 equiv.) and copper complex (1.0 mol %) were dissolved in anhydrous acetonitrile (21 mM) under an oxygen atmosphere. The vial was capped and the solution was irradiated by red Kessil lamps (640 nm) for 24 hours at room temperature. The reaction solvent was then concentrated under vacuum. The NMR yield was determined by adding 1,4-dimethoxybezene as an internal standard. The product was obtained in 95 % yield (NMR). Spectral data were in accordance with previous report.[9] **^{1}H NMR (400 MHz, CDCl$_3$):** δ 7.40 (dd, *J* = 7.8, 1.5 Hz, 2H), 7.25 – 7.14 (m, 20H), 6.95 (dd, *J* = 7.6, 7.6 Hz, 2H), 6.54 (dd, *J* = 7.5, 1.7 Hz, 2H), 1.65 (s, 6H).

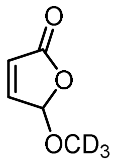

5-(Methoxy-d3)furan-2(5H)-one (33): In 750 uL of a 7:1 MeOH-d4:acetonitrile solution is added 23.9 mg (248 μmol) of freshly purified furfural and 3.34 mg (1 mol%) of the copper complex. The solution is then bubbled with O_2 for 5 minutes before stirring under red light (640 nm) irradiation. Spectral data were in accordance with previous report.[10] **1H NMR** (400 MHz, MeOH-d4) δ 7,39 - 7,38 (dd, *J*=4, 1H), 6,23 – 6,19 (m, 2 H).

Other photochemical reactions

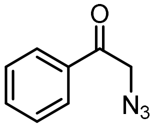

2-Azido-1-phenylethan-1-one (30): In an oven dried appropriate vial, the 2-azido-1-phenylethan-1-one (50 mg, 1 equiv.) and copper complex (1.0 mol %) were dissolved in acetonitrile (2 mL) followed by the addition of trimethylsilylazide (2 equiv.) under air. The solution was irradiated by red Kessil lamps (640 nm) for 24 hours at room temperature. Upon completion (followed by TLC), the reaction mixture was diluted in dichloromethane in a round bottom flask and then concentrated under vacuum. Purification by flash column chromatography (10% EtOAc in Hexanes) afforded the pure product in 41 % isolated yield (33 mg). Spectral data were in accordance with previous report.[11]

[8] Yin, K.; Wei, M.; Wang, Z.; Luo, W.; Li, L. *Org. Lett.* **2023**, *25*, 5236–5241.
[9] Hiramatsu, R.; Horibe, T.; Ishihara, K.; Sakakibara, M.; Takeda, K. *Angew. Chem. Int. Ed.* **2020**, 59, 16470 –16474.
[10] Ravetz, B. D.;Tay, N. E. S.; Joe, C. L.; Sezen-Edmonds, M.; Schmidt, M. A.; Tan, Y.; Janey. J. M.; Eastgate, M. D.; Rovis, T. *ACS Cent. Sci.* **2020**, *6*, 2053–2059.
[11] Wei, W.; Cui, H. H.; Yue, H. L.; Yang, D. S. *Green. Chem.* **2018**, 20, 3197-3202.

^{1}**H NMR (400 MHz, CDCl$_3$):** δ 7.91 (dd, *J* = 8.4, 1.4 Hz, 2H), 7.67 – 7.59 (m, 1H), 7.51 (dd, *J* = 8.4, 7.1 Hz, 2H), 4.57 (s, 2H).

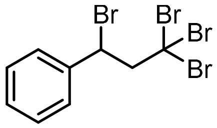

(1,3,3,3-Tetrabromopropyl)benzene (31): In an oven dried appropriate vial, the styrene (158 mg, 1 equiv.), carbon tetrabromide (1 equiv.) and copper complex (0.3 mol %) were dissolved in dichloromethane (1.5 mL) under nitrogen. The solution was irradiated by red Kessil lamps (640 nm) for 24 hours at room temperature. Upon completion (followed by TLC), the reaction mixture was purified on silica gel (5% EtOAc in Hexanes) afforded the pure product in 98 % isolated yield (644 mg). Spectral data were in accordance with previous report.[12] ^{1}**H NMR (400 MHz, CDCl$_3$):** δ 7.52 – 7.46 (m, 2H), 7.40 – 7.28 (m, 3H), 5.33 (dd, *J* = 7.7, 4.1 Hz, 1H), 4.16 – 4.02 (m, 2H).

Photochemical oxidation at 740 nm

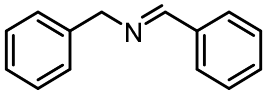

N-Benzylidenebenzylamine (9): Following the general procedure, except under 740 nm Kessil lamp, the product was obtained in 95 % yield (NMR). Spectral data were in accordance with previous report.[1] ^{1}**H NMR (400 MHz, CDCl$_3$):** δ 8.37 (s, 1H), 7.79 – 7.70 (m, 2H), 7.43 – 7.34 (m, 3H), 7.33 – 7.28 (m, 4H), 7.24 – 7.18 (m, 1H), 4.79 (d, *J* = 3.4 Hz, 2H).

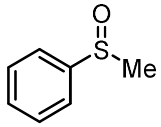

Methyl phenyl sulfoxide (20): Following the general procedure, except under 740 nm Kessil lamp, the product was obtained in 49 % yield (NMR). Spectral data were in accordance with previous report.[3] ^{1}**H NMR (400 MHz, CDCl$_3$):** δ 7.64 (d, J = 7.2 Hz, 2H), 7.54 – 7.45 (m, 3H), 2.73 (s, 3H).

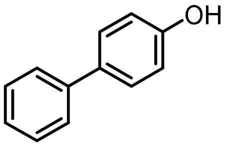

4-Phenylphenol (22a): Following the general procedure, except under 740 nm Kessil lamp, the product was obtained in 47 % yield (NMR). Spectral data were in accordance with previous report.[7] ^{1}**H NMR (400 MHz, CDCl$_3$):** δ 7.56 – 7.52 (m, 2H), 7.51 – 7.46 (m, 2H), 7.44 – 7.38 (m, 2H), 7.33 – 7.28 (m, 1H), 6.93 – 6.88 (m, 2H), 4.85 (s, 1H).

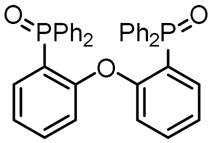

Bis[2-(diphenylphosphino)phenyl]ether oxide (26): Following the general procedure, except under 740 nm Kessil lamp, the product was obtained in 95 % yield (NMR). Spectral data were in

[12] Pirtsch, M.; Paria, S.; Matsuno, T.; Isobe, H.; Reiser, R. *Chem. Eur. J.* **2012**, 18, 7336–7340.

accordance with previous report.[8] **^{1}H NMR (400 MHz, $CDCl_3$):** δ 7.77 – 7.57 (m, 10H), 7.54 – 7.27 (m, 9H), 7.24 (s, 3H), 7.19 – 7.04 (m, 4H), 6.03 (ddd, J = 8.2, 5.0, 1.1 Hz, 2H).

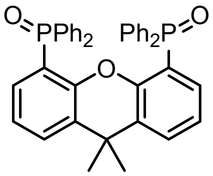

(9,9-Dimethyl-9H-xanthene-4,5-diyl) bis(diphenylphosphineoxide) (28): Following the general procedure, except under 740 nm Kessil lamp, the product was obtained in 95 % yield (NMR). Spectral data were in accordance with previous report.[9] **^{1}H NMR (400 MHz, $CDCl_3$):** δ 7.40 (dd, *J* = 7.8, 1.5 Hz, 2H), 7.25 – 7.14 (m, 20H), 6.95 (dd, *J* = 7.6, 7.6 Hz, 2H), 6.54 (dd, *J* = 7.5, 1.7 Hz, 2H), 1.65 (s, 6H).

Other photochemical reactions at 740 nm

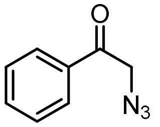

2-Azido-1-phenylethan-1-one (30): Following the general procedure, except under 740 nm Kessil lamp, the product was obtained in 34 % yield (NMR). Spectral data were in accordance with previous report.[10] **^{1}H NMR (400 MHz, $CDCl_3$):** δ 7.91 (dd, *J* = 8.4, 1.4 Hz, 2H), 7.67 – 7.59 (m, 1H), 7.51 (dd, *J* = 8.4, 7.1 Hz, 2H), 4.57 (s, 2H).

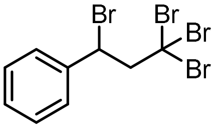

(1,3,3,3-Tetrabromopropyl)benzene (31): Following the general procedure, except under 740 nm Kessil lamp, the product was obtained in 92 % isolated yield (600 mg). Spectral data were in accordance with previous report.[11] **^{1}H NMR (400 MHz, $CDCl_3$):** δ 7.52 – 7.46 (m, 2H), 7.40 – 7.28 (m, 3H), 5.33 (dd, *J* = 7.7, 4.1 Hz, 1H), 4.16 – 4.02 (m, 2H).

GENERAL PROCEDURE FOR PENETRATION LENGTH TEST

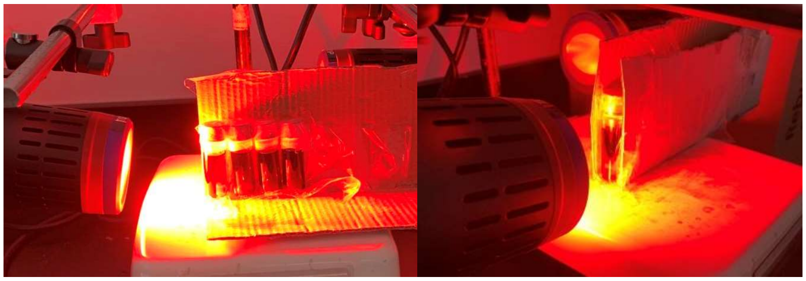

Penetration length test set-up

Following the general procedure, 4 vials of the photochemical oxidation of benzylamine were prepared. The vials were aligned with the corresponding Kessil lamp (740 nm, 640 nm, 535 nm or 390 nm) (as shown above). The sides of the vials were covered with cardboard to limit penetration of light from an other angles and the Kessil lamp was placed facing only the exposed face of the front vial. The NMR yields were determined by adding 1,4-dimethoxybezene as an internal standard to the crude product. Spectral data for the product **9** were in accordance with a previous report.[1] **^{1}H NMR (400 MHz, $CDCl_3$):** δ 8.37 (s, 1H), 7.79 – 7.70 (m, 2H), 7.43 – 7.34 (m, 3H), 7.33 – 7.28 (m, 4H), 7.24 – 7.18 (m, 1H), 4.79 (d, *J* = 3.4 Hz, 2H).

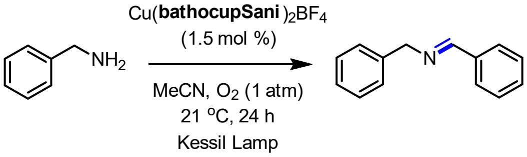

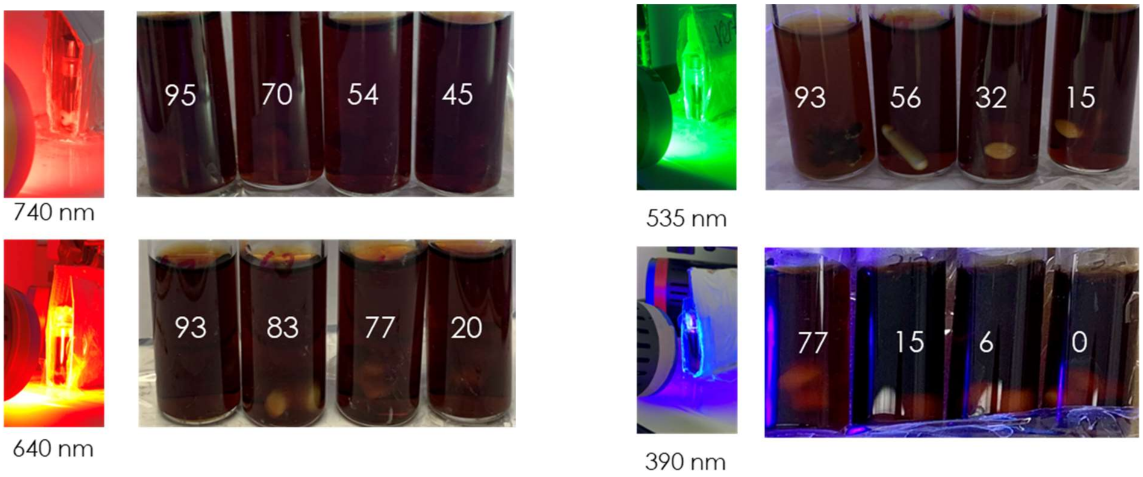

ABSORBANCE/EMISSION DATA

Absorbance UV-Vis spectra were recorded with Varian Cary 5000 UV-Vis-NIR spectrophotometer in a quartz cuvette. Emission spectra were recorded with the Varian Cary Eclipse Fluorescence. Spectrophotometer/Fluorometer in a quartz cuvette.

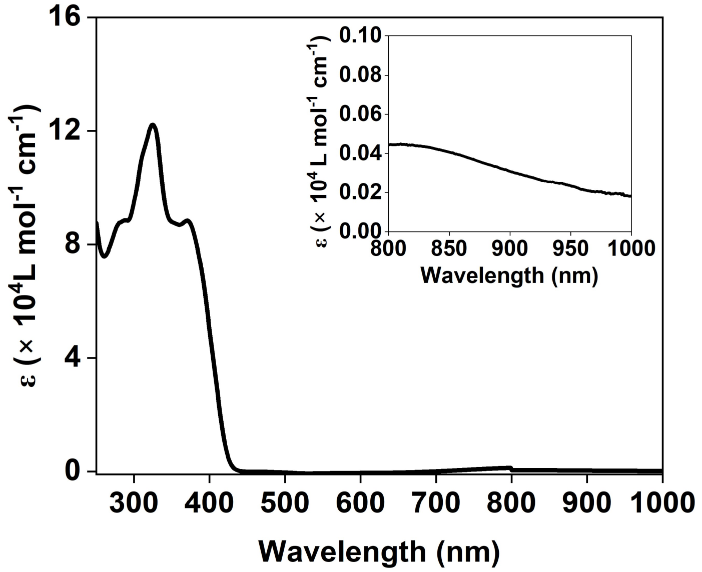

Figure S1: UV-visible absorption spectrum of **bathocupSani** recorded at ambient temperature in CH_3CN ($1.0 \cdot 10^{-5}$M).

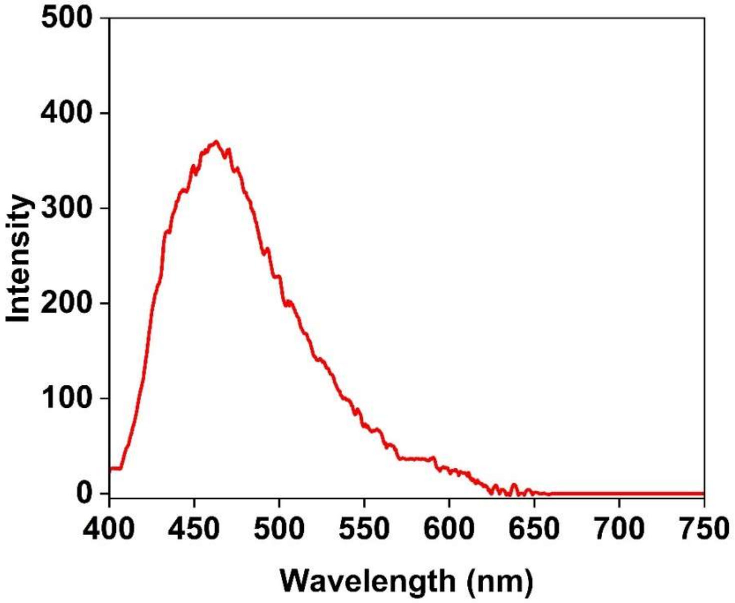

Figure S2: Emission spectrum of **bathocupSani** excited at 395 nm, recorded at ambient temperature in CH_3CN ($1.0 \cdot 10^{-5}$M).

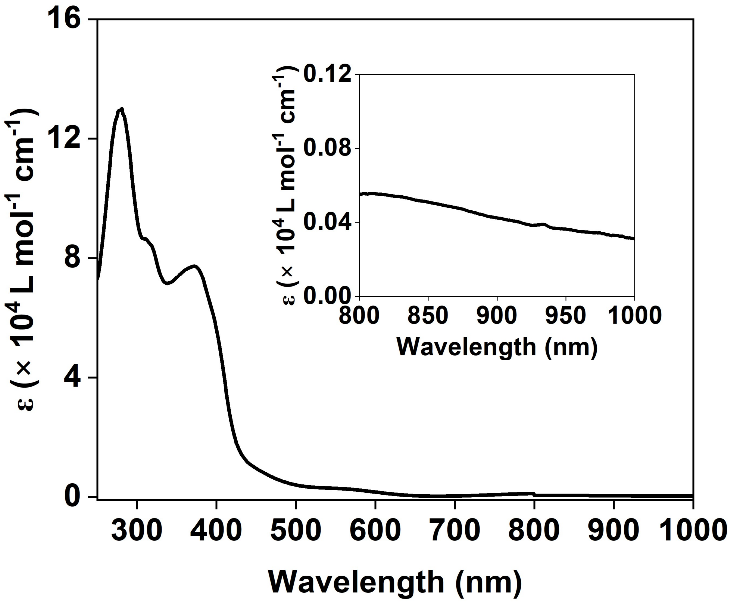

Figure S3: UV-visible absorption spectrum of Cu(**bathocupSani**)$_2$BF$_4$ complex recorded at ambient temperature in CH_3CN ($1.0 \cdot 10^{-5}$M).

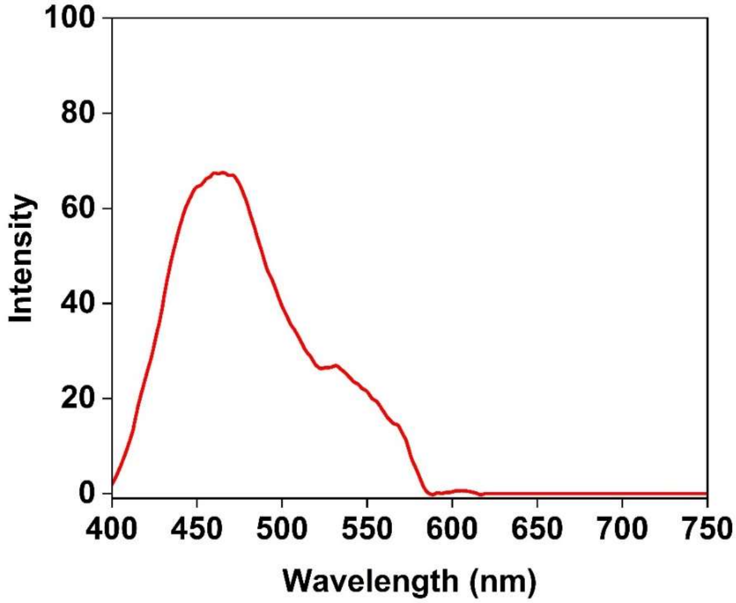

Figure S4: Emission spectrum of Cu(**bathocupSani**)$_2$BF$_4$ complex excited at 395 nm, recorded at ambient temperature in CH_3CN ($1.0 \cdot 10^{-5}$M).

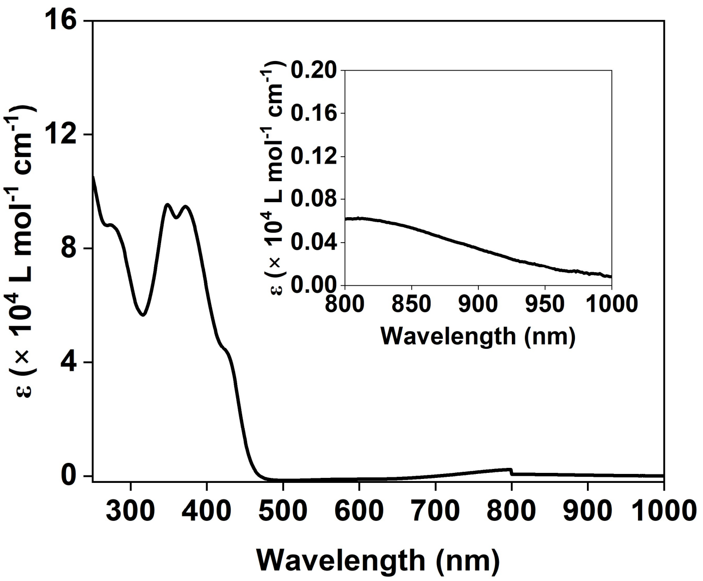

Figure S5: UV-visible absorption spectrum of Cu(**bathocupSani**)(**DPEPhos**)BF_4 complex recorded at ambient temperature in CH_3CN ($1.0 \cdot 10^{-5}$M).

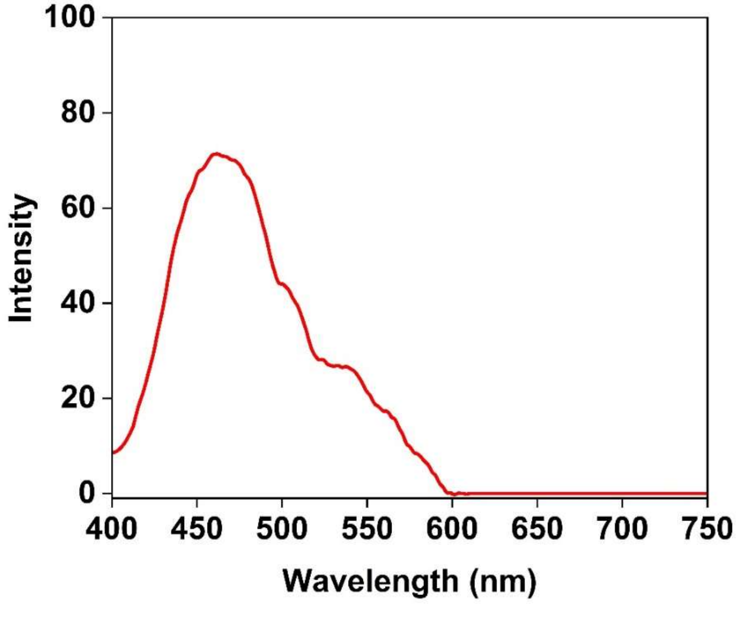

Figure S6: Emission spectrum of Cu(**bathocupSani**)(**DPEPhos**)BF_4 complex recorded at ambient temperature in CH_3CN ($1.0 \cdot 10^{-5}$M).

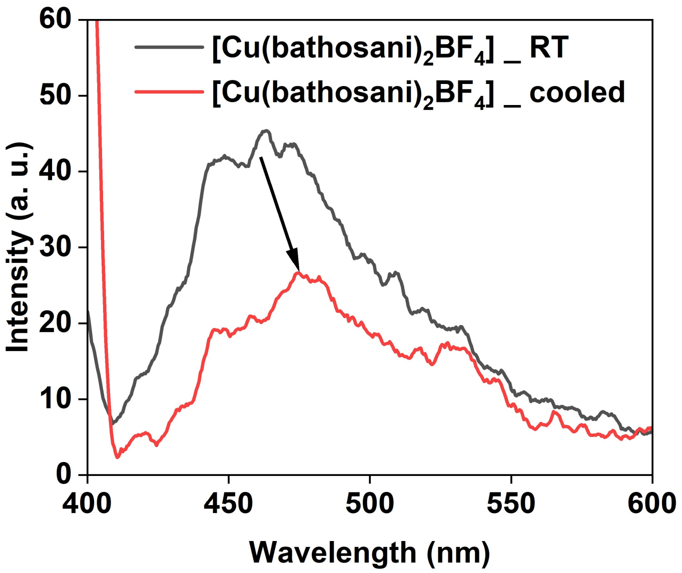

Figure S7. Emission scan of homoleptic complex [Cu(bathocupSani)$_2$]BF_4 at room temperature and as a solution cooled in liquid nitrogen. After recording the emission at room temperature, the vial containing the solution was cooled in liquid nitrogen and the spectra measured. The precise temperature of the cooled solution was able to be accurately measured. The emission intensity decrease may be due to change in transmittance of the solution due to some condensed water vapours around the vial whereas red-shift is representative of TADF.

ELECTROCHEMICAL DATA

Cyclic voltammetry measurements in dry acetonitrile (MeCN) with tetra-*n*-butylammonium hexafluorophosphate ($TBAPF_6$) as the supporting electrolyte were performed using a standard three-electrode cell, consisting of a silver wire pseudo-reference electrode, a platinum wire counter electrode and a glassy carbon disk working electrode with a BioLogic SP-50 potentiostat. The solutions were degassed with a flow of argon for 10 minutes prior to the measurements, which was left over the surface of the solution during the measurements. All measurements were carried out at 22 °C. All potentials were reported to the mid-point potential of the ferrocene/ferrocenium (Fc/Fc^+) redox couple which was determined in the aforementioned electrolyte.

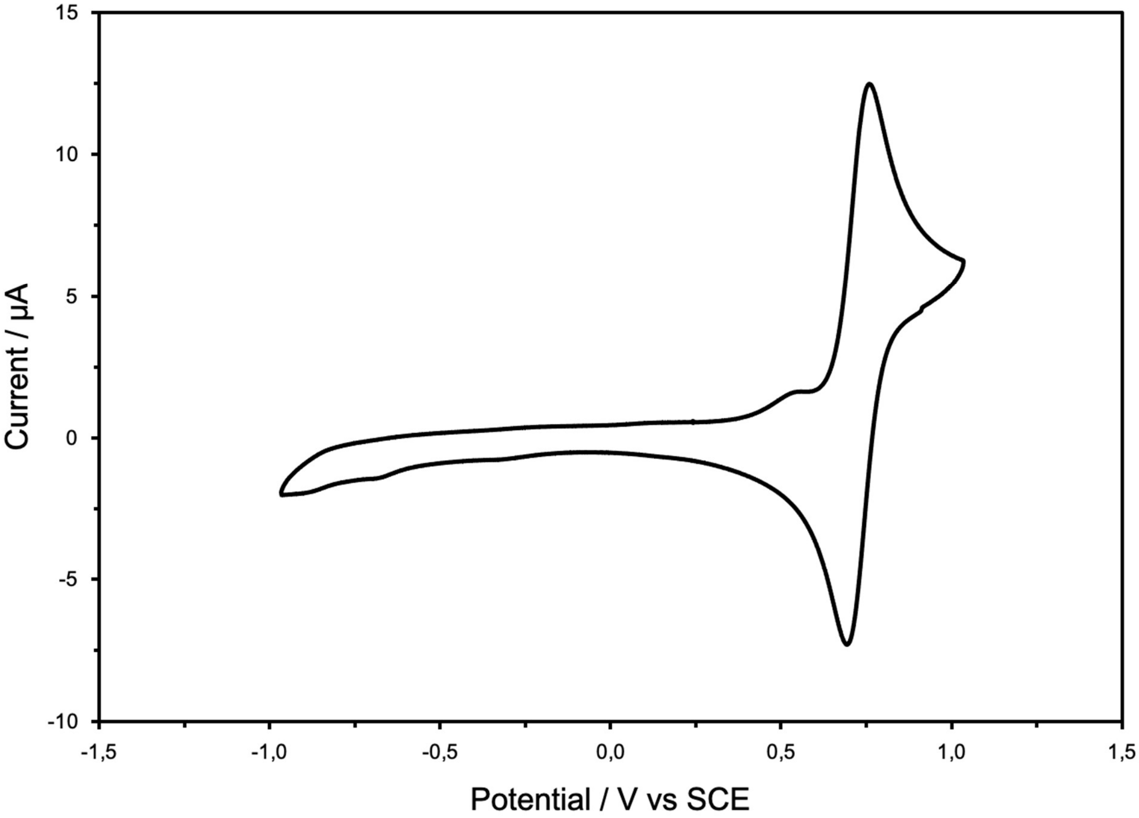

Figure S8: Cyclic Voltammogram of Cu(**bathocupSani**)$_2BF_4$ in MeCN (1 mM) using $TBAPF_6$ (100 mM) as supporting electrolyte at a scan rate of 50 $mV{\cdot}s^{-1}$.

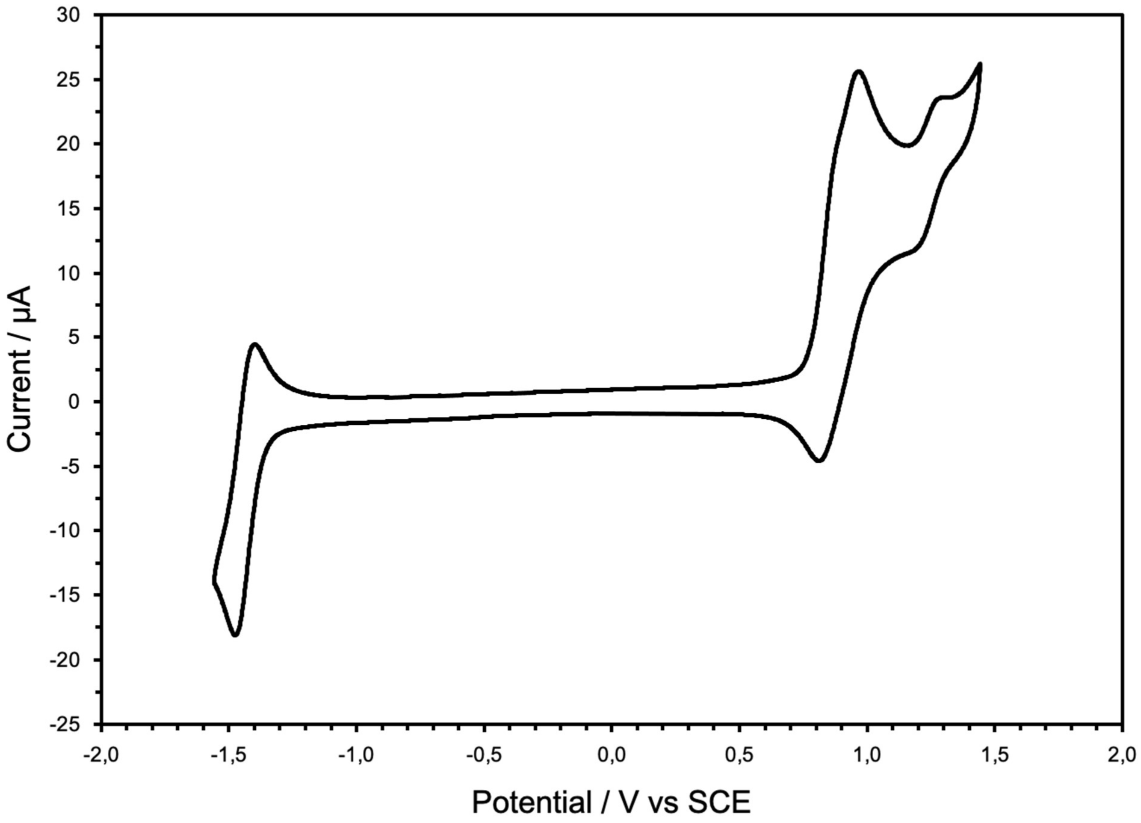

Figure S9: Cyclic Voltammogram of Cu(**bathocupSani**)(**DPEPhos**)BF_4 in MeCN (1 mM) using $TBAPF_6$ (100 mM) as supporting electrolyte at a scan rate of 50 $mV \cdot s^{-1}$.

NMR SPECTRA

^{1}H NMR (400 MHz, $CDCl_3$) of bathocupSani

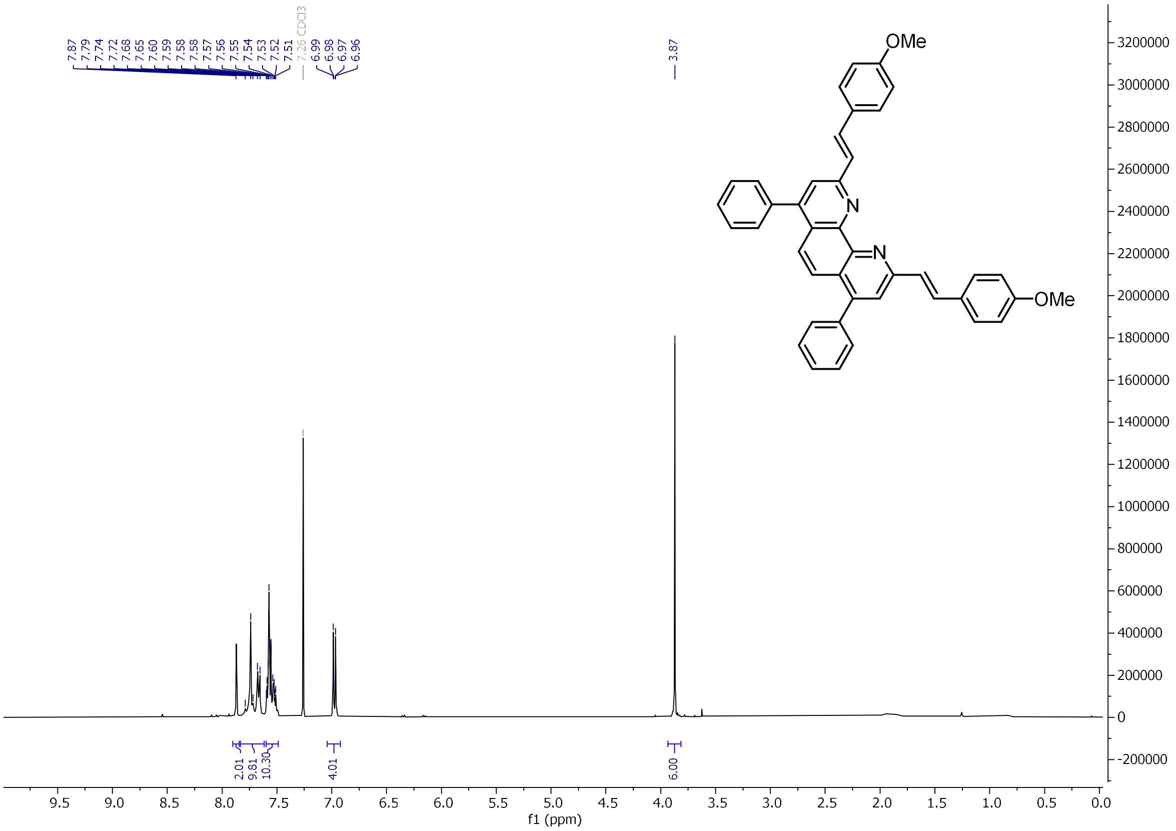

^{13}C NMR (101 MHz, CDCl$_3$) of bathocupSani

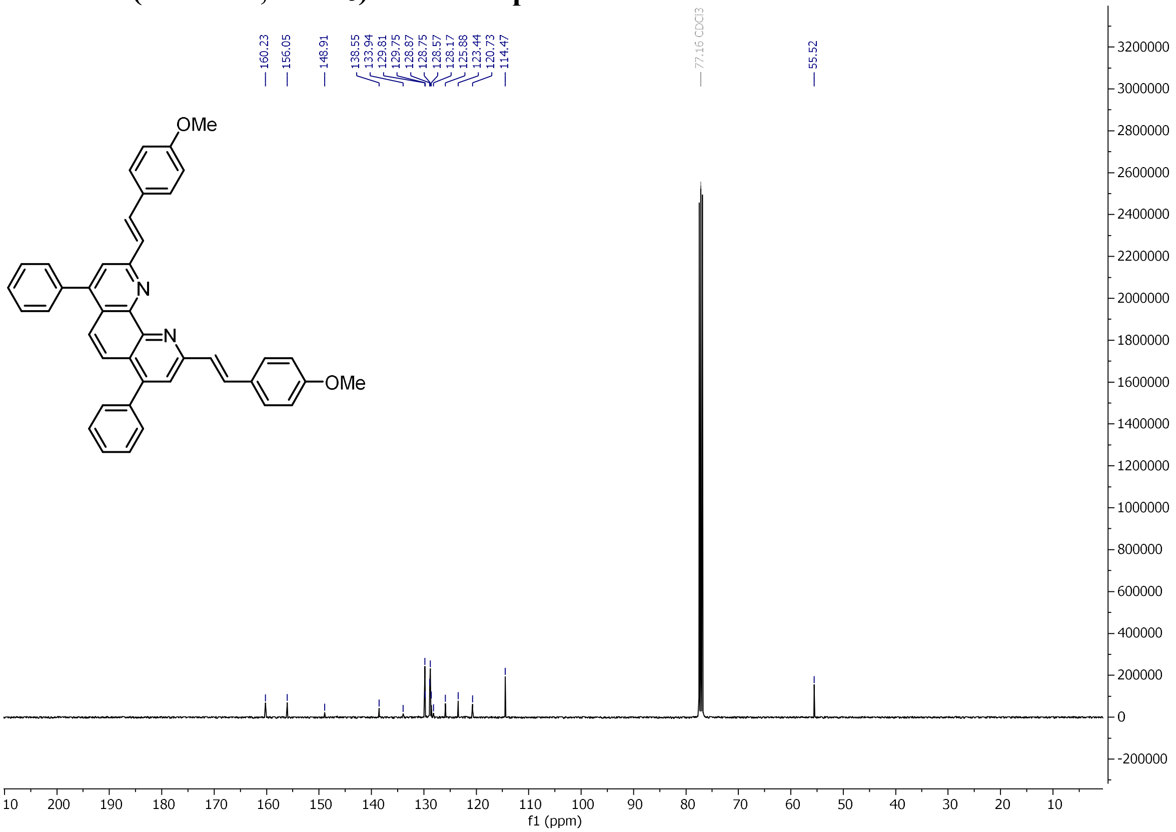

^{1}H NMR (400 MHz, Acetone-*d6*) of Cu(bathocupSani)$_2$BF$_4$

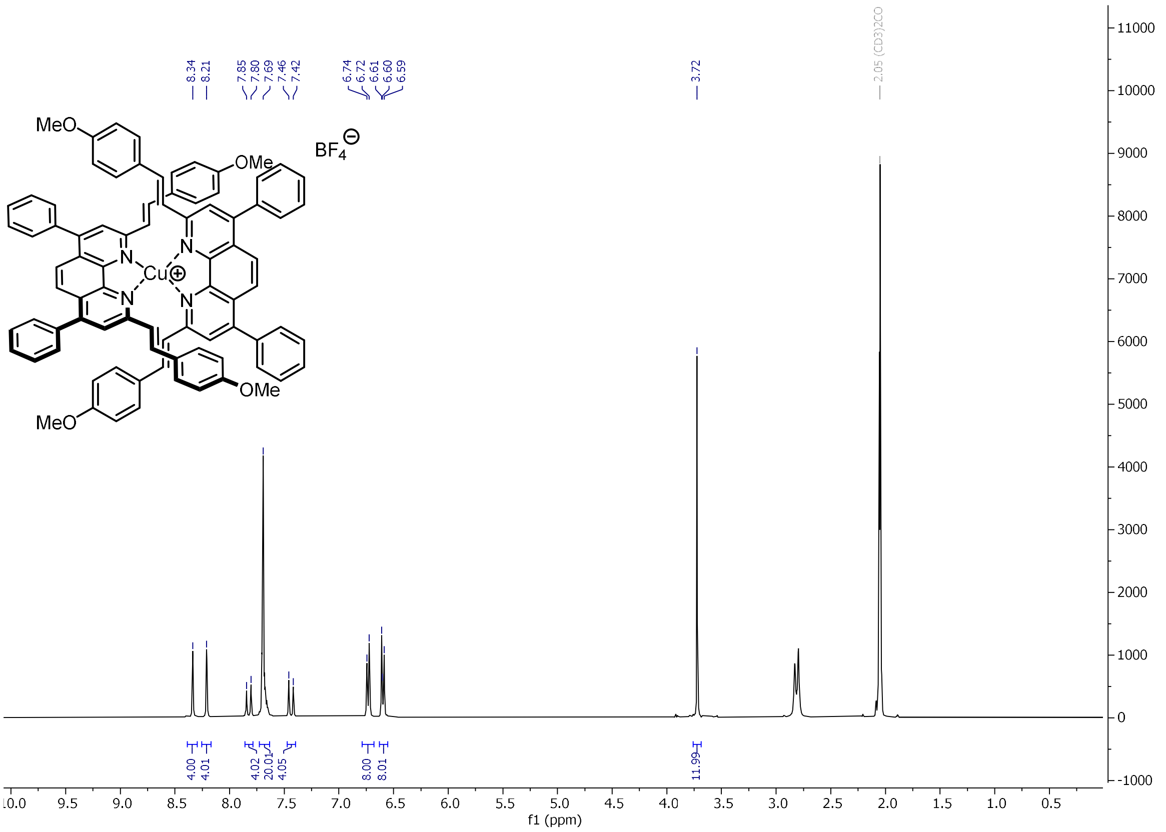

^{13}C NMR (101 MHz, Acetone-*d6*) of Cu(bathocupSani)$_2$BF$_4$

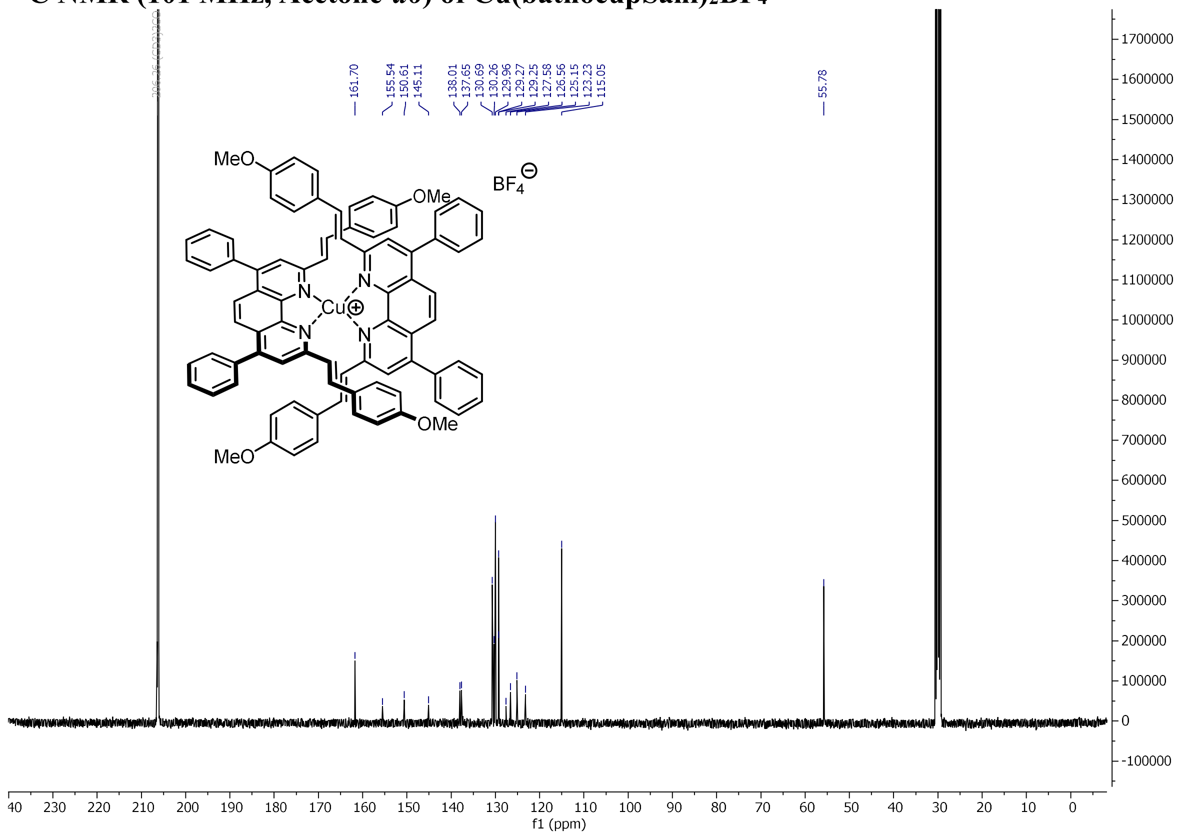

^{1}H NMR (400 MHz, Acetone-*d6*) of Cu(bathocupSani)(DPEPhos)BF_4

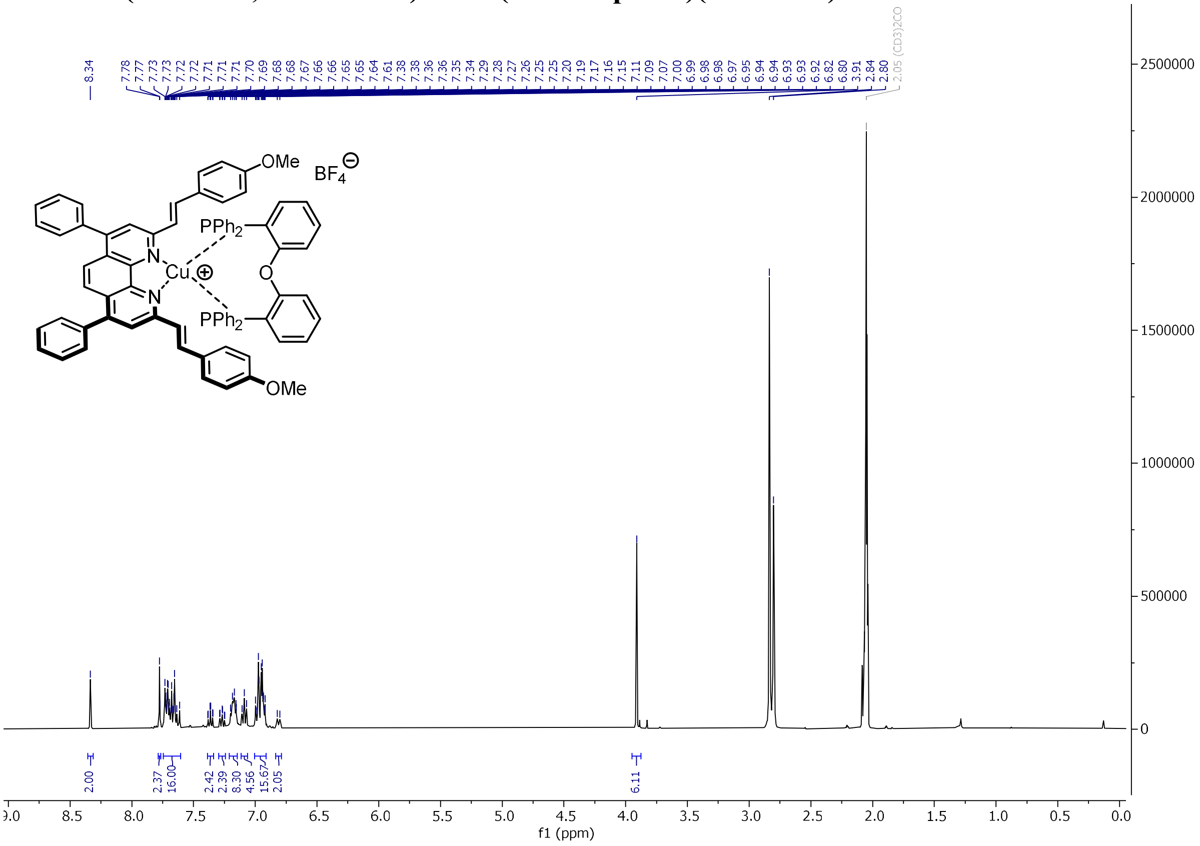

^{13}C NMR (101 MHz, Acetone-*d6*) of Cu(bathocupSani)(DPEPhos)BF_4

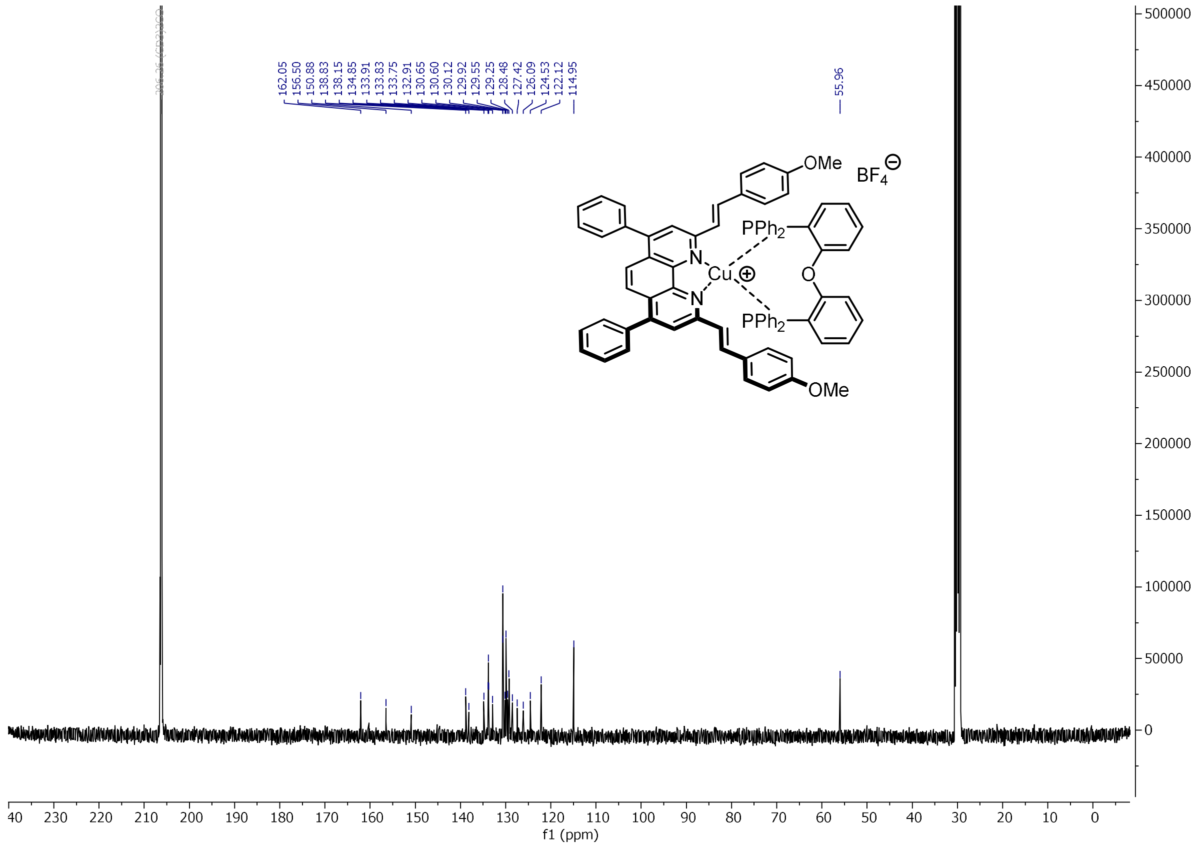

¹H RMN of photochemical reaction scope*

*Internal standard pics of 1,4-dimethoxybenzene at 6.80 ppm (s) and 3.73 ppm (s).

¹H NMR (400 MHz, $CDCl_3$) of N-benzylidenebenzylamine

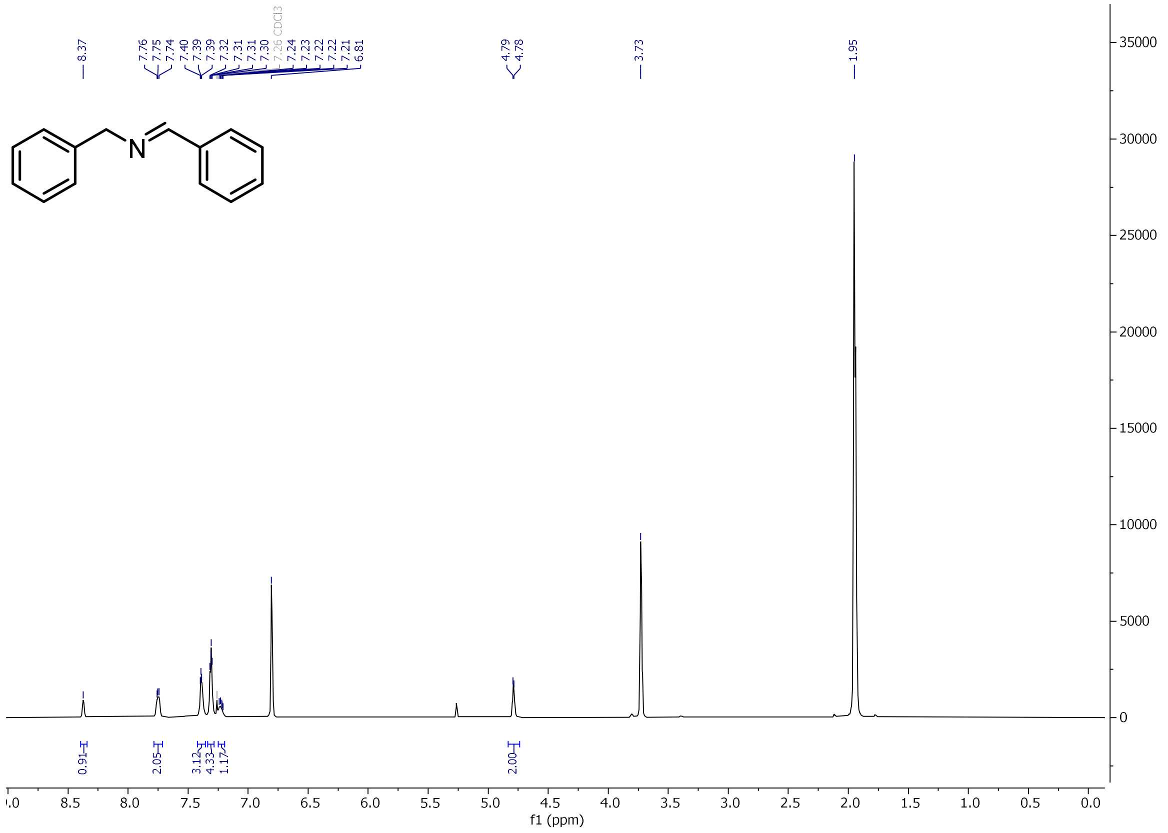

^{1}H NMR (400 MHz, $CDCl_3$) of N-(4-methylbenzylidene)-4-methylbenzylamine

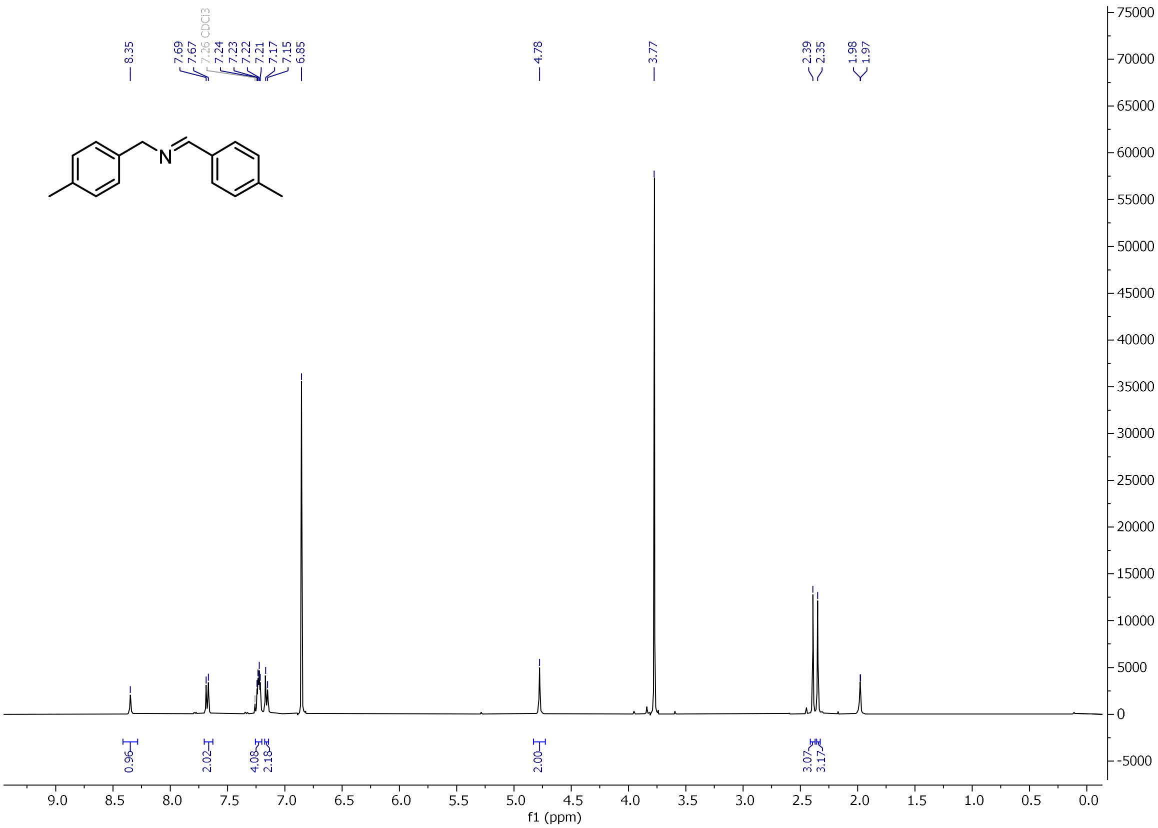

^{1}H NMR (400 MHz, $CDCl_3$) of N-(4-methoxybenzylidene)-4-methoxybenzylamine

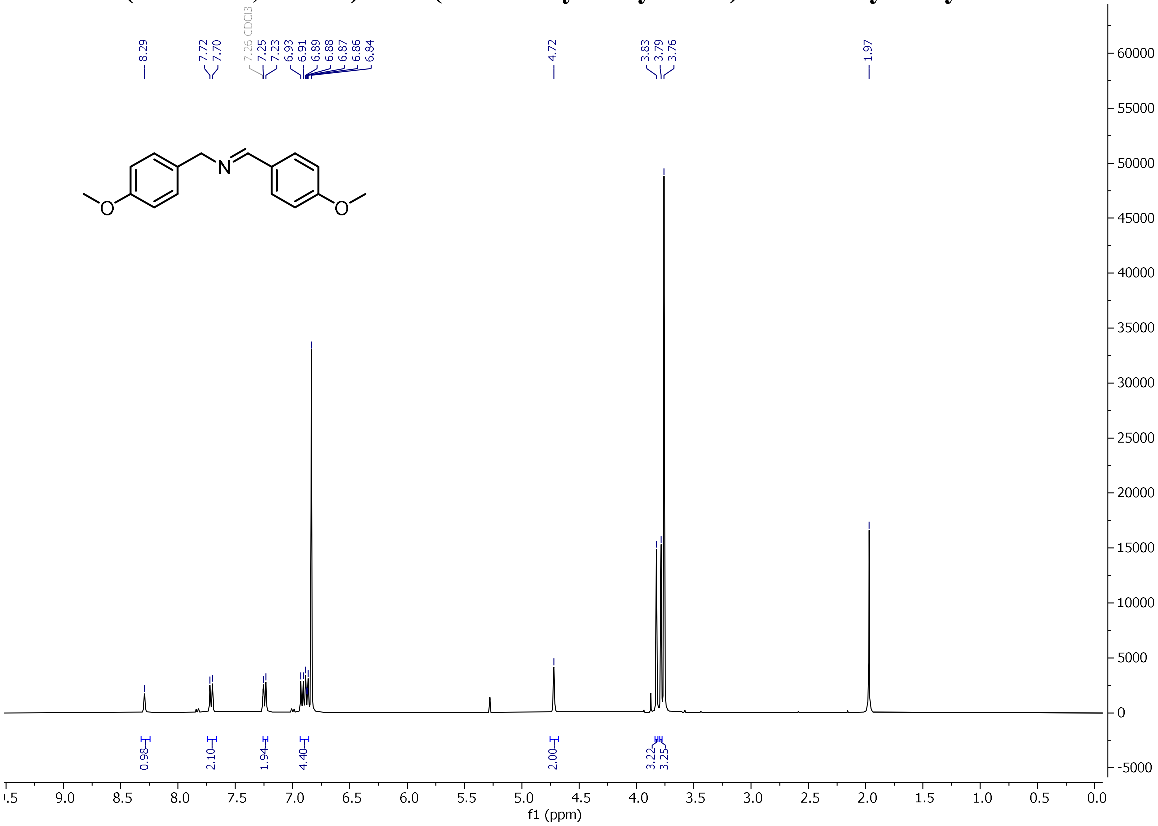

^{1}H NMR (400 MHz, $CDCl_3$) of N-(4-methoxycarbonylbenzyl)-N-(4-methoxycarbonylbenzylidene)amine

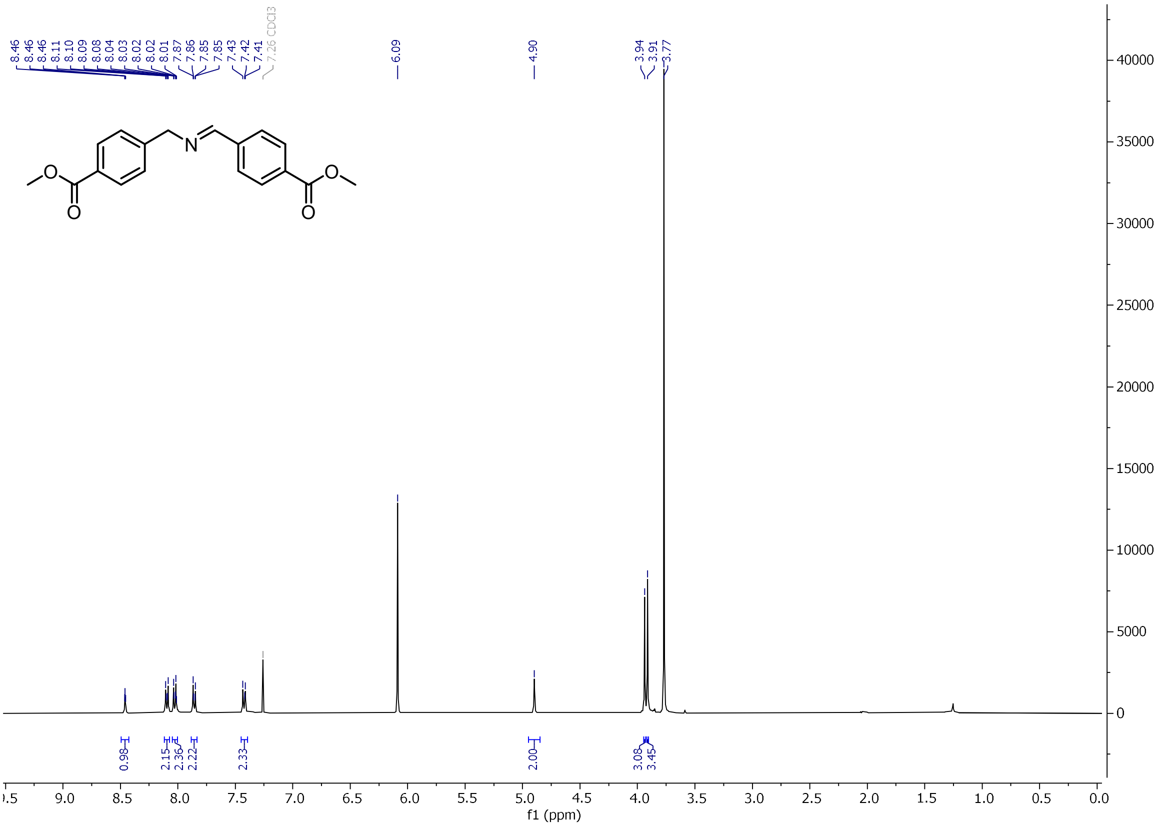

^{1}H NMR (400 MHz, $CDCl_3$) of Benzophenone N-benzhydrylimine

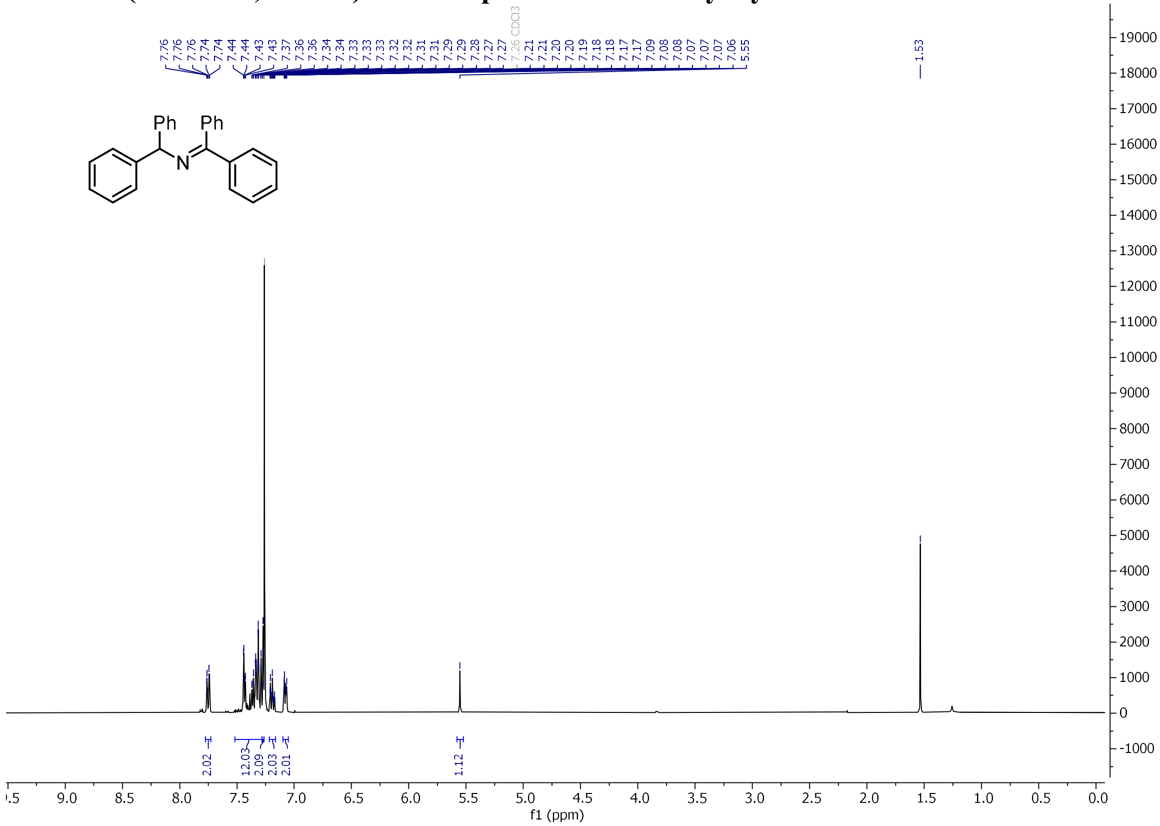

^{1}H NMR (400 MHz, $CDCl_3$) of

*Internal standard pics of 1,3,5-trimethoxybenzene at 6.09 ppm (s) and 3.77 ppm (s).

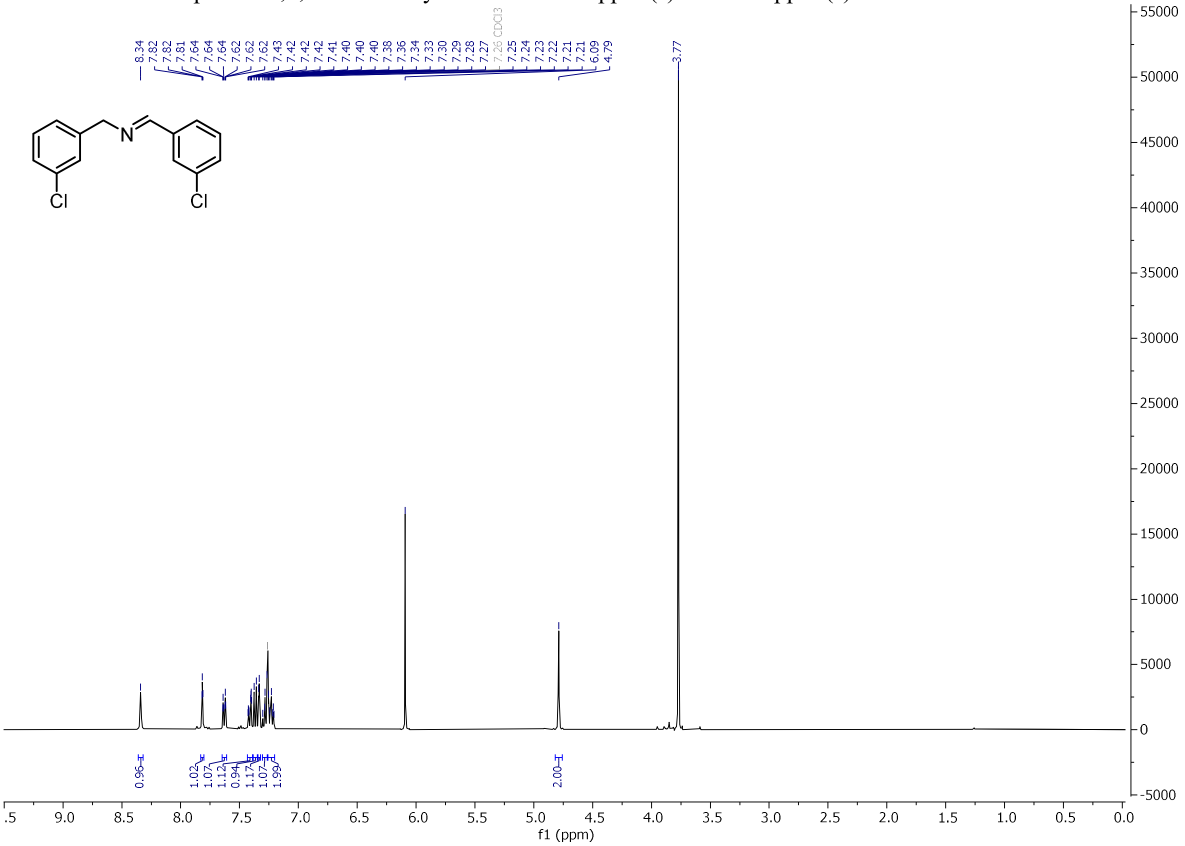

^{1}H NMR (400 MHz, CDCl₃) of N-(3-methoxybenzylidene)-3-methoxyphenylmethylamine

*Internal standard pics of 1,3,5-trimethoxybenzene at 6.09 ppm (s) and 3.77 ppm (s).

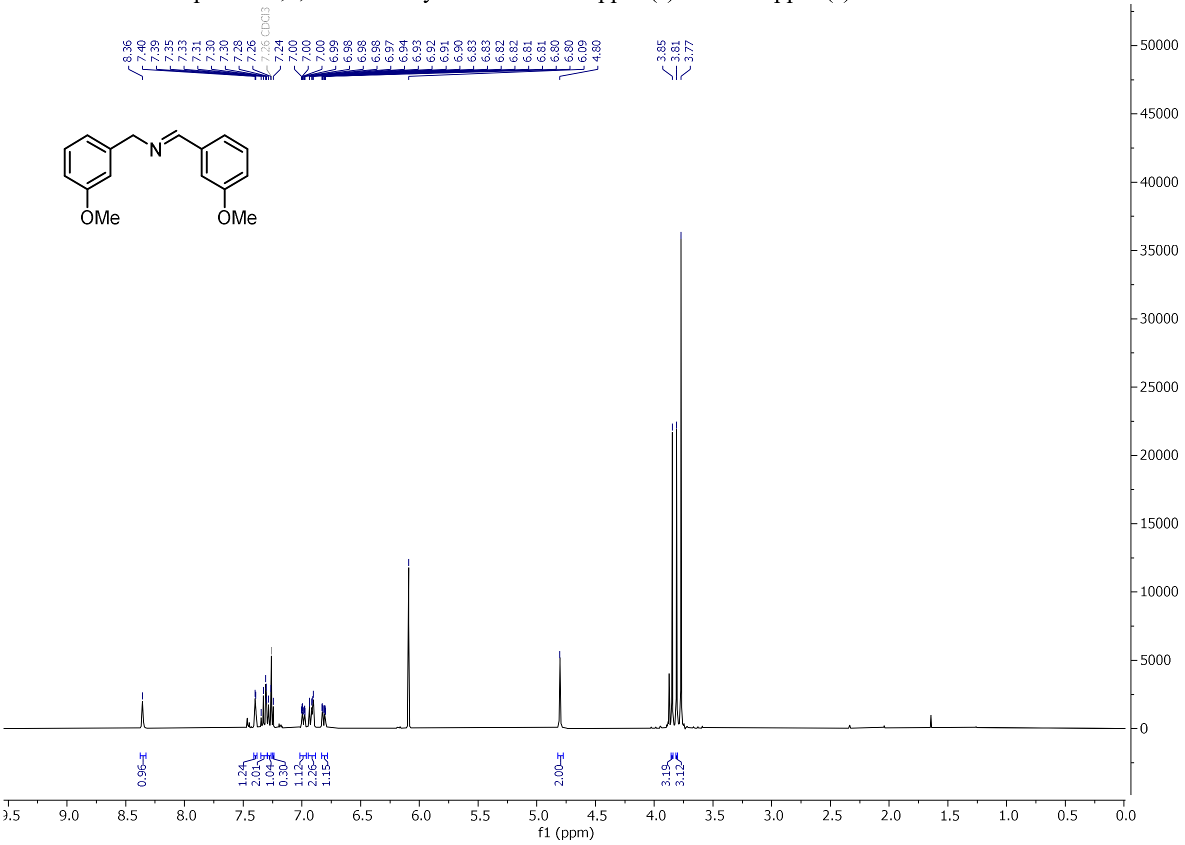

^{1}H NMR (400 MHz, $CDCl_3$) of N-(3,5-bis(trifluoromethyl)benzylidene)-1-(3,5 bis(trifluoromethyl)phenyl)methanamine

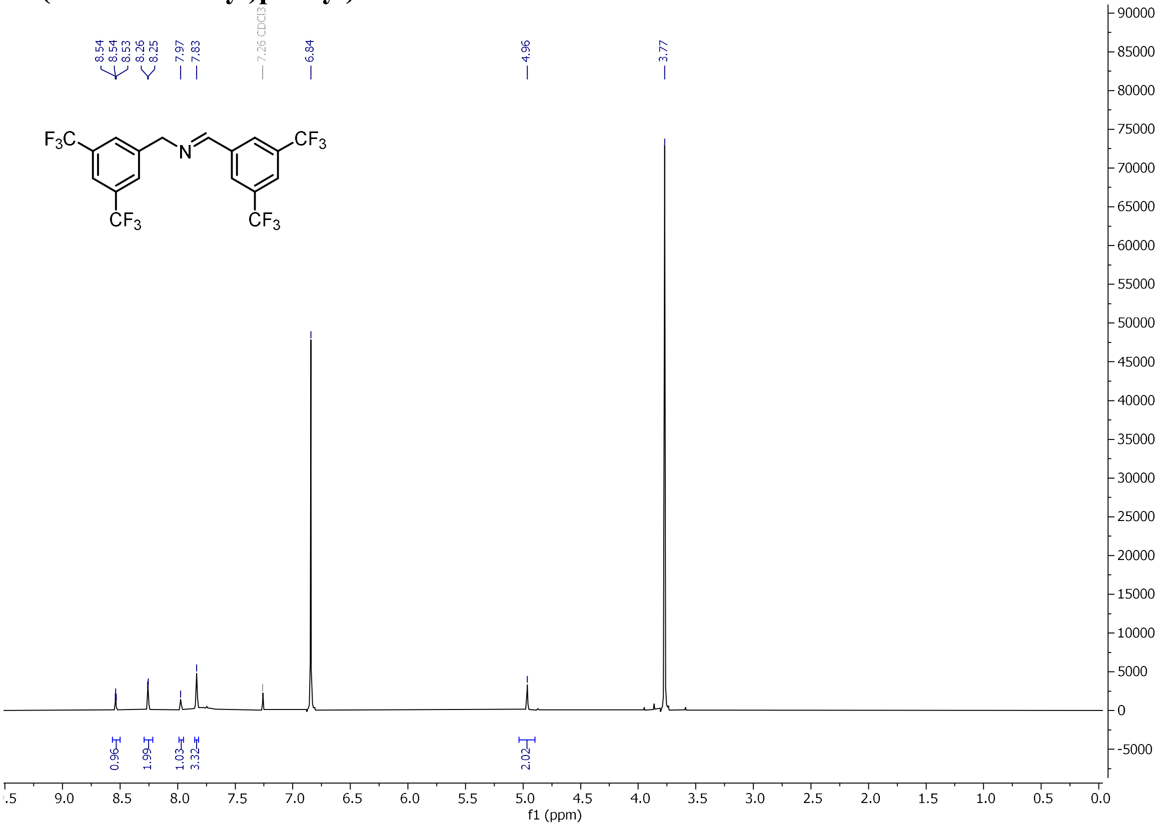

^{1}H NMR (400 MHz, $CDCl_3$) of 4-(((4-cyanobenzyl)imino)methyl)benzonitrile

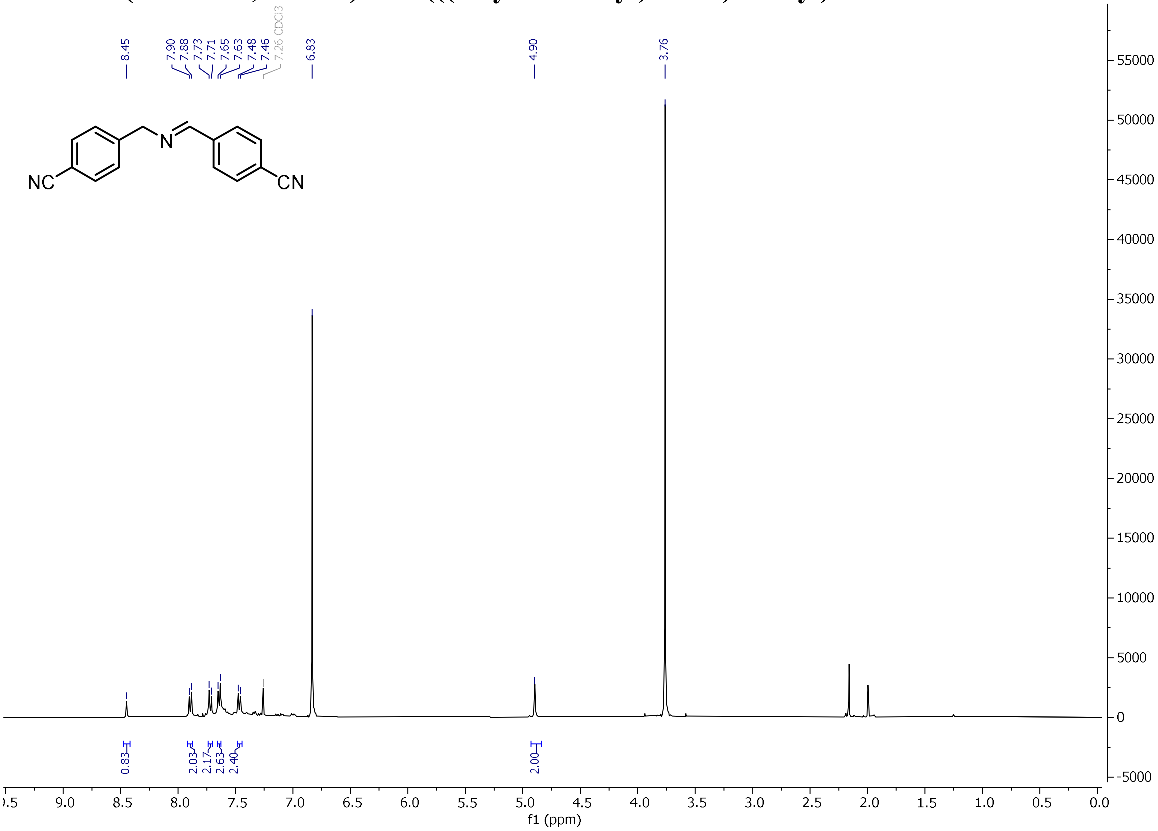

^{1}H NMR (400 MHz, CDCl$_3$) of Dibenzylamine

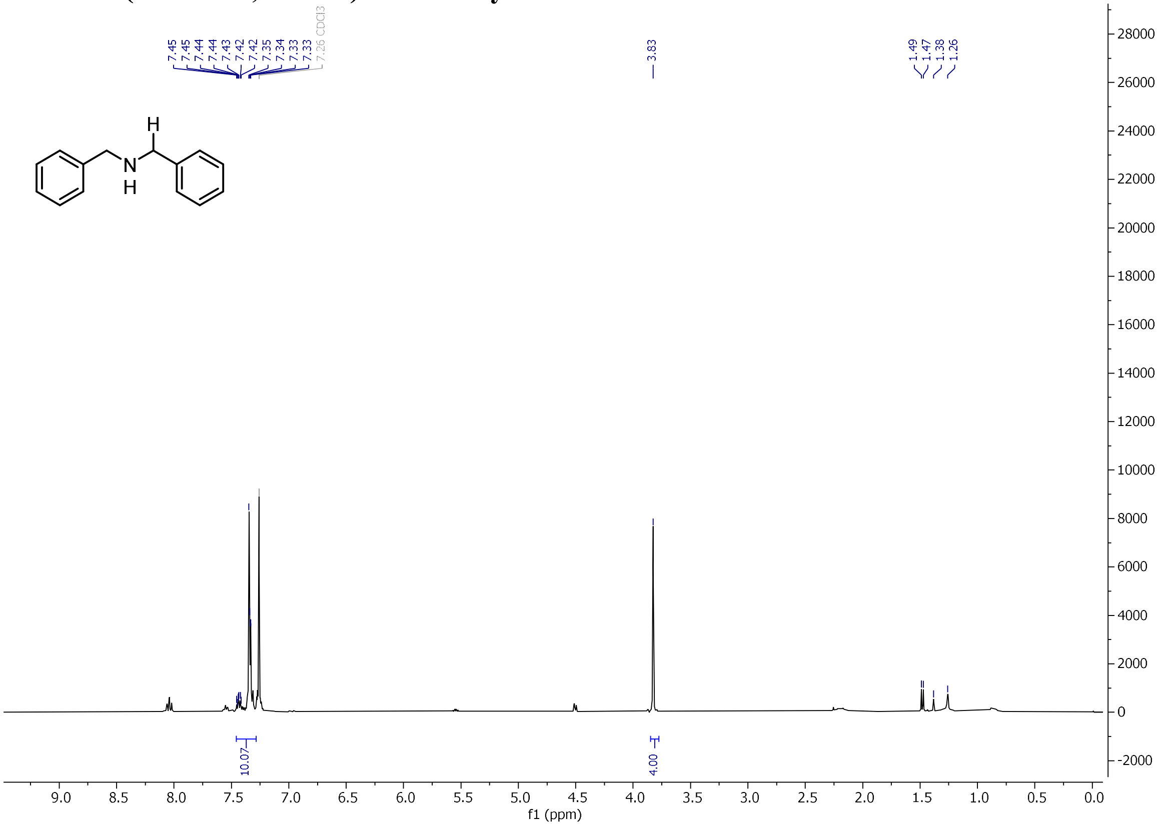

^{1}H NMR (400 MHz, CDCl$_3$) of Methyl phenyl sulfoxide

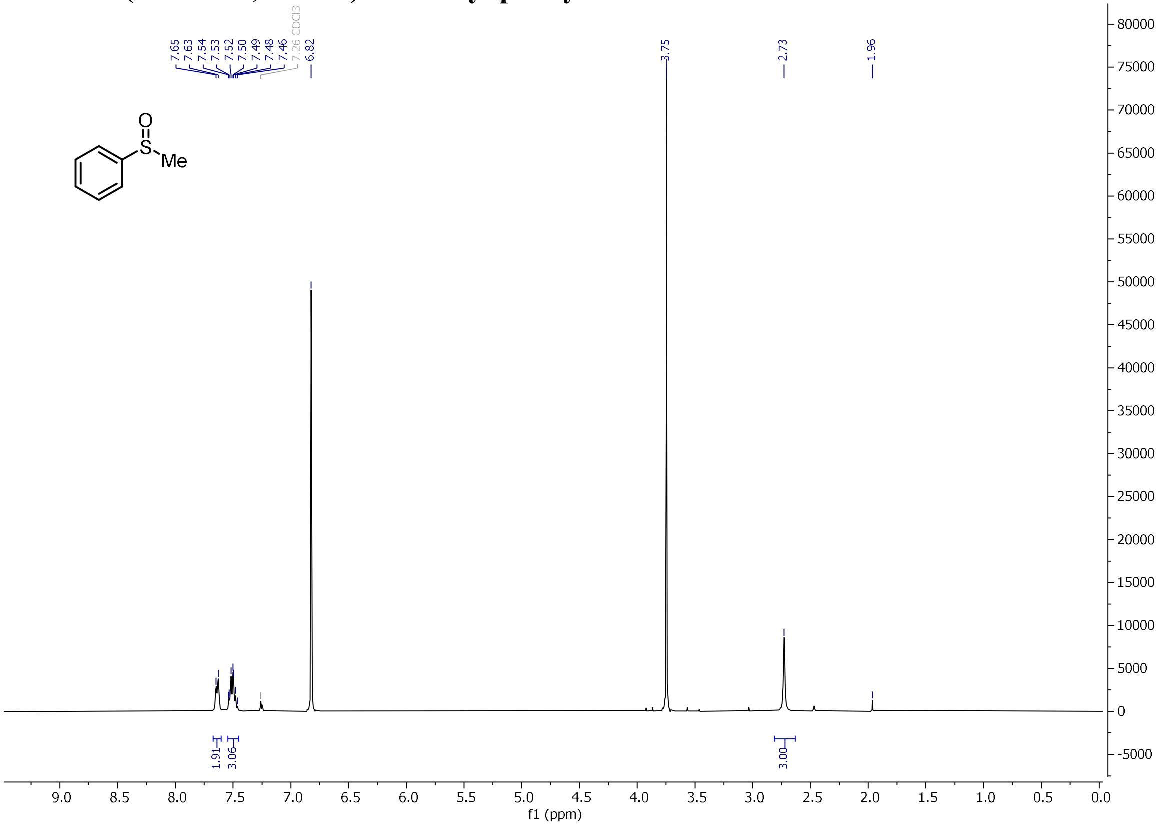

^{1}H NMR (400 MHz, $CDCl_3$) of Triphenylphosphine oxide

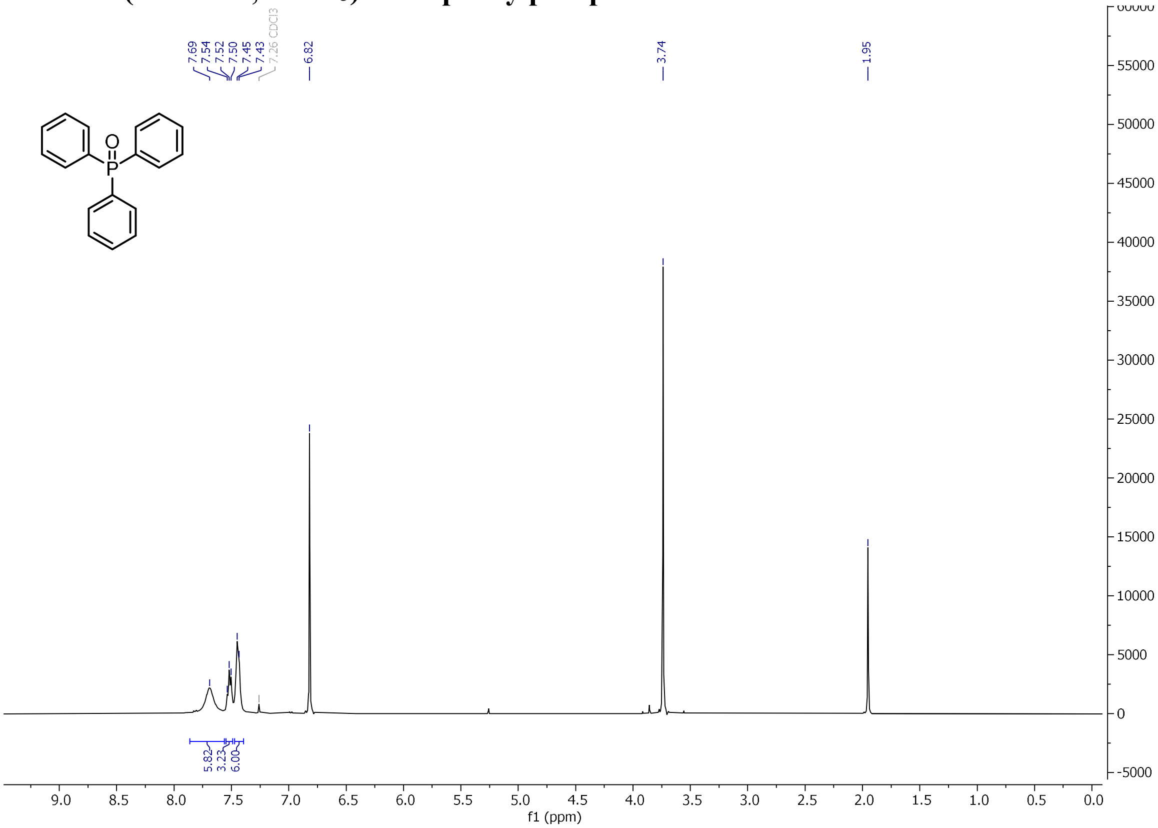

^1H NMR (400 MHz, $CDCl_3$) of 4-Phenylphenol

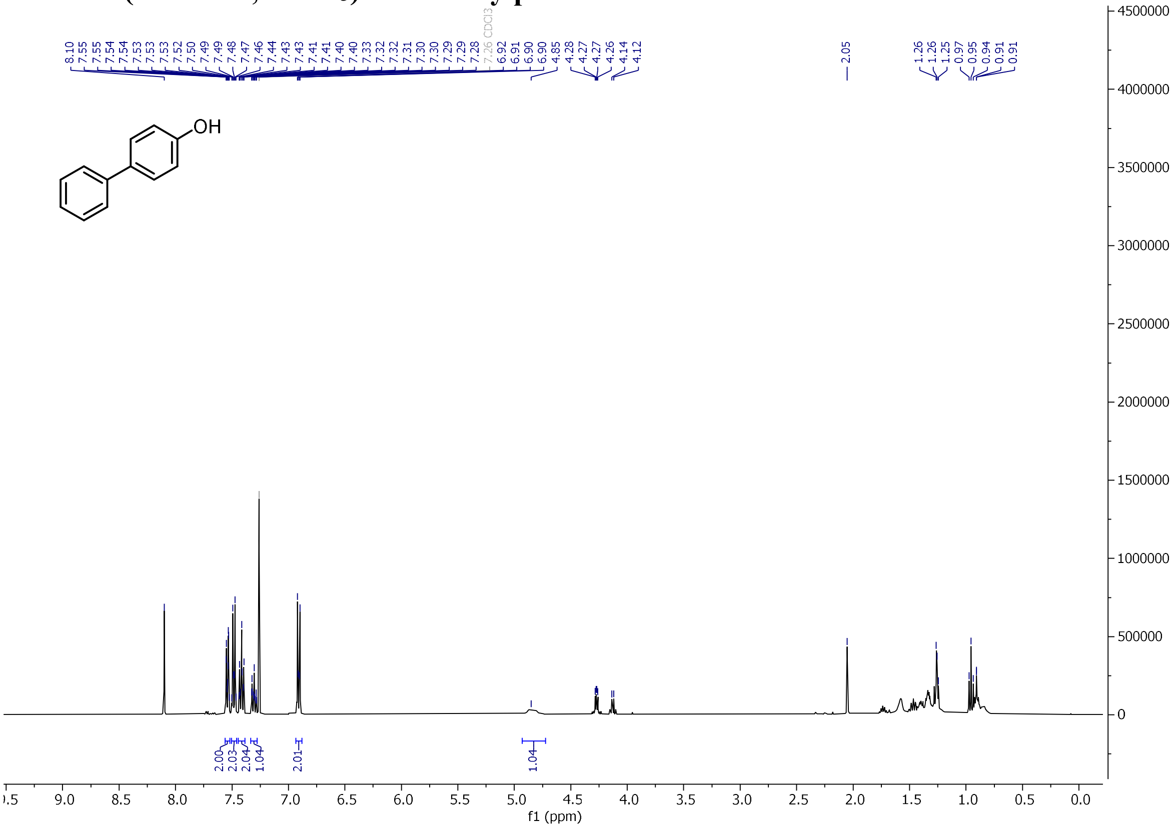

^1H NMR (400 MHz, $CDCl_3$) of 4-Methoxy-phenol

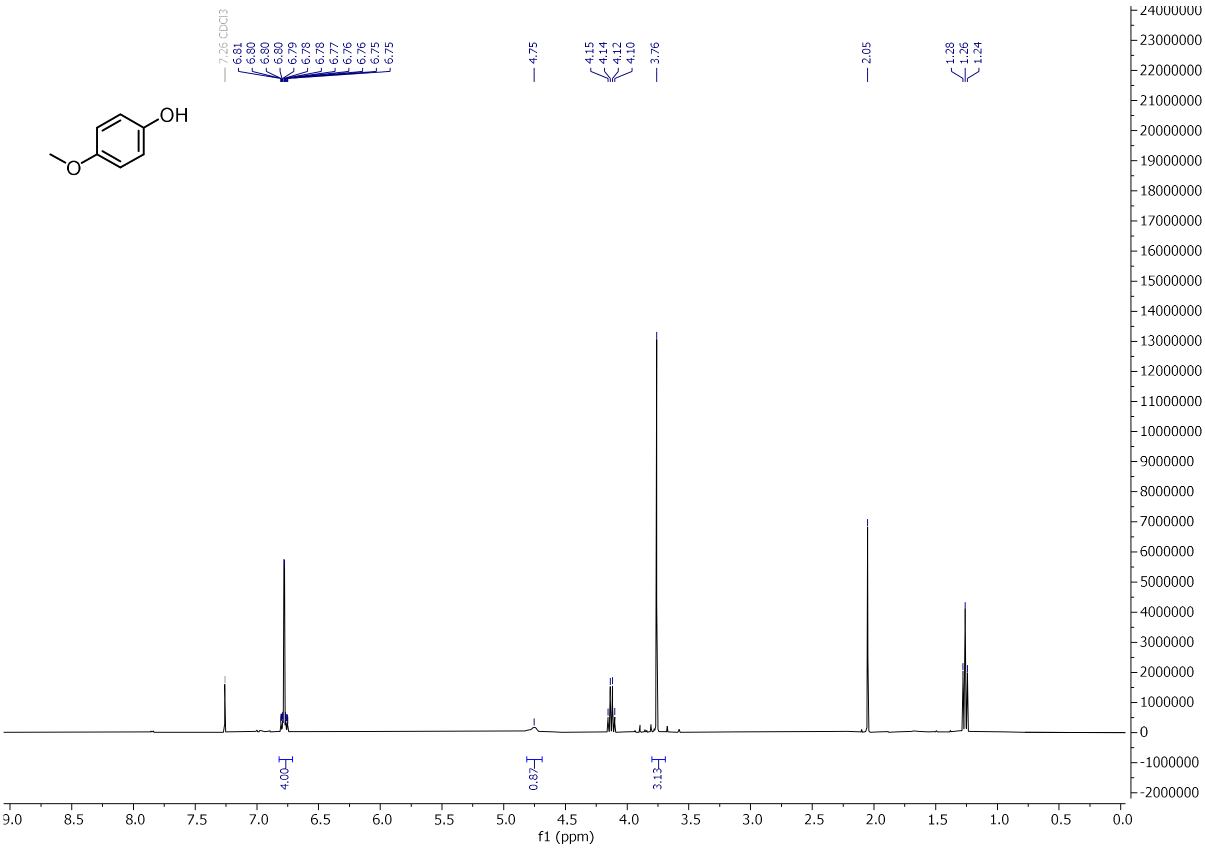

^{1}H NMR (400 MHz, $CDCl_3$) of bis[2-(diphenylphosphino)phenyl]ether oxide:

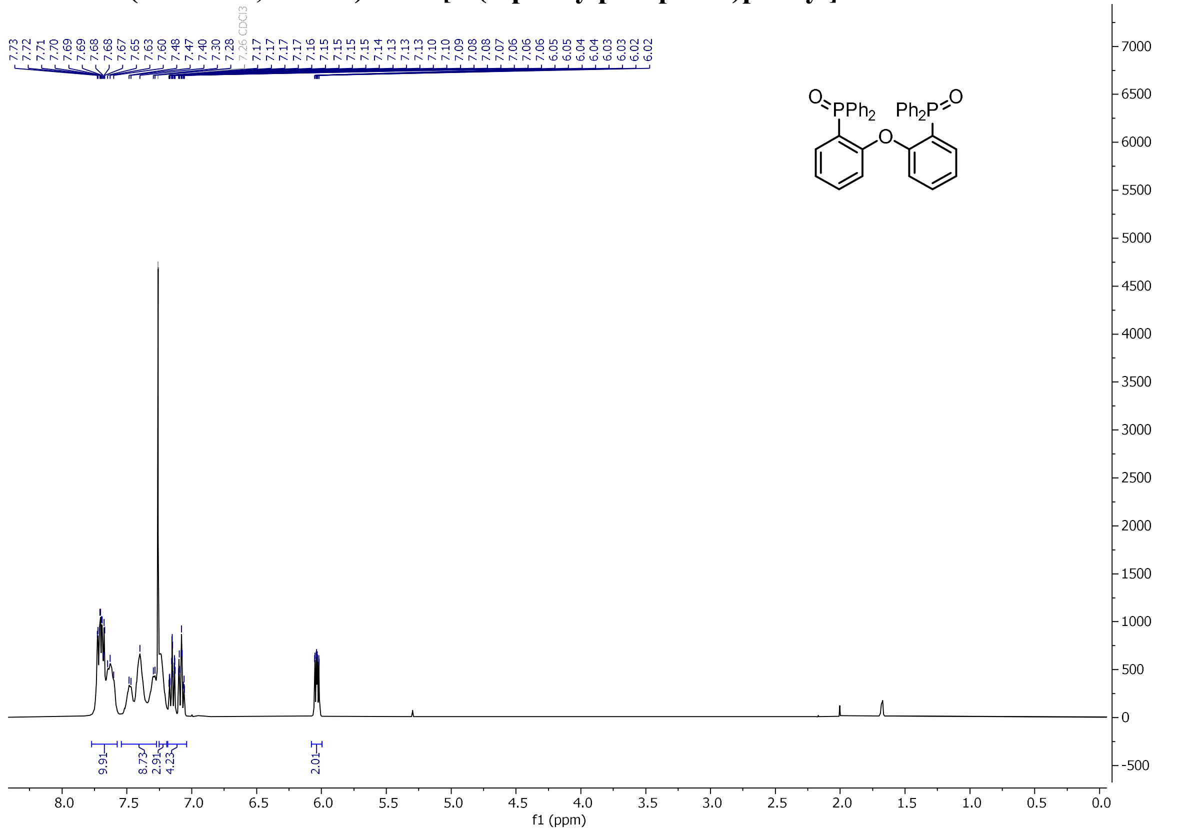

^{1}H NMR (400 MHz, $CDCl_3$) of (9,9-dimethyl-9H-xanthene-4,5-diyl) bis(diphenylphosphineoxide):

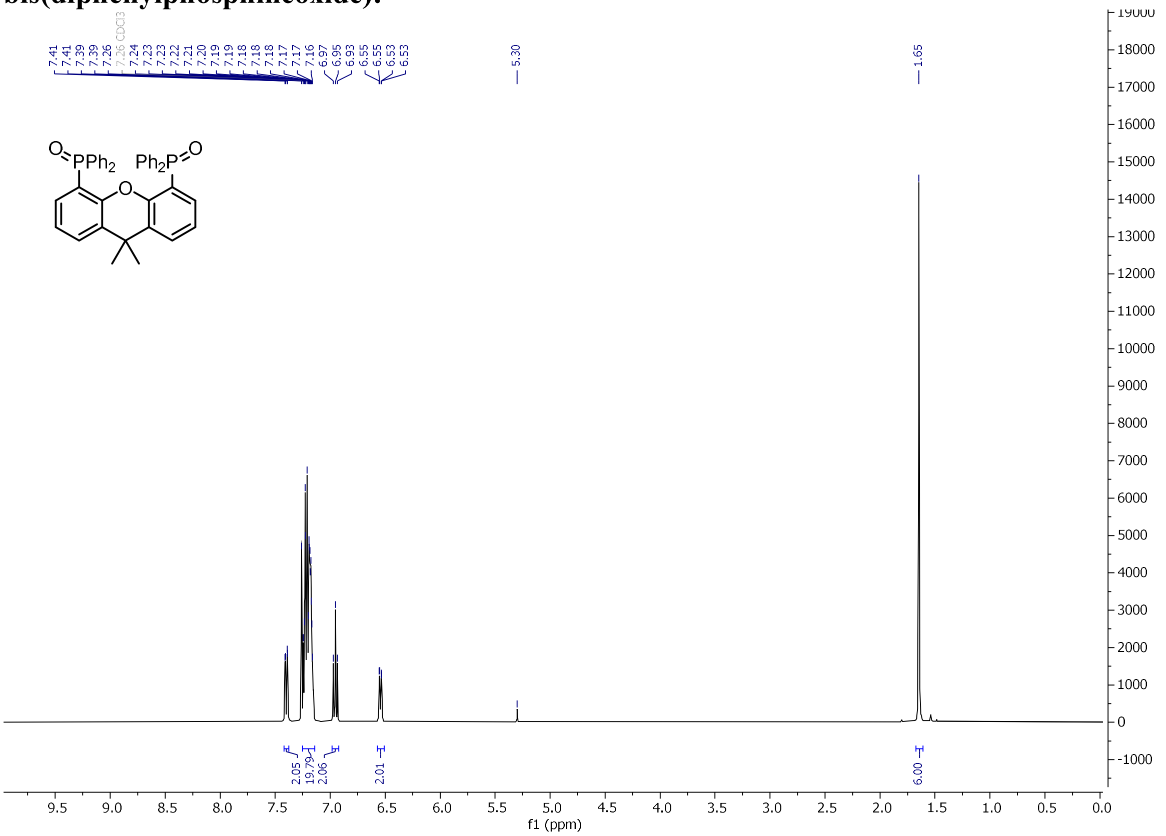

^1H NMR (400 MHz, CDCl$_3$) of 2-azido-1-phenylethan-1-one

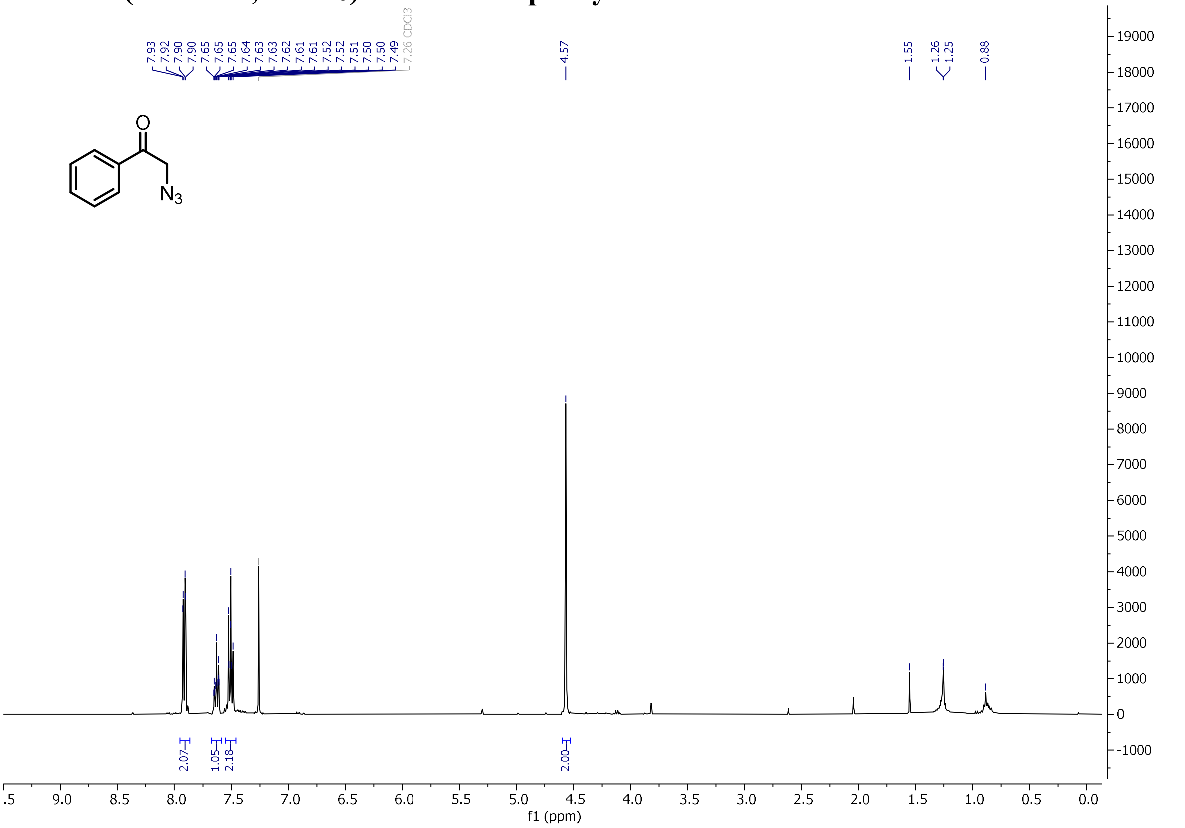

^{1}H NMR (400 MHz, $CDCl_3$) of (1,3,3,3-tetrabromopropyl)benzene

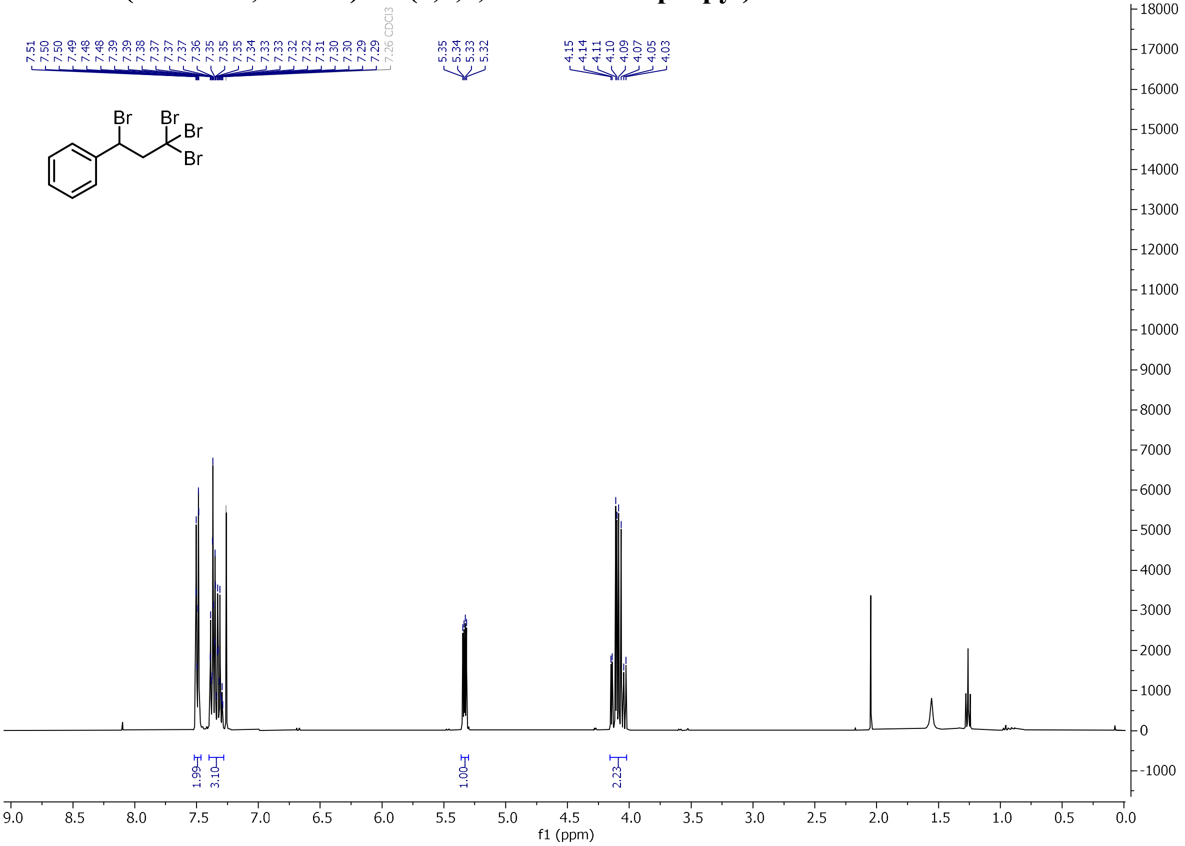

X-RAY CRYSTALOGRAPHY

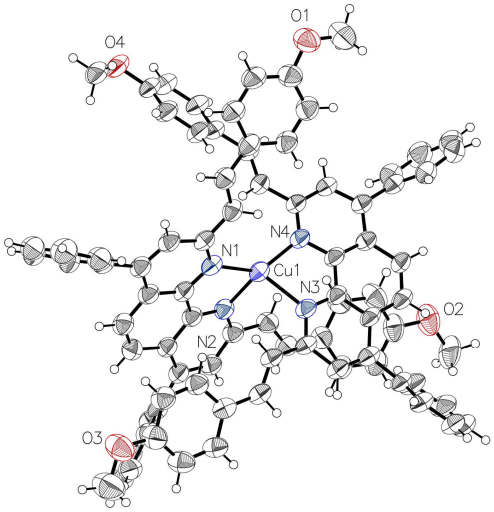

The data for Cu(**bathocupSani**)$_2$BF$_4$, crystallized from DCM/Et$_2$O, were collected from a shock-cooled single crystal at 150 K on a Bruker Venture Metaljet k-geometry diffractometer with a Metal Jet using a Helios MX Mirror Optics as monochromator and a Bruker CMOS Photon III detector. The diffractometer was equipped with an Oxford Cryostream 700 low temperature device and used Ga K_α radiation (λ = 1.34139 Å). All data were integrated with *SAINT* and a multi-scan absorption correction using *SADABS* was applied.[13-14] The structure was solved by intrinsic phasing methods with *XT* and refined by full-matrix least-squares methods against F^2 using *XL*.[15-16] All non-hydrogen atoms were refined with anisotropic displacement parameters. The hydrogen atoms were refined isotropically on calculated positions using a riding model with their U_{iso} values constrained to 1.5 times the U_{eq} of their pivot atoms for terminal sp^3 carbon atoms and 1.2 times for all other carbon atoms. Disordered water molecules were refined as single isolated oxygen atoms with adjusted occupancy factors. This report and the CIF file were generated using FinalCif.[17]

[13] Bruker, *SAINT*, *(2020)*, Bruker AXS Inc., Madison, Wisconsin, USA.

[14] Krause, L.; Herbst-Irmer, R.; Sheldrick, G. M.; Stalke, D. *J. Appl. Cryst.* **2015**, *48*, 3.

[15] Sheldrick, G. M. *Acta Cryst.* **2015**, *C71*, 3.

[16] Dolomanov, O. V.; Bourhis, L. J.; Gildea, R. J.; Howard, J. A. K.; Puschmann, H. *J. Appl. Cryst.* **2009**, 42, 339.

[17] D. Kratzert, *FinalCif*, *V84*, https://www.xs3.uni-freiburg.de/research/finalcif.

Table 1. Crystal data and structure refinement for Cu(**bathocupSani**)$_2$BF$_4$

Empirical formula	$C_{84}H_{64}BCuF_4N_4O_{5.75}$
Formula weight	1371.74
Temperature [K]	150
Crystal system	monoclinic
Space group (number)	$P2_1/n$ (14)
a [Å]	19.375(2)
b [Å]	13.1376(14)
c [Å]	26.461(3)
α [°]	90
β [°]	97.144(6)
γ [°]	90
Volume [Å^3]	6683.1(13)
Z	4
ρ_{calc} [gcm^{-3}]	1.363
μ [mm^{-1}]	2.139
F(000)	2848
Crystal size [mm^3]	0.03×0.12×0.35
Crystal colour	clear light red
Crystal shape	plate
Radiation	Ga K_α (λ=1.34139 Å)
2θ range [°]	5.24 to 110.80 (0.81 Å)
Index ranges	$-23 \leq h \leq 23$<br>$-15 \leq k \leq 16$<br>$-32 \leq l \leq 32$
Reflections collected	98572
Independent reflections	12823<br>R_{int} = 0.1238<br>R_{sigma} = 0.0756
Completeness to θ = 53.594°	99.9 %
Data / Restraints / Parameters	12823 / 36 / 912
Goodness-of-fit on F^2	1.036
Final R indexes [$I\geq2\sigma(I)$]	R_1 = 0.0709<br>wR_2 = 0.1838
Final R indexes [all data]	R_1 = 0.1302<br>wR_2 = 0.2201
Largest peak/hole [eÅ^{-3}]	0.49/−0.48

Table 2. Atomic coordinates and U_{eq} [Å^2] for Cu(**bathocupSani**)$_2$BF$_4$

Atom	*x*	*y*	*z*	U_{eq}
Cu1	0.80606(3)	0.68333(4)	0.27952(2)	0.0563(2)
C1	0.95892(17)	0.7344(3)	0.27741(14)	0.0541(9)
N1	0.90030(15)	0.6881(2)	0.25606(12)	0.0557(8)
O1	1.05016(17)	0.8852(3)	0.56906(12)	0.0862(9)
N2	0.78820(14)	0.5807(2)	0.21971(11)	0.0540(7)
C2	1.01989(18)	0.7311(3)	0.25375(15)	0.0600(10)
H2	1.060044	0.765870	0.269260	0.072
O2	0.39048(18)	0.5345(3)	0.34158(14)	0.0992(11)
O3	0.90598(19)	0.7795(3)	−0.02424(13)	0.1004(11)
N3	0.71854(14)	0.7753(2)	0.25979(11)	0.0537(7)
C3	1.02360(19)	0.6795(3)	0.20900(15)	0.0583(10)
N4	0.76471(15)	0.6738(2)	0.34513(12)	0.0537(8)
O4	1.16873(14)	0.3946(3)	0.45003(11)	0.0797(9)
C4	0.96160(18)	0.6335(3)	0.18530(14)	0.0569(9)
C5	0.95569(19)	0.5851(3)	0.13682(15)	0.0610(10)
H5	0.995368	0.582182	0.119085	0.073
C6	0.8956(2)	0.5431(3)	0.11525(15)	0.0604(10)
H6	0.893748	0.512808	0.082508	0.072
C7	0.83466(18)	0.5432(3)	0.14061(14)	0.0530(9)
C8	0.76958(18)	0.5021(3)	0.12006(14)	0.0543(9)
C9	0.71817(19)	0.4973(3)	0.15098(14)	0.0580(10)
H9	0.674567	0.468307	0.138175	0.070
C10	0.72813(18)	0.5341(3)	0.20120(14)	0.0551(9)
C11	0.83968(17)	0.5850(3)	0.18994(14)	0.0526(9)
C12	0.90250(18)	0.6376(3)	0.21084(14)	0.0545(9)
C13	1.0913(2)	0.6698(4)	0.18962(15)	0.0653(11)
C14	1.1350(2)	0.7512(4)	0.18710(17)	0.0798(13)
H14	1.119243	0.817583	0.194263	0.096
C15	1.2017(3)	0.7385(6)	0.1743(2)	0.0971(17)
H15	1.231261	0.795774	0.172835	0.116
C16	1.2245(3)	0.6451(7)	0.1639(2)	0.104(2)
H16	1.270504	0.637358	0.155619	0.125
C17	1.1832(3)	0.5608(6)	0.16501(19)	0.0983(17)
H17	1.200011	0.495468	0.157069	0.118
C18	1.1162(2)	0.5725(4)	0.17798(17)	0.0815(14)
H18	1.087016	0.514631	0.179016	0.098
C19	0.75462(18)	0.4678(3)	0.06611(14)	0.0564(9)
C20	0.7706(2)	0.5295(4)	0.02653(16)	0.0695(11)
H20	0.793960	0.592416	0.033866	0.083
C21	0.7527(2)	0.4997(4)	−0.02358(17)	0.0788(13)
H21	0.762808	0.542851	−0.050496	0.095
C22	0.7204(2)	0.4082(5)	−0.03452(17)	0.0822(14)
H22	0.708299	0.388015	−0.068958	0.099
C23	0.7052(2)	0.3450(4)	0.00453(18)	0.0741(13)
H23	0.683518	0.281053	−0.002956	0.089
C24	0.72204(18)	0.3759(3)	0.05448(15)	0.0603(10)
H24	0.710959	0.333072	0.081264	0.072
C25	0.95797(19)	0.7788(3)	0.32780(15)	0.0574(9)

H25	0.914118	0.792574	0.338878	0.069
C26	1.0156(2)	0.8013(3)	0.35942(15)	0.0619(10)
H26	1.058312	0.796098	0.345498	0.074
C27	1.02036(19)	0.8327(3)	0.41258(16)	0.0618(10)
C28	1.0871(2)	0.8385(4)	0.44095(17)	0.0711(12)
H28	1.127278	0.828335	0.424321	0.085
C29	1.0945(2)	0.8586(4)	0.49243(17)	0.0750(12)
H29	1.139718	0.862305	0.510940	0.090
C30	1.0372(2)	0.8734(4)	0.51716(17)	0.0714(11)
C31	0.9712(2)	0.8739(4)	0.48978(17)	0.0720(11)
H31	0.931540	0.888629	0.506303	0.086
C32	0.9639(2)	0.8528(3)	0.43819(16)	0.0654(11)
H32	0.918570	0.852043	0.419748	0.079
C33	0.9914(3)	0.8861(5)	0.5970(2)	0.0999(17)
H33A	0.963520	0.947241	0.588151	0.150
H33B	1.007531	0.886151	0.633575	0.150
H33C	0.962923	0.825432	0.588299	0.150
C34	0.67527(19)	0.5297(3)	0.23522(15)	0.0585(10)
H34	0.689699	0.542580	0.270275	0.070
C35	0.60805(19)	0.5093(3)	0.22175(15)	0.0594(10)
H35	0.595006	0.492791	0.186916	0.071
C36	0.5528(2)	0.5090(3)	0.25353(15)	0.0595(10)
C37	0.4841(2)	0.5050(3)	0.23108(17)	0.0649(10)
H37	0.475189	0.497068	0.195175	0.078
C38	0.4286(2)	0.5119(3)	0.25849(18)	0.0716(12)
H38	0.382372	0.507922	0.241738	0.086
C39	0.4406(2)	0.5247(4)	0.31045(19)	0.0747(12)
C40	0.5088(3)	0.5267(4)	0.33456(18)	0.0826(14)
H40	0.517163	0.533717	0.370531	0.099
C41	0.5642(2)	0.5185(4)	0.30701(17)	0.0726(12)
H41	0.610309	0.519300	0.324044	0.087
C42	0.3191(3)	0.5413(5)	0.3166(2)	0.113(2)
H42A	0.315347	0.597633	0.292131	0.169
H42B	0.287597	0.553515	0.342249	0.169
H42C	0.306346	0.477457	0.298722	0.169
C43	0.69465(18)	0.8147(3)	0.21394(15)	0.0553(10)
C44	0.62481(18)	0.8484(3)	0.20362(15)	0.0577(10)
H44	0.608715	0.875487	0.170991	0.069
C45	0.58066(19)	0.8428(3)	0.23924(15)	0.0586(10)
C46	0.60606(18)	0.8062(3)	0.28910(15)	0.0543(9)
C47	0.56723(19)	0.8044(3)	0.33088(15)	0.0591(10)
H47	0.522117	0.833905	0.327172	0.071
C48	0.59228(18)	0.7620(3)	0.37592(15)	0.0584(10)
H48	0.565251	0.763844	0.403497	0.070
C49	0.65917(18)	0.7144(3)	0.38248(14)	0.0559(9)
C50	0.68484(19)	0.6584(3)	0.42691(14)	0.0574(10)
C51	0.74814(18)	0.6115(3)	0.42793(15)	0.0587(10)
H51	0.765367	0.571769	0.456740	0.070
C52	0.78830(18)	0.6208(3)	0.38738(14)	0.0548(9)
C53	0.70064(17)	0.7188(3)	0.34287(14)	0.0536(9)

C54	0.67546(18)	0.7696(3)	0.29585(14)	0.0546(9)
C55	0.50538(18)	0.8704(3)	0.22819(14)	0.0575(10)
C56	0.4846(2)	0.9659(3)	0.21060(15)	0.0634(10)
H56	0.518323	1.014379	0.203301	0.076
C57	0.4145(2)	0.9911(4)	0.20356(16)	0.0743(12)
H57	0.400253	1.057093	0.191785	0.089
C58	0.3659(2)	0.9209(4)	0.21360(17)	0.0795(14)
H58	0.318069	0.939058	0.209209	0.095
C59	0.3852(2)	0.8242(4)	0.23001(18)	0.0797(14)
H59	0.351099	0.775367	0.236257	0.096
C60	0.4551(2)	0.7995(4)	0.23720(18)	0.0733(12)
H60	0.468999	0.733059	0.248460	0.088
C61	0.64546(19)	0.6516(4)	0.47143(15)	0.0619(10)
C62	0.6225(2)	0.5575(4)	0.48648(17)	0.0756(12)
H62	0.629824	0.498680	0.466910	0.091
C63	0.5892(3)	0.5481(5)	0.5296(2)	0.0885(15)
H63	0.572379	0.483767	0.539112	0.106
C64	0.5807(2)	0.6334(5)	0.55863(19)	0.0872(15)
H64	0.559633	0.627025	0.589031	0.105
C65	0.6026(2)	0.7287(5)	0.54393(18)	0.0822(14)
H65	0.596092	0.787059	0.564002	0.099
C66	0.6342(2)	0.7380(4)	0.49975(16)	0.0707(12)
H66	0.647973	0.803030	0.488933	0.085
C67	0.74292(19)	0.8156(3)	0.17657(15)	0.0574(10)
H67	0.791075	0.814456	0.189002	0.069
C68	0.7261(2)	0.8180(3)	0.12630(16)	0.0621(10)
H68	0.677926	0.824110	0.114512	0.075
C69	0.7725(2)	0.8125(3)	0.08712(16)	0.0610(10)
C70	0.7458(2)	0.8098(3)	0.03562(16)	0.0676(11)
H70	0.696989	0.816016	0.026670	0.081
C71	0.7869(2)	0.7987(4)	−0.00261(17)	0.0754(13)
H71	0.766577	0.796153	−0.037147	0.090
C72	0.8584(2)	0.7911(4)	0.00951(17)	0.0757(12)
C73	0.8865(2)	0.7954(4)	0.06065(17)	0.0733(12)
H73	0.935496	0.790971	0.069371	0.088
C74	0.8450(2)	0.8057(3)	0.09829(17)	0.0656(11)
H74	0.865600	0.808281	0.132775	0.079
C75	0.8805(3)	0.7708(7)	−0.0765(2)	0.131(2)
H75A	0.919246	0.756923	−0.096003	0.196
H75B	0.857702	0.834487	−0.088362	0.196
H75C	0.846859	0.714813	−0.081270	0.196
C76	0.85850(18)	0.5808(3)	0.38995(14)	0.0565(9)
H76	0.885024	0.600878	0.363764	0.068
C77	0.88927(18)	0.5183(3)	0.42576(14)	0.0560(9)
H77	0.861521	0.491678	0.449846	0.067
C78	0.96230(18)	0.4878(3)	0.43069(14)	0.0564(10)
C79	0.98392(19)	0.3978(3)	0.45546(15)	0.0612(10)
H79	0.950837	0.355051	0.468629	0.073
C80	1.0535(2)	0.3701(3)	0.46102(16)	0.0683(11)
H80	1.067552	0.308064	0.477687	0.082

C81	1.10170(19)	0.4304(4)	0.44294(15)	0.0639(11)
C82	1.0821(2)	0.5212(4)	0.41973(16)	0.0693(11)
H82	1.115895	0.564656	0.408023	0.083
C83	1.0129(2)	0.5486(3)	0.41354(15)	0.0651(11)
H83	0.999506	0.611090	0.397048	0.078
C84	1.2189(2)	0.4455(5)	0.42506(18)	0.0913(16)
H84A	1.202690	0.449721	0.388532	0.137
H84B	1.262915	0.407933	0.430248	0.137
H84C	1.226007	0.514338	0.439044	0.137
B1	0.2145(2)	0.7909(5)	0.3368(2)	0.0732(14)
F1	0.16655(17)	0.8494(4)	0.31060(17)	0.1529(17)
F2	0.27294(17)	0.7801(3)	0.31424(14)	0.1220(11)
F3	0.23382(18)	0.8355(3)	0.38215(13)	0.1349(14)
F4	0.1847(2)	0.7002(3)	0.3455(2)	0.1506(16)
O1A	1.0796(17)	0.443(3)	0.0639(14)	0.0973(18)
O1B	1.0656(3)	0.4933(4)	0.0316(2)	0.0973(18)
O1C	1.0398(14)	0.4511(18)	0.0553(9)	0.133(3)
O1D	1.0236(6)	0.4578(10)	0.0139(4)	0.133(3)

U_{eq} is defined as 1/3 of the trace of the orthogonalized U_{ij} tensor.

Table 3. Anisotropic displacement parameters (Å2) for Cu(**bathocupSani**)$_2$BF$_4$.

The anisotropic displacement factor exponent takes the form:
$-2\pi^2[h^2(a^*)^2U_{11} + k^2(b^*)^2U_{22} + \ldots + 2hka^*b^*U_{12}]$

**Atom**	U_{11}	U_{22}	U_{33}	U_{23}	U_{13}	U_{12}
Cu1	0.0391(3)	0.0697(4)	0.0590(4)	−0.0020(3)	0.0010(2)	0.0029(3)
C1	0.0371(18)	0.059(2)	0.064(2)	0.0032(19)	−0.0011(16)	−0.0003(17)
N1	0.0426(16)	0.0592(19)	0.063(2)	0.0026(15)	−0.0023(14)	0.0049(14)
O1	0.080(2)	0.106(3)	0.071(2)	−0.0112(17)	0.0043(16)	−0.0142(18)
N2	0.0406(15)	0.064(2)	0.0574(18)	0.0022(15)	0.0054(13)	0.0014(14)
C2	0.0376(19)	0.073(3)	0.068(3)	0.006(2)	0.0015(17)	−0.0017(18)
O2	0.082(2)	0.121(3)	0.104(2)	−0.004(2)	0.050(2)	−0.009(2)
O3	0.095(3)	0.133(3)	0.076(2)	−0.007(2)	0.0217(19)	−0.029(2)
N3	0.0384(15)	0.0624(19)	0.0580(19)	−0.0012(15)	−0.0034(14)	0.0004(14)
C3	0.043(2)	0.068(3)	0.063(2)	0.005(2)	0.0043(17)	0.0009(18)
N4	0.0379(15)	0.064(2)	0.0572(18)	−0.0036(15)	−0.0033(13)	0.0045(14)
O4	0.0456(15)	0.104(2)	0.087(2)	0.0100(17)	−0.0035(14)	0.0190(16)
C4	0.0428(19)	0.066(3)	0.060(2)	0.0033(19)	0.0016(16)	0.0001(18)
C5	0.046(2)	0.071(3)	0.066(2)	0.002(2)	0.0080(18)	−0.0016(19)
C6	0.053(2)	0.068(3)	0.061(2)	−0.0026(19)	0.0088(18)	0.0005(19)
C7	0.0425(19)	0.058(2)	0.058(2)	0.0022(18)	0.0056(16)	0.0018(17)
C8	0.0446(19)	0.059(2)	0.058(2)	0.0000(18)	0.0034(17)	0.0029(17)
C9	0.045(2)	0.065(3)	0.061(2)	−0.0066(19)	−0.0005(17)	−0.0023(18)
C10	0.045(2)	0.062(2)	0.058(2)	0.0006(18)	0.0026(16)	−0.0033(18)
C11	0.0407(18)	0.060(2)	0.056(2)	0.0022(18)	0.0030(16)	−0.0002(17)
C12	0.0399(18)	0.061(2)	0.062(2)	0.0031(19)	0.0020(16)	0.0036(17)
C13	0.042(2)	0.094(3)	0.060(2)	0.002(2)	0.0022(17)	0.001(2)
C14	0.055(2)	0.113(4)	0.072(3)	−0.006(3)	0.008(2)	−0.013(3)
C15	0.053(3)	0.148(6)	0.090(4)	−0.008(4)	0.007(2)	−0.020(3)
C16	0.046(3)	0.190(7)	0.076(3)	−0.019(4)	0.006(2)	0.001(4)
C17	0.065(3)	0.146(5)	0.084(3)	−0.006(3)	0.011(3)	0.028(4)

C18	0.053(2)	0.114(4)	0.079(3)	−0.002(3)	0.014(2)	0.010(3)
C19	0.0401(19)	0.072(3)	0.056(2)	−0.003(2)	−0.0003(16)	0.0026(18)
C20	0.058(2)	0.083(3)	0.067(3)	0.006(2)	0.005(2)	0.000(2)
C21	0.060(3)	0.115(4)	0.061(3)	0.009(3)	0.006(2)	0.009(3)
C22	0.053(2)	0.133(5)	0.059(3)	−0.013(3)	−0.002(2)	0.007(3)
C23	0.043(2)	0.100(3)	0.076(3)	−0.020(3)	−0.006(2)	0.002(2)
C24	0.0387(18)	0.078(3)	0.062(2)	−0.008(2)	−0.0021(16)	0.0034(19)
C25	0.0419(19)	0.061(2)	0.069(2)	−0.0020(19)	0.0051(17)	0.0000(18)
C26	0.046(2)	0.073(3)	0.067(3)	−0.001(2)	0.0043(18)	−0.0041(19)
C27	0.043(2)	0.070(3)	0.071(3)	0.000(2)	0.0020(18)	−0.0031(18)
C28	0.047(2)	0.089(3)	0.075(3)	−0.003(2)	−0.0019(19)	−0.004(2)
C29	0.050(2)	0.094(3)	0.078(3)	−0.002(3)	−0.005(2)	−0.005(2)
C30	0.069(3)	0.076(3)	0.067(3)	−0.005(2)	0.001(2)	−0.009(2)
C31	0.055(2)	0.081(3)	0.080(3)	−0.007(2)	0.006(2)	−0.004(2)
C32	0.046(2)	0.072(3)	0.076(3)	−0.006(2)	0.0009(19)	−0.004(2)
C33	0.106(4)	0.114(5)	0.082(3)	−0.017(3)	0.021(3)	−0.016(3)
C34	0.052(2)	0.065(3)	0.057(2)	−0.0045(19)	0.0022(17)	−0.0090(19)
C35	0.050(2)	0.068(3)	0.060(2)	−0.0035(19)	0.0059(17)	−0.0063(19)
C36	0.051(2)	0.064(3)	0.064(2)	−0.0055(19)	0.0108(18)	−0.0101(18)
C37	0.050(2)	0.070(3)	0.075(3)	−0.006(2)	0.0067(19)	−0.0034(19)
C38	0.053(2)	0.077(3)	0.086(3)	0.001(2)	0.014(2)	−0.007(2)
C39	0.056(3)	0.079(3)	0.094(3)	−0.008(3)	0.031(2)	−0.010(2)
C40	0.085(3)	0.097(4)	0.070(3)	−0.012(3)	0.027(2)	−0.024(3)
C41	0.062(3)	0.086(3)	0.072(3)	−0.008(2)	0.014(2)	−0.019(2)
C42	0.058(3)	0.146(6)	0.142(5)	−0.003(4)	0.044(3)	0.005(3)
C43	0.0419(19)	0.060(2)	0.061(2)	0.0036(18)	−0.0059(17)	0.0001(17)
C44	0.043(2)	0.065(2)	0.063(2)	0.0065(19)	−0.0027(17)	0.0042(18)
C45	0.047(2)	0.058(2)	0.067(2)	0.0017(19)	−0.0077(18)	0.0033(18)
C46	0.0352(18)	0.061(2)	0.064(2)	−0.0010(18)	−0.0053(16)	0.0035(16)
C47	0.0366(18)	0.069(3)	0.069(3)	0.001(2)	−0.0030(17)	0.0039(17)
C48	0.0389(18)	0.070(3)	0.065(2)	−0.004(2)	0.0019(17)	0.0059(18)
C49	0.0381(18)	0.067(2)	0.060(2)	−0.0024(19)	−0.0021(16)	0.0007(17)
C50	0.043(2)	0.069(3)	0.058(2)	−0.0044(19)	−0.0025(16)	0.0023(18)
C51	0.045(2)	0.071(3)	0.057(2)	0.0027(19)	−0.0038(17)	0.0085(19)
C52	0.0425(19)	0.065(2)	0.055(2)	−0.0030(18)	−0.0029(16)	0.0066(18)
C53	0.0367(18)	0.062(2)	0.059(2)	−0.0028(18)	−0.0059(16)	0.0040(17)
C54	0.0419(19)	0.059(2)	0.060(2)	−0.0038(18)	−0.0045(17)	0.0033(17)
C55	0.0382(18)	0.071(3)	0.061(2)	0.0027(19)	−0.0050(16)	0.0053(18)
C56	0.046(2)	0.071(3)	0.069(2)	0.004(2)	−0.0100(18)	0.0042(19)
C57	0.057(2)	0.087(3)	0.074(3)	0.001(2)	−0.013(2)	0.018(2)
C58	0.041(2)	0.113(4)	0.081(3)	0.000(3)	−0.008(2)	0.008(3)
C59	0.042(2)	0.113(4)	0.081(3)	0.011(3)	−0.007(2)	−0.008(2)
C60	0.046(2)	0.084(3)	0.085(3)	0.015(2)	−0.010(2)	−0.003(2)
C61	0.0405(19)	0.080(3)	0.063(2)	0.000(2)	−0.0033(17)	0.005(2)
C62	0.068(3)	0.089(4)	0.070(3)	0.005(2)	0.009(2)	0.012(3)
C63	0.074(3)	0.100(4)	0.093(4)	0.022(3)	0.018(3)	0.009(3)
C64	0.066(3)	0.121(5)	0.077(3)	0.026(3)	0.018(2)	0.020(3)
C65	0.059(3)	0.113(4)	0.074(3)	−0.011(3)	0.008(2)	0.015(3)
C66	0.049(2)	0.093(3)	0.070(3)	−0.004(2)	0.0062(19)	0.005(2)
C67	0.0409(19)	0.066(3)	0.062(2)	0.0059(19)	−0.0051(17)	0.0005(17)
C68	0.046(2)	0.069(3)	0.068(3)	0.005(2)	−0.0036(19)	0.0032(18)
C69	0.058(2)	0.058(2)	0.065(2)	0.0068(19)	−0.0014(19)	−0.0014(19)
C70	0.061(2)	0.077(3)	0.062(3)	0.006(2)	−0.005(2)	−0.007(2)

C71	0.073(3)	0.088(3)	0.063(3)	0.005(2)	0.000(2)	−0.018(2)
C72	0.068(3)	0.091(3)	0.069(3)	0.005(2)	0.013(2)	−0.017(2)
C73	0.055(2)	0.088(3)	0.076(3)	0.007(2)	0.006(2)	−0.009(2)
C74	0.056(2)	0.075(3)	0.064(3)	0.006(2)	0.003(2)	−0.002(2)
C75	0.107(5)	0.201(8)	0.084(4)	−0.003(4)	0.014(3)	−0.030(5)
C76	0.0416(19)	0.070(3)	0.057(2)	−0.0023(19)	0.0034(16)	0.0069(18)
C77	0.0417(19)	0.067(3)	0.057(2)	0.0013(19)	−0.0028(16)	0.0055(18)
C78	0.0414(19)	0.072(3)	0.054(2)	0.0014(19)	−0.0040(16)	0.0061(18)
C79	0.045(2)	0.068(3)	0.067(2)	0.005(2)	−0.0077(17)	0.0027(19)
C80	0.055(2)	0.069(3)	0.077(3)	0.007(2)	−0.010(2)	0.009(2)
C81	0.042(2)	0.084(3)	0.064(2)	0.004(2)	−0.0045(17)	0.011(2)
C82	0.046(2)	0.090(3)	0.071(3)	0.013(2)	0.0003(19)	0.006(2)
C83	0.048(2)	0.074(3)	0.071(3)	0.013(2)	−0.0001(19)	0.008(2)
C84	0.050(2)	0.148(5)	0.076(3)	0.014(3)	0.007(2)	0.024(3)
B1	0.044(3)	0.100(4)	0.073(3)	−0.009(3)	−0.006(2)	−0.007(3)
F1	0.072(2)	0.194(4)	0.185(4)	0.072(3)	−0.014(2)	0.004(2)
F2	0.091(2)	0.148(3)	0.135(3)	−0.012(2)	0.050(2)	−0.013(2)
F3	0.099(2)	0.204(4)	0.098(2)	−0.042(2)	−0.0004(19)	−0.025(2)
F4	0.102(3)	0.101(3)	0.259(5)	−0.015(3)	0.067(3)	−0.023(2)
O1A	0.101(3)	0.082(3)	0.120(4)	−0.028(3)	0.059(3)	−0.018(3)
O1B	0.101(3)	0.082(3)	0.120(4)	−0.028(3)	0.059(3)	−0.018(3)
O1C	0.145(7)	0.137(6)	0.123(6)	0.011(6)	0.042(6)	0.043(5)
O1D	0.145(7)	0.137(6)	0.123(6)	0.011(6)	0.042(6)	0.043(5)

Table 4. Bond lengths and angles for Cu(**bathocupSani**)$_2$BF$_4$

Atom–Atom	Length [Å]
Cu1–N1	2.001(3)
Cu1–N2	2.075(3)
Cu1–N3	2.095(3)
Cu1–N4	2.004(3)
C1–N1	1.349(4)
C1–C2	1.405(5)
C1–C25	1.458(5)
N1–C12	1.373(5)
O1–C30	1.374(5)
O1–C33	1.432(6)
N2–C10	1.352(4)
N2–C11	1.347(4)
C2–H2	0.9500
C2–C3	1.374(6)
O2–C39	1.355(5)
O2–C42	1.460(6)
O3–C72	1.369(6)
O3–C75	1.413(6)
N3–C43	1.347(5)
N3–C54	1.346(5)
C3–C4	1.419(5)
C3–C13	1.472(6)
N4–C52	1.347(5)
N4–C53	1.370(5)
O4–C81	1.372(4)

O4–C84	1.411(6)
C4–C5	1.424(5)
C4–C12	1.401(5)
C5–H5	0.9500
C5–C6	1.349(5)
C6–H6	0.9500
C6–C7	1.429(5)
C7–C8	1.416(5)
C7–C11	1.408(5)
C8–C9	1.367(5)
C8–C19	1.491(5)
C9–H9	0.9500
C9–C10	1.405(5)
C10–C34	1.446(5)
C11–C12	1.448(5)
C13–C14	1.371(6)
C13–C18	1.414(7)
C14–H14	0.9500
C14–C15	1.387(7)
C15–H15	0.9500
C15–C16	1.344(9)
C16–H16	0.9500
C16–C17	1.368(9)
C17–H17	0.9500
C17–C18	1.392(6)
C18–H18	0.9500

C19–C20	1.390(6)
C19–C24	1.380(6)
C20–H20	0.9500
C20–C21	1.385(6)
C21–H21	0.9500
C21–C22	1.369(7)
C22–H22	0.9500
C22–C23	1.384(7)
C23–H23	0.9500
C23–C24	1.382(6)
C24–H24	0.9500
C25–H25	0.9500
C25–C26	1.342(5)
C26–H26	0.9500
C26–C27	1.458(6)
C27–C28	1.414(5)
C27–C32	1.382(6)
C28–H28	0.9500
C28–C29	1.377(6)
C29–H29	0.9500
C29–C30	1.371(6)
C30–C31	1.390(6)
C31–H31	0.9500
C31–C32	1.383(6)
C32–H32	0.9500
C33–H33A	0.9800
C33–H33B	0.9800
C33–H33C	0.9800
C34–H34	0.9500
C34–C35	1.334(5)
C35–H35	0.9500
C35–C36	1.442(5)
C36–C37	1.388(5)
C36–C41	1.410(6)
C37–H37	0.9500
C37–C38	1.373(6)
C38–H38	0.9500
C38–C39	1.376(6)
C39–C40	1.396(7)
C40–H40	0.9500
C40–C41	1.373(6)
C41–H41	0.9500
C42–H42A	0.9800
C42–H42B	0.9800
C42–H42C	0.9800
C43–C44	1.418(5)
C43–C67	1.443(6)
C44–H44	0.9500
C44–C45	1.351(6)
C45–C46	1.432(5)
C45–C55	1.496(5)
C46–C47	1.413(6)
C46–C54	1.418(5)

C47–H47	0.9500
C47–C48	1.350(5)
C48–H48	0.9500
C48–C49	1.430(5)
C49–C50	1.423(5)
C49–C53	1.399(5)
C50–C51	1.369(5)
C50–C61	1.484(6)
C51–H51	0.9500
C51–C52	1.407(5)
C52–C76	1.452(5)
C53–C54	1.442(5)
C55–C56	1.380(6)
C55–C60	1.389(6)
C56–H56	0.9500
C56–C57	1.388(5)
C57–H57	0.9500
C57–C58	1.367(7)
C58–H58	0.9500
C58–C59	1.380(7)
C59–H59	0.9500
C59–C60	1.383(6)
C60–H60	0.9500
C61–C62	1.390(6)
C61–C66	1.392(6)
C62–H62	0.9500
C62–C63	1.386(7)
C63–H63	0.9500
C63–C64	1.380(8)
C64–H64	0.9500
C64–C65	1.394(8)
C65–H65	0.9500
C65–C66	1.390(6)
C66–H66	0.9500
C67–H67	0.9500
C67–C68	1.330(5)
C68–H68	0.9500
C68–C69	1.456(6)
C69–C70	1.397(6)
C69–C74	1.402(6)
C70–H70	0.9500
C70–C71	1.371(6)
C71–H71	0.9500
C71–C72	1.385(6)
C72–C73	1.395(6)
C73–H73	0.9500
C73–C74	1.363(6)
C74–H74	0.9500
C75–H75A	0.9800
C75–H75B	0.9800
C75–H75C	0.9800
C76–H76	0.9500
C76–C77	1.337(5)

C77–H77	0.9500
C77–C78	1.461(5)
C78–C79	1.391(5)
C78–C83	1.384(6)
C79–H79	0.9500
C79–C80	1.386(5)
C80–H80	0.9500
C80–C81	1.358(6)
C81–C82	1.373(6)
C82–H82	0.9500
C82–C83	1.379(5)
C83–H83	0.9500
C84–H84A	0.9800
C84–H84B	0.9800
C84–H84C	0.9800
B1–F1	1.332(6)
B1–F2	1.352(6)
B1–F3	1.345(6)
B1–F4	1.356(7)
**Atom–Atom–Atom**	**Angle [°]**
N1–Cu1–N2	81.72(12)
N1–Cu1–N3	130.24(13)
N1–Cu1–N4	138.51(12)
N2–Cu1–N3	97.56(12)
N4–Cu1–N2	124.98(12)
N4–Cu1–N3	81.58(12)
N1–C1–C2	120.7(4)
N1–C1–C25	117.2(3)
C2–C1–C25	121.8(3)
C1–N1–Cu1	129.6(3)
C1–N1–C12	117.8(3)
C12–N1–Cu1	112.5(2)
C30–O1–C33	117.3(4)
C10–N2–Cu1	128.7(2)
C11–N2–Cu1	110.7(2)
C11–N2–C10	118.3(3)
C1–C2–H2	118.8
C3–C2–C1	122.4(4)
C3–C2–H2	118.8
C39–O2–C42	116.2(4)
C72–O3–C75	117.7(4)
C43–N3–Cu1	128.5(3)
C54–N3–Cu1	110.2(2)
C54–N3–C43	119.1(3)
C2–C3–C4	117.3(3)
C2–C3–C13	119.2(4)
C4–C3–C13	123.4(4)
C52–N4–Cu1	128.3(2)
C52–N4–C53	118.1(3)
C53–N4–Cu1	113.1(2)
C81–O4–C84	117.9(4)
C3–C4–C5	123.9(3)

C12–C4–C3	117.9(4)
C12–C4–C5	118.2(3)
C4–C5–H5	118.9
C6–C5–C4	122.2(4)
C6–C5–H5	118.9
C5–C6–H6	119.3
C5–C6–C7	121.4(4)
C7–C6–H6	119.3
C8–C7–C6	124.6(3)
C11–C7–C6	118.0(3)
C11–C7–C8	117.4(3)
C7–C8–C19	122.7(3)
C9–C8–C7	117.9(3)
C9–C8–C19	119.3(3)
C8–C9–H9	119.1
C8–C9–C10	121.7(3)
C10–C9–H9	119.1
N2–C10–C9	120.6(3)
N2–C10–C34	115.9(3)
C9–C10–C34	123.4(3)
N2–C11–C7	123.7(3)
N2–C11–C12	116.4(3)
C7–C11–C12	119.9(3)
N1–C12–C4	123.7(3)
N1–C12–C11	116.6(3)
C4–C12–C11	119.7(3)
C14–C13–C3	122.1(4)
C14–C13–C18	117.7(4)
C18–C13–C3	119.9(4)
C13–C14–H14	119.4
C13–C14–C15	121.2(5)
C15–C14–H14	119.4
C14–C15–H15	120.0
C16–C15–C14	120.0(6)
C16–C15–H15	120.0
C15–C16–H16	119.1
C15–C16–C17	121.8(5)
C17–C16–H16	119.1
C16–C17–H17	120.6
C16–C17–C18	118.7(6)
C18–C17–H17	120.6
C13–C18–H18	119.7
C17–C18–C13	120.5(5)
C17–C18–H18	119.7
C20–C19–C8	120.7(4)
C24–C19–C8	120.5(4)
C24–C19–C20	118.8(4)
C19–C20–H20	119.9
C21–C20–C19	120.2(4)
C21–C20–H20	119.9
C20–C21–H21	119.8
C22–C21–C20	120.3(5)
C22–C21–H21	119.8

C21–C22–H22	119.9
C21–C22–C23	120.1(4)
C23–C22–H22	119.9
C22–C23–H23	120.3
C24–C23–C22	119.4(5)
C24–C23–H23	120.3
C19–C24–C23	121.1(4)
C19–C24–H24	119.4
C23–C24–H24	119.4
C1–C25–H25	118.2
C26–C25–C1	123.7(4)
C26–C25–H25	118.2
C25–C26–H26	116.1
C25–C26–C27	127.8(4)
C27–C26–H26	116.1
C28–C27–C26	118.2(4)
C32–C27–C26	124.6(4)
C32–C27–C28	117.2(4)
C27–C28–H28	119.6
C29–C28–C27	120.7(4)
C29–C28–H28	119.6
C28–C29–H29	119.7
C30–C29–C28	120.5(4)
C30–C29–H29	119.7
O1–C30–C31	124.2(4)
C29–C30–O1	115.8(4)
C29–C30–C31	120.0(4)
C30–C31–H31	120.4
C32–C31–C30	119.2(4)
C32–C31–H31	120.4
C27–C32–C31	122.1(4)
C27–C32–H32	118.9
C31–C32–H32	118.9
O1–C33–H33A	109.5
O1–C33–H33B	109.5
O1–C33–H33C	109.5
H33A–C33–H33B	109.5
H33A–C33–H33C	109.5
H33B–C33–H33C	109.5
C10–C34–H34	117.1
C35–C34–C10	125.8(4)
C35–C34–H34	117.1
C34–C35–H35	116.0
C34–C35–C36	128.0(4)
C36–C35–H35	116.0
C37–C36–C35	119.5(4)
C37–C36–C41	117.1(4)
C41–C36–C35	123.4(4)
C36–C37–H37	118.6
C38–C37–C36	122.9(4)
C38–C37–H37	118.6
C37–C38–H38	120.3
C37–C38–C39	119.4(4)

C39–C38–H38	120.3
O2–C39–C38	125.1(4)
O2–C39–C40	115.5(4)
C38–C39–C40	119.4(4)
C39–C40–H40	119.5
C41–C40–C39	120.9(4)
C41–C40–H40	119.5
C36–C41–H41	119.9
C40–C41–C36	120.3(4)
C40–C41–H41	119.9
O2–C42–H42A	109.5
O2–C42–H42B	109.5
O2–C42–H42C	109.5
H42A–C42–H42B	109.5
H42A–C42–H42C	109.5
H42B–C42–H42C	109.5
N3–C43–C44	120.4(4)
N3–C43–C67	116.2(3)
C44–C43–C67	123.3(3)
C43–C44–H44	119.3
C45–C44–C43	121.4(4)
C45–C44–H44	119.3
C44–C45–C46	119.1(3)
C44–C45–C55	122.5(3)
C46–C45–C55	118.4(4)
C47–C46–C45	124.8(3)
C47–C46–C54	119.0(3)
C54–C46–C45	116.2(4)
C46–C47–H47	119.0
C48–C47–C46	122.0(3)
C48–C47–H47	119.0
C47–C48–H48	119.7
C47–C48–C49	120.6(4)
C49–C48–H48	119.7
C50–C49–C48	123.0(4)
C53–C49–C48	119.2(3)
C53–C49–C50	117.8(3)
C49–C50–C61	121.7(3)
C51–C50–C49	118.0(4)
C51–C50–C61	120.4(3)
C50–C51–H51	119.2
C50–C51–C52	121.7(4)
C52–C51–H51	119.2
N4–C52–C51	121.0(3)
N4–C52–C76	116.4(3)
C51–C52–C76	122.4(3)
N4–C53–C49	123.5(3)
N4–C53–C54	116.4(3)
C49–C53–C54	120.1(3)
N3–C54–C46	123.6(3)
N3–C54–C53	117.7(3)
C46–C54–C53	118.6(4)
C56–C55–C45	121.5(4)

C56–C55–C60	119.1(4)
C60–C55–C45	119.4(4)
C55–C56–H56	119.9
C55–C56–C57	120.1(4)
C57–C56–H56	119.9
C56–C57–H57	120.1
C58–C57–C56	119.9(4)
C58–C57–H57	120.1
C57–C58–H58	119.5
C57–C58–C59	121.1(4)
C59–C58–H58	119.5
C58–C59–H59	120.6
C58–C59–C60	118.8(4)
C60–C59–H59	120.6
C55–C60–H60	119.5
C59–C60–C55	121.0(4)
C59–C60–H60	119.5
C62–C61–C50	119.7(4)
C62–C61–C66	119.6(4)
C66–C61–C50	120.6(4)
C61–C62–H62	119.5
C63–C62–C61	120.9(5)
C63–C62–H62	119.5
C62–C63–H63	120.5
C64–C63–C62	119.1(5)
C64–C63–H63	120.5
C63–C64–H64	119.6
C63–C64–C65	120.9(5)
C65–C64–H64	119.6
C64–C65–H65	120.2
C66–C65–C64	119.7(5)
C66–C65–H65	120.2
C61–C66–H66	120.1
C65–C66–C61	119.7(5)
C65–C66–H66	120.1
C43–C67–H67	117.0
C68–C67–C43	125.9(3)
C68–C67–H67	117.0
C67–C68–H68	116.0
C67–C68–C69	127.9(4)
C69–C68–H68	116.0
C70–C69–C68	120.6(4)
C70–C69–C74	116.3(4)
C74–C69–C68	123.0(4)
C69–C70–H70	118.5
C71–C70–C69	123.0(4)
C71–C70–H70	118.5
C70–C71–H71	120.2
C70–C71–C72	119.5(4)
C72–C71–H71	120.2
O3–C72–C71	126.3(4)

O3–C72–C73	115.1(4)
C71–C72–C73	118.7(4)
C72–C73–H73	119.4
C74–C73–C72	121.2(4)
C74–C73–H73	119.4
C69–C74–H74	119.4
C73–C74–C69	121.3(4)
C73–C74–H74	119.4
O3–C75–H75A	109.5
O3–C75–H75B	109.5
O3–C75–H75C	109.5
H75A–C75–H75B	109.5
H75A–C75–H75C	109.5
H75B–C75–H75C	109.5
C52–C76–H76	117.0
C77–C76–C52	126.0(4)
C77–C76–H76	117.0
C76–C77–H77	117.5
C76–C77–C78	125.0(4)
C78–C77–H77	117.5
C79–C78–C77	120.5(4)
C83–C78–C77	122.0(4)
C83–C78–C79	117.5(3)
C78–C79–H79	119.8
C80–C79–C78	120.3(4)
C80–C79–H79	119.8
C79–C80–H80	119.6
C81–C80–C79	120.8(4)
C81–C80–H80	119.6
O4–C81–C82	124.4(4)
C80–C81–O4	115.6(4)
C80–C81–C82	120.0(4)
C81–C82–H82	120.3
C81–C82–C83	119.5(4)
C83–C82–H82	120.3
C78–C83–H83	119.1
C82–C83–C78	121.8(4)
C82–C83–H83	119.1
O4–C84–H84A	109.5
O4–C84–H84B	109.5
O4–C84–H84C	109.5
H84A–C84–H84B	109.5
H84A–C84–H84C	109.5
H84B–C84–H84C	109.5
F1–B1–F2	113.5(5)
F1–B1–F3	108.2(5)
F1–B1–F4	108.1(4)
F2–B1–F4	112.3(5)
F3–B1–F2	106.5(4)
F3–B1–F4	108.0(5)

Table 5. Torsion angles for Cu(**bathocupSani**)$_2$BF$_4$

Atom–Atom–Atom–Atom	Torsion Angle [°]
Cu1–N1–C12–C4	177.2(3)
Cu1–N1–C12–C11	−3.9(4)
Cu1–N2–C10–C9	156.7(3)
Cu1–N2–C10–C34	−20.4(5)
Cu1–N2–C11–C7	−164.5(3)
Cu1–N2–C11–C12	14.5(4)
Cu1–N3–C43–C44	160.7(3)
Cu1–N3–C43–C67	−16.7(5)
Cu1–N3–C54–C46	−166.9(3)
Cu1–N3–C54–C53	9.9(4)
Cu1–N4–C52–C51	−171.6(3)
Cu1–N4–C52–C76	12.6(5)
Cu1–N4–C53–C49	174.5(3)
Cu1–N4–C53–C54	−3.3(4)
C1–N1–C12–C4	−1.4(5)
C1–N1–C12–C11	177.4(3)
C1–C2–C3–C4	3.6(6)
C1–C2–C3–C13	−173.1(4)
C1–C25–C26–C27	−171.1(4)
N1–C1–C2–C3	−1.6(6)
N1–C1–C25–C26	160.7(4)
O1–C30–C31–C32	−174.6(4)
N2–C10–C34–C35	163.7(4)
N2–C11–C12–N1	−7.6(5)
N2–C11–C12–C4	171.4(3)
C2–C1–N1–Cu1	−178.0(3)
C2–C1–N1–C12	0.4(5)
C2–C1–C25–C26	−13.5(6)
C2–C3–C4–C5	174.5(4)
C2–C3–C4–C12	−4.4(6)
C2–C3–C13–C14	−47.1(6)
C2–C3–C13–C18	126.2(4)
O2–C39–C40–C41	−179.3(4)
O3–C72–C73–C74	−179.3(4)
N3–C43–C44–C45	0.5(6)
N3–C43–C67–C68	156.2(4)
C3–C4–C5–C6	−179.3(4)
C3–C4–C12–N1	3.5(6)
C3–C4–C12–C11	−175.3(3)
C3–C13–C14–C15	172.5(4)
C3–C13–C18–C17	−172.9(4)
N4–C52–C76–C77	−173.0(4)
N4–C53–C54–N3	−4.8(5)
N4–C53–C54–C46	172.1(3)
O4–C81–C82–C83	178.5(4)
C4–C3–C13–C14	136.4(4)
C4–C3–C13–C18	−50.3(6)
C4–C5–C6–C7	−1.3(6)
C5–C4–C12–N1	−175.4(4)
C5–C4–C12–C11	5.7(6)
C5–C6–C7–C8	179.0(4)
C5–C6–C7–C11	−2.5(6)
C6–C7–C8–C9	172.7(4)
C6–C7–C8–C19	−9.5(6)
C6–C7–C11–N2	−173.2(4)
C6–C7–C11–C12	7.8(5)
C7–C8–C9–C10	1.7(6)
C7–C8–C19–C20	−48.4(5)
C7–C8–C19–C24	134.2(4)
C7–C11–C12–N1	171.5(3)
C7–C11–C12–C4	−9.6(6)
C8–C7–C11–N2	5.4(6)
C8–C7–C11–C12	−173.5(3)
C8–C9–C10–N2	3.5(6)
C8–C9–C10–C34	−179.5(4)
C8–C19–C20–C21	−176.1(4)
C8–C19–C24–C23	177.3(3)
C9–C8–C19–C20	129.4(4)
C9–C8–C19–C24	−48.0(5)
C9–C10–C34–C35	−13.3(7)
C10–N2–C11–C7	−0.4(5)
C10–N2–C11–C12	178.6(3)
C10–C34–C35–C36	−176.5(4)
C11–N2–C10–C9	−4.2(5)
C11–N2–C10–C34	178.7(3)
C11–C7–C8–C9	−5.9(5)
C11–C7–C8–C19	171.9(4)
C12–C4–C5–C6	−0.4(6)
C13–C3–C4–C5	−9.0(6)
C13–C3–C4–C12	172.1(4)
C13–C14–C15–C16	0.2(8)
C14–C13–C18–C17	0.7(7)
C14–C15–C16–C17	0.8(8)
C15–C16–C17–C18	−1.0(8)
C16–C17–C18–C13	0.2(7)
C18–C13–C14–C15	−0.9(7)
C19–C8–C9–C10	−176.2(4)
C19–C20–C21–C22	−1.4(7)
C20–C19–C24–C23	−0.1(6)
C20–C21–C22–C23	0.2(7)
C21–C22–C23–C24	1.0(7)
C22–C23–C24–C19	−1.1(6)
C24–C19–C20–C21	1.4(6)
C25–C1–N1–Cu1	7.7(5)
C25–C1–N1–C12	−173.9(3)
C25–C1–C2–C3	172.5(4)
C25–C26–C27–C28	171.0(4)
C25–C26–C27–C32	−5.7(7)
C26–C27–C28–C29	−174.0(4)
C26–C27–C32–C31	174.4(4)

C27–C28–C29–C30	0.1(7)
C28–C27–C32–C31	−2.4(7)
C28–C29–C30–O1	175.2(4)
C28–C29–C30–C31	−3.7(7)
C29–C30–C31–C32	4.2(7)
C30–C31–C32–C27	−1.1(7)
C32–C27–C28–C29	3.0(7)
C33–O1–C30–C29	−171.2(5)
C33–O1–C30–C31	7.7(7)
C34–C35–C36–C37	167.7(4)
C34–C35–C36–C41	−8.6(7)
C35–C36–C37–C38	−175.1(4)
C35–C36–C41–C40	174.3(4)
C36–C37–C38–C39	0.8(7)
C37–C36–C41–C40	−2.1(6)
C37–C38–C39–O2	178.7(4)
C37–C38–C39–C40	−2.3(7)
C38–C39–C40–C41	1.6(7)
C39–C40–C41–C36	0.6(7)
C41–C36–C37–C38	1.4(6)
C42–O2–C39–C38	−6.0(7)
C42–O2–C39–C40	175.0(5)
C43–N3–C54–C46	−2.2(6)
C43–N3–C54–C53	174.5(3)
C43–C44–C45–C46	2.8(6)
C43–C44–C45–C55	−175.3(4)
C43–C67–C68–C69	−175.7(4)
C44–C43–C67–C68	−21.0(6)
C44–C45–C46–C47	174.5(4)
C44–C45–C46–C54	−5.5(5)
C44–C45–C55–C56	−57.9(6)
C44–C45–C55–C60	124.0(5)
C45–C46–C47–C48	175.0(4)
C45–C46–C54–N3	5.4(6)
C45–C46–C54–C53	−171.4(3)
C45–C55–C56–C57	−176.2(4)
C45–C55–C60–C59	176.5(4)
C46–C45–C55–C56	123.9(4)
C46–C45–C55–C60	−54.1(5)
C46–C47–C48–C49	−1.7(6)
C47–C46–C54–N3	−174.6(4)
C47–C46–C54–C53	8.6(5)
C47–C48–C49–C50	−172.5(4)
C47–C48–C49–C53	4.7(6)
C48–C49–C50–C51	176.6(4)
C48–C49–C50–C61	−4.4(6)
C48–C49–C53–N4	−178.6(4)
C48–C49–C53–C54	−0.9(6)
C49–C50–C51–C52	2.3(6)
C49–C50–C61–C62	117.9(4)
C49–C50–C61–C66	−65.2(5)
C49–C53–C54–N3	177.3(3)
C49–C53–C54–C46	−5.7(6)

C50–C49–C53–N4	−1.2(6)
C50–C49–C53–C54	176.5(4)
C50–C51–C52–N4	−2.2(6)
C50–C51–C52–C76	173.4(4)
C50–C61–C62–C63	176.2(4)
C50–C61–C66–C65	−174.3(4)
C51–C50–C61–C62	−63.2(5)
C51–C50–C61–C66	113.7(4)
C51–C52–C76–C77	11.2(6)
C52–N4–C53–C49	1.4(5)
C52–N4–C53–C54	−176.4(3)
C52–C76–C77–C78	−173.0(4)
C53–N4–C52–C51	0.3(5)
C53–N4–C52–C76	−175.5(3)
C53–C49–C50–C51	−0.6(6)
C53–C49–C50–C61	178.4(4)
C54–N3–C43–C44	−0.9(5)
C54–N3–C43–C67	−178.2(3)
C54–C46–C47–C48	−5.0(6)
C55–C45–C46–C47	−7.3(6)
C55–C45–C46–C54	172.7(3)
C55–C56–C57–C58	−0.6(6)
C56–C55–C60–C59	−1.6(7)
C56–C57–C58–C59	−1.0(7)
C57–C58–C59–C60	1.3(7)
C58–C59–C60–C55	0.0(7)
C60–C55–C56–C57	1.9(6)
C61–C50–C51–C52	−176.7(4)
C61–C62–C63–C64	−1.9(7)
C62–C61–C66–C65	2.6(6)
C62–C63–C64–C65	2.6(7)
C63–C64–C65–C66	−0.7(7)
C64–C65–C66–C61	−1.9(6)
C66–C61–C62–C63	−0.7(6)
C67–C43–C44–C45	177.6(4)
C67–C68–C69–C70	176.1(4)
C67–C68–C69–C74	−1.4(7)
C68–C69–C70–C71	−176.1(4)
C68–C69–C74–C73	176.6(4)
C69–C70–C71–C72	−1.0(7)
C70–C69–C74–C73	−1.0(6)
C70–C71–C72–O3	179.8(5)
C70–C71–C72–C73	−0.1(7)
C71–C72–C73–C74	0.7(7)
C72–C73–C74–C69	−0.1(7)
C74–C69–C70–C71	1.6(6)
C75–O3–C72–C71	−2.3(8)
C75–O3–C72–C73	177.6(5)
C76–C77–C78–C79	−156.4(4)
C76–C77–C78–C83	27.0(6)
C77–C78–C79–C80	−178.6(4)
C77–C78–C83–C82	177.9(4)
C78–C79–C80–C81	0.7(6)

C79–C78–C83–C82	1.1(6)
C79–C80–C81–O4	−179.1(4)
C79–C80–C81–C82	1.3(7)
C80–C81–C82–C83	−2.1(7)
C81–C82–C83–C78	0.8(7)
C83–C78–C79–C80	−1.8(6)
C84–O4–C81–C80	170.1(4)
C84–O4–C81–C82	−10.4(6)

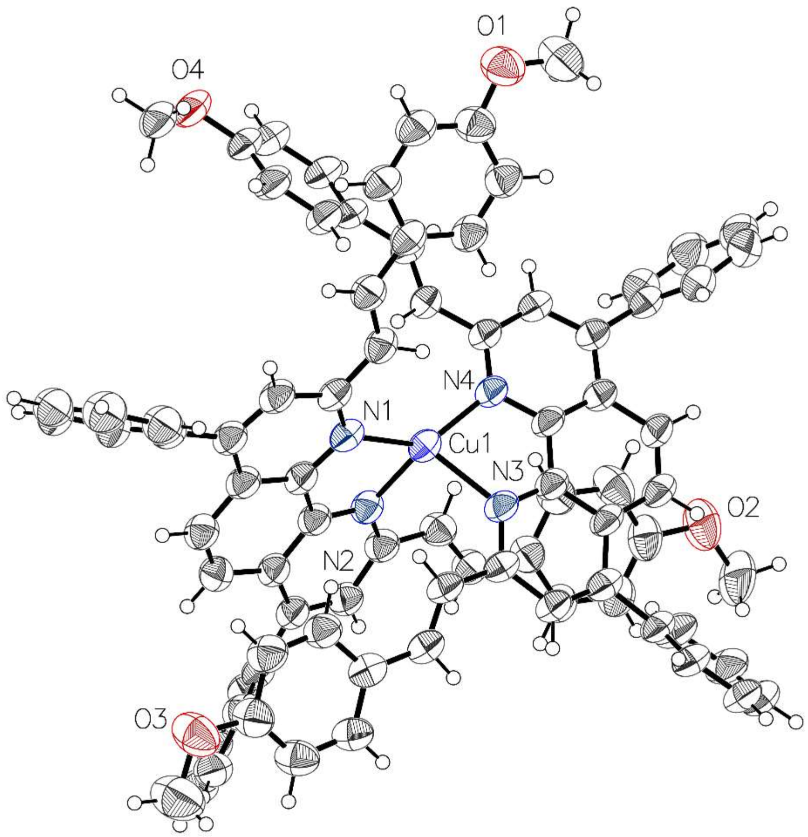

Figure S10: Thermal atomic displacement ellipsoid plot for the main complex of Cu(**bathocupSani**)$_2$BF$_4$ with partial numbering scheme

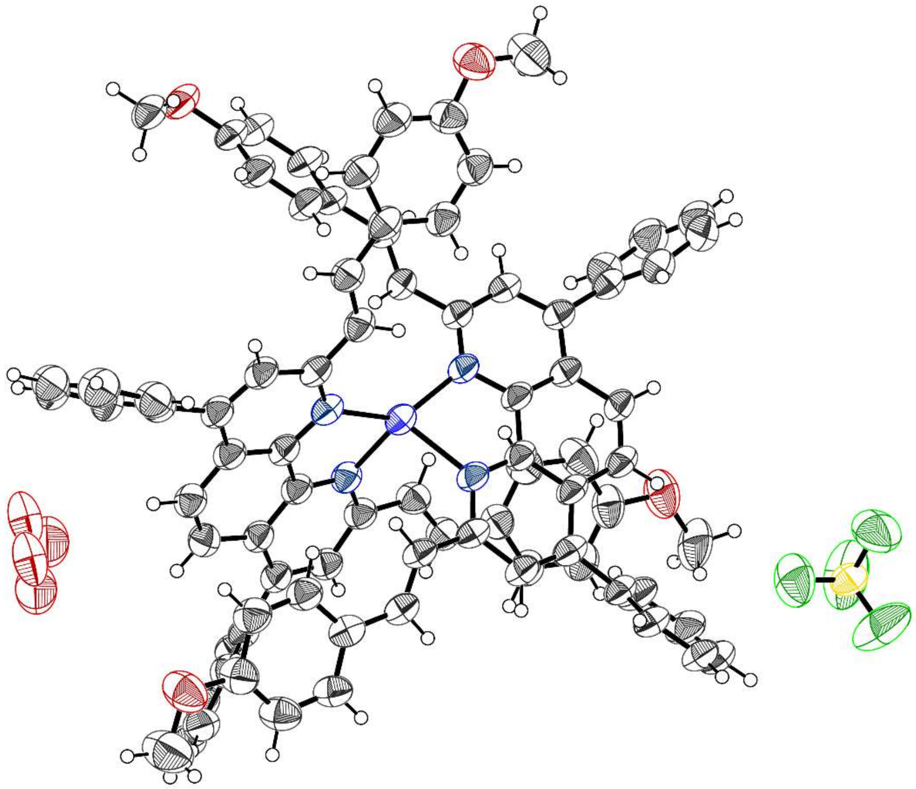

Figure S11: Thermal atomic displacement ellipsoid plot for Cu(**bathocupSani**)$_2BF_4$ including counter ion and disordered water solvent molecules. Ellipsoids are drawn at the 50% probability level and hydrogen atoms are shown as sphere of arbitrary size.

COMPUTATIONAL DETAILS AND METHODS

Theoretical calculations were performed using density functional theory (DFT) to calculate the nature of the lowest energy unoccupied molecular orbitals (LUMOs) and the highest energy molecular orbitals (HOMOs) for both complexes (computed frontier molecular orbitals shown in Table 6). The ground state optimization and energy calculations were carried out with Gaussian using the B3PW91 functional with dgdzvp as basis set. The optimizations were conducted without symmetry constraint, and frequency calculations were made to confirm reaching the energy minima. GaussView was used for data analysis, visualization and plots. All calculations were conducted for gaseous phase complexes. TD-DFT calculations were performed using with Gaussian using the b3lyp as functional with 6 – 31g as basis set with acetonitrile as continuous solvent model.

Table 1. Calculated MOs of copper complexes (Isovalue = 0.03).

<table>
<tr><td colspan="2">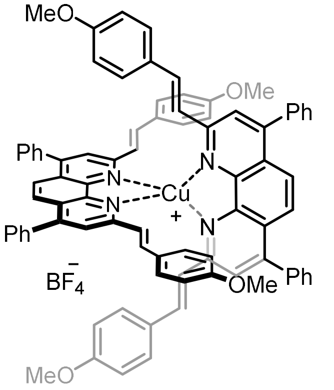
</td><td colspan="2">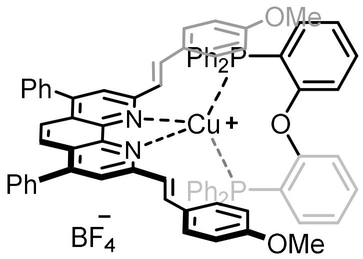
</td></tr>
<tr><td>MOs</td><td>Orbitals</td><td>MOs</td><td>Orbitals</td></tr>
<tr><td>LUMO + 6 (335)</td><td>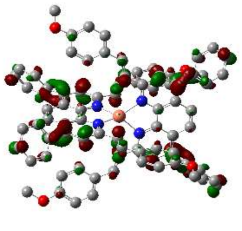</td><td>LUMO + 6 (320)</td><td>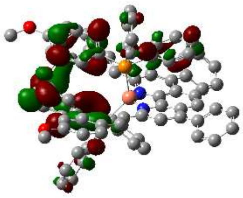</td></tr>
<tr><td>LUMO + 5 (334)</td><td>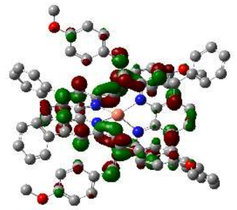</td><td>LUMO + 5 (319)</td><td>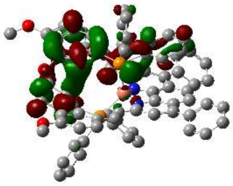</td></tr>
</table>

LUMO + 4 (333)	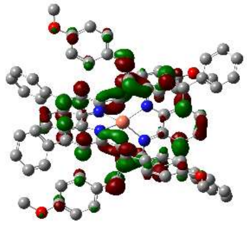	LUMO + 4 (318)	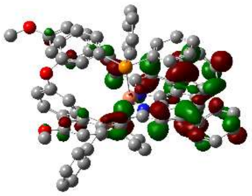
LUMO + 3 (332)	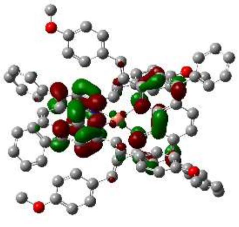	LUMO + 3 (317)	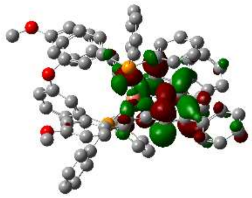
LUMO + 2 (331)	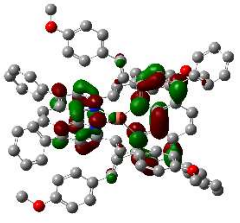	LUMO + 2 (316)	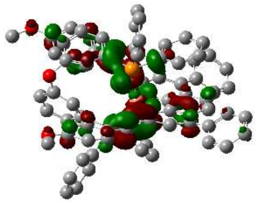
LUMO +1 (330)	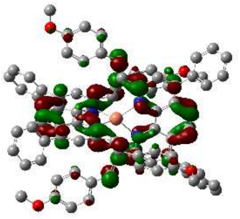	LUMO +1 (315)	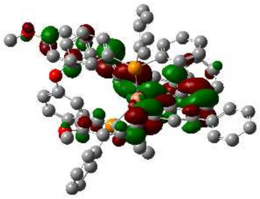
LUMO (329)	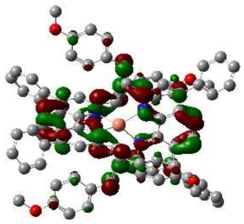	LUMO (314)	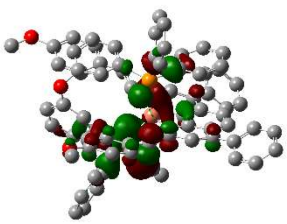

HOMO (328)	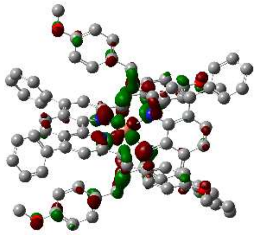	HOMO (313)	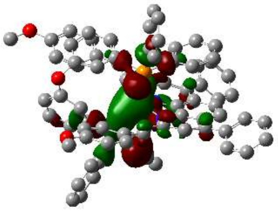
HOMO − 1 (327)	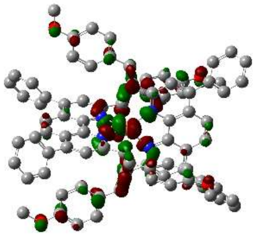	HOMO − 1 (312)	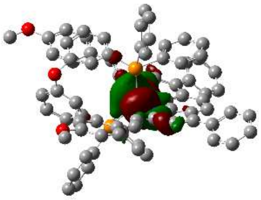
HOMO − 2 (326)	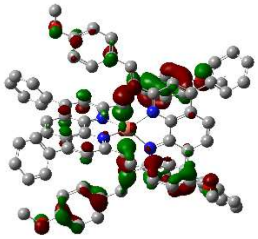	HOMO − 2 (311)	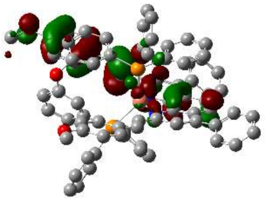
HOMO − 3 (325)	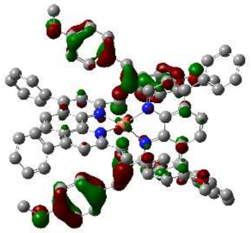	HOMO − 3 (310)	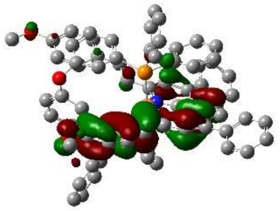
HOMO − 4 (324)	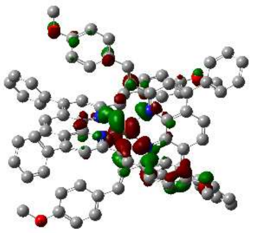	HOMO − 4 (309)	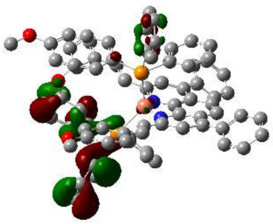

HOMO – 5 (323)	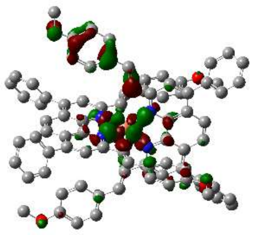	HOMO – 5 (308)	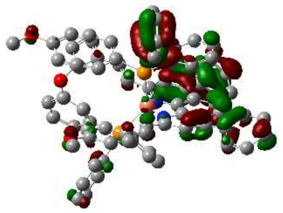
HOMO – 6 (322)	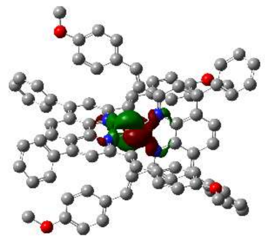	HOMO – 6 (307)	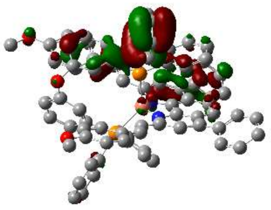

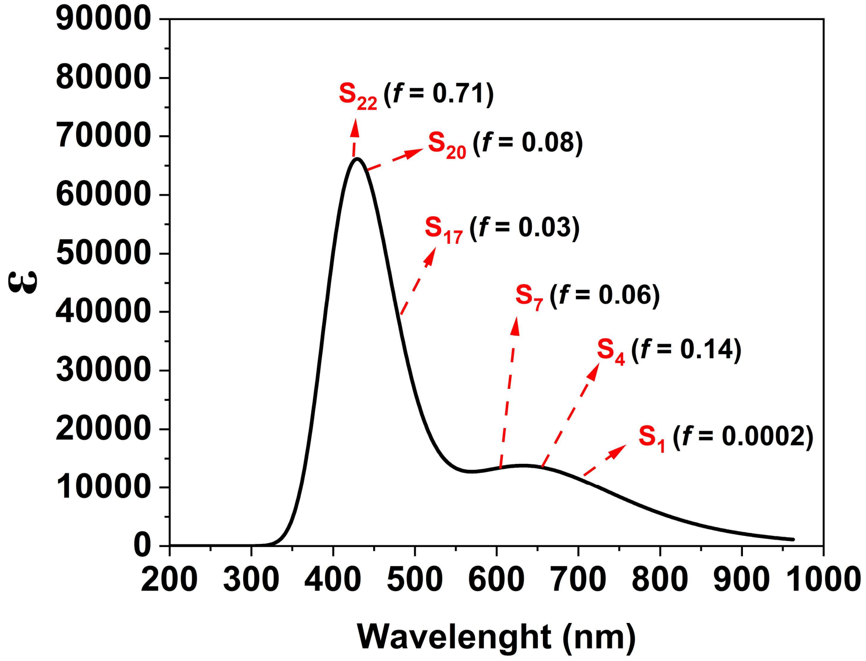

Figure S12: Calculated UV-Vis spectra of homoleptic complex Cu(**bathocupSani**)$_2$BF$_4$ along with selected singlet electronic transitions.

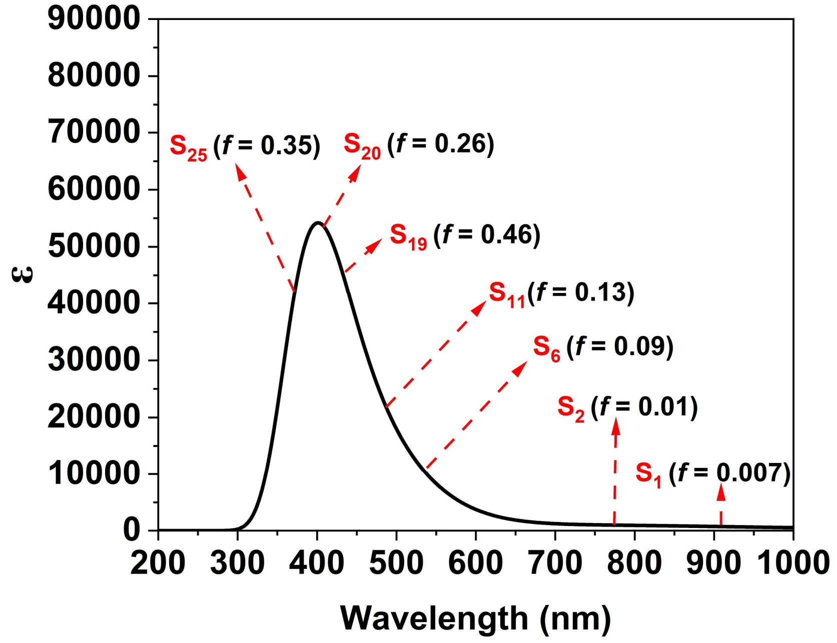

Figure S13: Calculated UV-Vis spectra of heteroleptic complex Cu(**bathocupSani**)(**DPEPhos**)BF_4 along with selected singlet electronic transitions.

Table 2. Calculated selected singlet electronic transitions with oscillator strength ($f > 0.01$) of homoleptic complex Cu(**bathocupSani**)$_2BF_4$.

No.	Wavelength (nm)	Oscillator strength *(f)*	Major Contribution
S4	651.4	0.140	328 → 329 (49 %)<br>327 → 330 (49 %)
S5	651.39	0.139	327 → 329 (50 %)<br>328 → 330 (47 %)
S7	595.18	0.061	327 → 332 (49 %)<br>328 → 331 (49 %)
S17	473.85	0.028	327 → 334 (52 %)<br>328→ 333 (46 %)
S20	432.78	0.082	324 → 329 (62 %)
S21	432.73	0.076	323 → 329 (63 %)
S22	428.14	0.719	323 → 329 (31 %)<br>324 → 330 (61 %)
S23	428.13	0.707	323 → 330 (62 %)<br>324 → 329 (30 %)
S25	424.07	0.010	320 → 331 (68 %)<br>325 → 331 (10 %)

Table 3. Calculated selected singlet electronic transitions with oscillator strength (*f* > 0.01) of heteroleptic complex Cu(**bathocupSani**)(**DPEphos**)BF_4.

No.	Wavelength (nm)	Oscillator strength *(f)*	Major Contribution
S2	769.93	0.014	313 → 315 (70 %)
S6	512	0.099	309 → 314 (28 %)<br>311 → 314 (60 %)
S11	480.34	0.126	308 → 314 (21 %)<br>309 → 314 (45 %)
S15	448.91	0.019	306 → 314 (31 %)<br>307 → 314 (46 %)
S16	440.84	0.019	307 → 314 (18 %)<br>311 → 315 (50 %)
S17	436.16	0.011	306 → 315 (35 %)<br>307 → 315 (48 %)
S18	426.5	0.013	313 → 320 (70 %)
S19	423	0.464	306 → 314 (32 %)<br>307 → 314 (15 %)<br>308 → 314 (52 %)
S20	399.54	0.272	306 → 314 (41 %)<br>311 → 316 (41 %)
S21	398.21	0.027	313 → 325 (19 %)<br>313 → 327 (58 %)
S22	390.28	0.043	313 → 321 (59 %)
S23	389.94	0.114	311 → 316 (31 %)<br>313 → 321 (34 %)
S25	381.04	0.351	310 → 316 (31 %)<br>311 → 316 (29 %)
S26	375.91	0.077	310 → 316 (54 %)
S29	371.8	0.065	306 → 315 (50 %)<br>310 → 316 (21 %)<br>313 → 322 (11 %)
S30	369.55	0.020	313 → 330 (12 %)<br>313 → 331 (54 %)<br>313 → 332 (14 %)

Table 4. Atomic coordinates of the optimized geometry of homoleptic complex Cu(**bathocupSani**)$_2$BF$_4$

Atomic Symbol	Coordinates (Å)		
	X	Y	Z
C	-2.5959	1.7649	-1.6865
C	-2.6408	0.9128	-0.6405
C	-1.4628	0.6227	-0.0709
N	-0.2847	0.8439	-0.4882
C	-0.2109	1.5529	-1.5594
C	-1.3431	2.0633	-2.0994
C	-3.7036	0.3046	-0.0818
C	-3.5881	-0.4729	1.0096
C	-2.4206	-0.6103	1.6655
C	-1.4018	0.0918	1.1518
C	-2.1433	-1.3134	2.7809
C	-0.8833	-1.126	3.2342
C	0.0538	-0.2901	2.7064
N	-0.2707	0.3788	1.6553
C	0.2326	3.1996	1.4362
N	0.8019	2.2149	0.8274
C	2.0659	2.2667	0.7169
C	2.8683	3.3346	0.8443
C	2.2916	4.4645	1.2985
C	0.9836	4.3181	1.6072
C	2.5377	1.0555	0.4073
C	3.8198	0.8425	0.0786
C	4.5866	1.9471	0.0275
C	4.1308	3.1493	0.4173
N	1.5971	0.211	0.5046
C	1.8087	-0.9471	-0.0269
C	3.0866	-1.2403	-0.38
C	4.1507	-0.4132	-0.2822
C	-3.6761	2.3326	-2.2825
C	-2.9706	-2.2009	3.3913
C	2.921	5.6359	1.574
C	5.4093	-0.8396	-0.5605
C	-3.6834	2.7041	-3.5819
C	-4.747	3.2805	-4.1649
C	-5.8542	3.5313	-3.4545
C	-5.8706	3.2069	-2.1554
C	-4.799	2.6269	-1.592
C	-3.877	-2.931	2.707
C	-4.6837	-3.8297	3.2933
C	-4.6081	-4.0401	4.6135
C	-3.706	-3.3497	5.3228
C	-2.9034	-2.4604	4.716

C	2.282	6.8239	1.4882
C	2.884	7.992	1.7638
C	4.163	8.0146	2.1604
C	4.8182	6.8533	2.2846
C	4.2007	5.6956	1.9999
C	5.6681	-1.8334	-1.4387
C	6.9144	-2.2564	-1.7054
C	7.9631	-1.7077	-1.0788
C	7.7405	-0.7425	-0.1776
C	6.4878	-0.3294	0.0722
Cu	0.4518	0.9189	0.6497
C	0.9411	1.62	-2.2818
C	1.259	2.291	-3.4068
C	2.4495	2.2794	-4.0504
C	1.1653	-0.2177	3.4938
C	2.1646	0.6822	3.5343
C	3.2917	0.6795	4.2785
C	0.8752	-1.9321	-0.161
C	0.9378	-3.1597	-0.7179
C	-0.0509	-4.082	-0.785
C	-1.0728	3.264	1.8226
C	-1.7789	4.2145	2.4708
C	-3.0972	4.1936	2.7783
C	3.5328	1.5713	-3.6808
C	4.6882	1.6158	-4.3641
C	4.8616	2.3649	-5.4728
C	3.7687	3.0677	-5.8377
C	2.6109	3.0287	-5.1577
C	-1.2948	-3.9378	-0.2915
C	-2.2236	-4.9027	-0.3952
C	-1.9958	-6.0888	-0.9964
C	-0.7468	-6.2227	-1.4893
C	0.187	-5.2623	-1.3891
C	4.1466	1.7149	4.1749
C	5.2934	1.7703	4.8706
C	5.6808	0.7999	5.7246
C	4.8151	-0.2318	5.8177
C	3.6659	-0.2963	5.1243
C	-3.6401	5.2377	3.4339
C	-4.9411	5.284	3.7645
C	-5.8114	4.2957	3.47
C	-5.2578	3.2543	2.8145
C	-3.9573	3.2017	2.483
N	5.9675	2.4034	-6.1209
C	7.1253	1.5979	-5.6503
C	6.0792	3.2535	-7.3351
N	-2.8883	-7.0048	-1.0899
C	-2.5597	-8.2848	-1.7704
C	-4.2411	-6.7764	-0.5159

N	6.7774	0.8535	6.3873
C	7.1361	-0.2562	7.3091
C	7.6786	2.0249	6.226
N	-7.0538	4.3407	3.7844
C	-7.9466	3.2087	3.4215
C	-7.5872	5.5243	4.5086
H	-1.2504	2.7191	-2.9713
H	-4.7103	0.3792	-0.5209
H	-4.5241	-0.9107	1.3874
H	-0.6018	-1.721	4.1176
H	0.5252	5.1849	2.0944
H	5.6077	1.928	-0.3835
H	4.8312	3.9896	0.2996
H	3.3091	-2.259	-0.7117
H	-2.8314	2.5069	-4.2529
H	-4.7169	3.5456	-5.2356
H	-6.7273	4.0127	-3.925
H	-6.7583	3.4454	-1.5448
H	-4.8666	2.4759	-0.5028
H	-3.9486	-2.8855	1.6096
H	-5.3923	-4.4191	2.6866
H	-5.263	-4.7791	5.1036
H	-3.6305	-3.5127	6.4116
H	-2.2162	-1.9154	5.3842
H	1.2401	6.9002	1.1335
H	2.3301	8.9404	1.6575
H	4.6612	8.9693	2.3965
H	5.8596	6.8519	2.6493
H	4.7845	4.7864	2.2126
H	4.8726	-2.3175	-2.0283
H	7.082	-3.0542	-2.4491
H	8.988	-2.0559	-1.2879
H	8.5896	-0.3077	0.3768
H	6.3853	0.4088	0.8845
H	1.7632	1.0042	-1.9035
H	0.4921	2.9387	-3.8578
H	1.2755	-0.9868	4.2751
H	2.0433	1.5651	2.9042
H	-0.1151	-1.7317	0.2522
H	1.8814	-3.4716	-1.1896
H	-1.707	2.4186	1.5605
H	-1.2551	5.122	2.8049
H	3.5321	0.9157	-2.7973
H	5.4951	0.9889	-3.9503
H	3.7644	3.7226	-6.7237
H	1.7789	3.6458	-5.5422
H	-1.6191	-3.0214	0.2246
H	-3.2005	-4.6565	0.0504
H	-0.4183	-7.1383	-2.0067

H	1.1781	-5.4783	-1.8273
H	3.927	2.5614	3.4998
H	5.9009	2.6717	4.6905
H	5.0078	-1.0955	6.4744
H	3.0496	-1.1963	5.2775
H	-3.0178	6.104	3.7224
H	-5.2522	6.1966	4.2979
H	-5.8492	2.3756	2.5097
H	-3.6416	2.2924	1.9509
H	8.0046	1.742	-6.3161
H	7.4592	1.9172	-4.6367
H	6.9013	0.5071	-5.6781
H	5.3754	2.9204	-8.1316
H	5.9362	4.3311	-7.0923
H	7.096	3.1768	-7.7799
H	-3.4365	-8.9693	-1.7711
H	-2.3031	-8.1221	-2.842
H	-1.7488	-8.8323	-1.2382
H	-4.8927	-7.6641	-0.6733
H	-4.197	-6.6268	0.5871
H	-4.7593	-5.9284	-1.0191
H	8.1103	-0.0576	7.8078
H	6.3921	-0.3595	8.1316
H	7.2687	-1.2156	6.759
H	8.5825	1.9202	6.8658
H	8.0565	2.1072	5.1815
H	7.1843	2.9676	6.5542
H	-8.0092	3.0772	2.3171
H	-7.634	2.2657	3.9252
H	-8.988	3.4012	3.7616
H	-7.4882	6.4523	3.9007
H	-8.6754	5.4089	4.7081
H	-7.1081	5.6407	5.5074

Table 5. Atomic coordinates of the optimized geometry of homoleptic complex Cu(**bathocupSani**)(**DPEPhos**)BF_4

Atomic Symbol	Coordinates (Å)		
	X	Y	Z
C	-4.0215	1.1708	0.5659
C	-3.9734	0.6118	1.7862
C	-2.8008	0.4765	2.4306
C	-1.7083	0.9936	1.8434
C	-1.7038	1.3387	0.5491
C	-2.8811	1.4582	-0.0832
O	-2.6372	-0.3092	3.5517
C	-2.2684	-1.5278	3.0033
C	-3.0642	-2.6123	3.0049

C	-2.7236	-3.7205	2.3246
C	-1.6227	-3.7487	1.5536
C	-0.8077	-2.6824	1.515
C	-1.1166	-1.6568	2.3142
P	0.0064	1.5349	-0.2252
P	0.7795	-2.4588	0.4863
C	3.0261	0.2144	0.1496
N	2.2468	-0.2907	-0.7434
C	2.5411	-0.0851	-1.9576
C	3.6401	0.4855	-2.4737
C	4.5486	0.9383	-1.5862
C	4.1781	0.7762	-0.296
C	1.5193	-0.4935	-2.7149
C	1.5449	-0.4269	-4.0531
C	2.6815	0.0615	-4.5828
C	3.7063	0.4757	-3.8179
N	0.5709	-0.8614	-1.9593
C	-0.5946	-0.9458	-2.4981
C	-0.6333	-0.9591	-3.8563
C	0.4136	-0.7845	-4.6922
C	2.7934	0.2055	1.4946
C	3.5203	0.685	2.5263
C	3.1949	0.6964	3.8399
C	-1.7626	-1.1432	-1.8234
C	-3.0267	-1.1828	-2.2976
C	-4.1691	-1.4662	-1.6333
C	5.697	1.606	-1.8699
C	0.278	-0.9183	-6.0363
C	2.024	0.2793	4.3572
C	1.7738	0.3252	5.6747
C	2.6629	0.791	6.5707
C	3.8282	1.2219	6.0515
C	4.0829	1.1778	4.7337
C	6.7841	1.5217	-1.0706
C	7.9261	2.1798	-1.3265
C	8.0202	2.9754	-2.3996
C	6.9529	3.1056	-3.1979
C	5.8214	2.4355	-2.9273
C	1.2917	-1.3618	-6.8103
C	1.1781	-1.5185	-8.1387
C	0.02	-1.2424	-8.7519
C	-1.0156	-0.8259	-8.0119
C	-0.8841	-0.6798	-6.6836
C	-4.2351	-1.8489	-0.3461
C	-5.4014	-2.1211	0.261
C	-6.5837	-2.033	-0.3766
C	-6.5128	-1.6559	-1.6661
C	-5.35	-1.3869	-2.2789
Cu	0.916	-0.5064	-0.7128

C	-0.0739	3.4214	0.2533
C	-1.058	4.2153	-0.2076
C	-1.1061	5.5167	0.1165
C	-0.1603	6.0493	0.9038
C	0.8351	5.2738	1.3575
C	0.8785	3.9728	1.0303
C	0.3779	2.2681	-1.929
C	1.5494	2.8915	-2.1492
C	1.833	3.4101	-3.3538
C	0.947	3.3076	-4.3555
C	-0.2271	2.695	-4.1433
C	-0.5093	2.1839	-2.935
C	1.3212	-3.8291	1.7871
C	1.298	-5.1458	1.5144
C	1.6731	-6.0415	2.4413
C	2.0754	-5.6311	3.6534
C	2.1011	-4.3201	3.9355
C	1.7254	-3.4282	3.0064
C	0.814	-3.563	-1.0523
C	-0.3198	-4.0192	-1.6128
C	-0.2835	-4.7777	-2.719
C	0.8933	-5.0885	-3.2822
C	2.0307	-4.6375	-2.733
C	1.9894	-3.879	-1.6268
O	2.3232	0.8047	7.9021
C	3.2454	1.3274	8.8301
O	-7.8139	-2.284	0.1815
C	-7.8797	-2.6104	1.5506
H	-4.9853	1.2396	0.0335
H	-4.8914	0.1908	2.2286
H	-0.7623	0.9707	2.4023
H	-2.9305	1.75	-1.1423
H	-4.0285	-2.5741	3.5383
H	-3.4005	-4.5916	2.3345
H	-1.4123	-4.6485	0.956
H	-0.3665	-0.866	2.3122
H	0.7737	1.4736	1.0115
H	2.236	-2.3186	0.6453
H	4.8504	1.2302	0.4387
H	2.8066	0.2104	-5.6658
H	4.5947	0.825	-4.363
H	-1.5818	-1.1955	-4.346
H	1.8601	-0.2602	1.8307
H	4.4993	1.1362	2.3108
H	-1.6881	-1.2978	-0.7388
H	-3.1917	-0.9482	-3.3591
H	1.2074	-0.107	3.732
H	0.7944	-0.0297	6.0414
H	4.6189	1.6291	6.7018

H	5.0684	1.5536	4.4049
H	6.8079	0.8604	-0.1878
H	8.7961	2.0623	-0.6581
H	8.9517	3.5256	-2.6117
H	7.0001	3.7894	-4.0626
H	4.9693	2.6704	-3.5829
H	2.249	-1.6906	-6.374
H	2.0289	-1.9013	-8.7277
H	-0.0839	-1.3721	-9.8417
H	-1.9768	-0.5963	-8.5028
H	-1.7785	-0.2988	-6.1641
H	-3.3289	-1.9554	0.2643
H	-5.3585	-2.4234	1.3186
H	-7.4507	-1.5646	-2.2411
H	-5.4011	-1.0862	-3.3402
H	-1.8433	3.8196	-0.8694
H	-1.9203	6.1523	-0.2705
H	-0.1953	7.1193	1.168
H	1.6225	5.7091	1.9957
H	1.7221	3.3748	1.4135
H	2.2969	2.9809	-1.3438
H	2.7984	3.915	-3.5231
H	1.1825	3.7251	-5.3484
H	-0.959	2.6071	-4.9636
H	-1.4789	1.6851	-2.7902
H	0.9689	-5.5216	0.5338
H	1.6484	-7.1193	2.2077
H	2.3822	-6.3676	4.4146
H	2.43	-3.9755	4.9303
H	1.754	-2.3572	3.2588
H	-1.3028	-3.7689	-1.1865
H	-1.2226	-5.1378	-3.1716
H	0.925	-5.7058	-4.1952
H	3.0005	-4.8841	-3.1968
H	2.941	-3.5132	-1.2073
H	2.786	1.2583	9.8417
H	4.179	0.7218	8.8312
H	3.4471	2.4001	8.6128
H	-8.9517	-2.751	1.8156
H	-7.3463	-3.5673	1.747
H	-7.4751	-1.7765	2.167
